# Molecular Data Reveal a Cryptic Diversity in the Genus *Urotricha* (Alveolata, Ciliophora, Prostomatida), a Key Player in Freshwater Lakes, With Remarks on Morphology, Food Preferences, and Distribution

**DOI:** 10.3389/fmicb.2021.787290

**Published:** 2022-02-04

**Authors:** Daniela Frantal, Sabine Agatha, Daniela Beisser, Jens Boenigk, Tatyana Darienko, Gianna Dirren-Pitsch, Sabine Filker, Michael Gruber, Barbara Kammerlander, Laura Nachbaur, Ulrike Scheffel, Thorsten Stoeck, Kuimei Qian, Birgit Weißenbacher, Thomas Pröschold, Bettina Sonntag

**Affiliations:** ^1^Research Department for Limnology, Mondsee, University of Innsbruck, Mondsee, Austria; ^2^Department of Biosciences, Paris Lodron University of Salzburg, Salzburg, Austria; ^3^Department of Biodiversity, University of Duisburg-Essen, Essen, Germany; ^4^Experimental Phycology and Culture Collection of Algae, University of Göttingen, Göttingen, Germany; ^5^Limnological Station, Department of Plant and Microbial Biology, University of Zurich, Kilchberg, Switzerland; ^6^Molecular Ecology Group, Technische Universität Kaiserslautern, Kaiserslautern, Germany; ^7^Hieronymus-Illustrations, Salzburg, Austria; ^8^Federal Agency for Water Management, Institute for Aquatic Ecology and Fisheries Management, Mondsee, Austria; ^9^Ecology Group, Technische Universität Kaiserslautern, Kaiserslautern, Germany; ^10^College of Environmental Engineering, Xuzhou University of Technology, Xuzhou, China

**Keywords:** ciliate plankton, biogeography, ciliate diversity, cryptic species, microplankton

## Abstract

Species of the ciliate genus *Urotricha* are key players in freshwater plankton communities. In the pelagial of lakes, about 20 urotrich species occur throughout an annual cycle, some of which play a pivotal role in aquatic food webs. For example, during the phytoplankton spring bloom, they consume a remarkable proportion of the algal production. In ecological studies, urotrich ciliates are usually merely identified to genus rank and grouped into size classes. This is unsatisfying considering the distinct autecological properties of individual species and their specific spatial and temporal distribution patterns. As a basis for future research, we characterized in detail four common urotrich morphotypes, i.e., specimens identified as *U. furcata* and tentatively as *U. agilis*, *U. pseudofurcata*, and *U. castalia*, using state-of-the-art methods. We used an integrative polyphasic approach, in which morphological studies (*in vivo* observation, silver staining methods, scanning electron microscopy) were linked with a molecular approach exploiting four different gene fragments as taxonomic DNA barcodes with different resolution potential (SSU rDNA, ITS-1, ITS-2, hypervariable V4 and V9 regions of the SSU rDNA). We shed light on the diversity of urotrich ciliates as well as on their global distribution patterns, and annual cycles. Additionally, we coupled individual species occurrences and environmental parameters, and subsequently modeled the distribution and occurrence, using logistic regressions. Furthermore, for one strain putatively identified as *U. castalia*, we ascertained the optimal cultivation media and food preferences. Thereby, our comprehensive view on these important freshwater ciliates that frequently occur in environmental high throughput sequencing datasets worldwide will allow future studies to better exploit protistan plankton data from lakes.

## Introduction

Members of the genus *Urotricha* Claparède and Lachmann, 1859 represent some of the most common planktonic ciliates in freshwater systems worldwide, including temperate and remote lakes, and reservoirs (e.g., Weisse et al., 1990; Sonntag et al., 2006, 2017; Sichrowsky et al., 2014; Posch et al., 2015; Kammerlander et al., 2016; Šimek et al., 2019). *Urotricha* species often dominate ciliate communities and co-occur with other small prostomatids, such as *Balanion planctonicum*, halteriids, and diverse oligotrichs, e.g., *Rimostrombidium* species. Species of this genus are considered omnivores with a preference for algae. Accordingly, they significantly foster the clear-water phase after the algal spring bloom in temperate lakes (Weisse et al., 1990; Foissner et al., 1999; Sonntag et al., 2006; Tirok and Gaedke, 2006). In ecological network analyses, certain *Urotricha* species have been identified as key ciliates, and significant connections to other planktonic organisms suggested that urotrichs were indeed omnivorous feeders (Qu et al., 2021). Omnivory might be a nutritional advantage for the survival of a species (Holyoak and Sachdev, 1998; Diehl, 2003); it may not only cause positive growth rates, but also contribute to the stability of a ciliate community due to the numerous prey options. Despite several experiments concerning their ecological niche and the quality of their food items, the knowledge on the autecology of urotrichs is still insufficient (e.g., Weisse et al., 2001; Weisse and Frahm, 2001, 2002). In principle, the role of ciliates in the planktonic food web, including predators and algae, is largely a black box, though ciliate grazing on phytoplankton contributes significantly to the carbon flux. Ciliate growth and grazing rates largely depend on the quantity and quality of available food and are species-specific. For a better understanding of the role of ciliates within the planktonic food web, investigations on a large variety of predator-prey relationships are needed.

Despite their wide distribution and occasionally high abundance in the ciliate plankton, merely environmental or unidentified sequences of urotrichs have been deposited in public databases. The lack of barcodes of reliably identified *Urotricha* species is probably due to the difficulties in observing and identifying these very small ciliates with their characteristic fast forward swimming interrupted by rapid jumps. Additionally, their cultivation is tricky because appropriate food items and media need to be tested and adapted for each particular strain (e.g., Weisse et al., 2001). So far, approximately 40 urotrich species have been described based on live observation, silver-stained material, and partially scanning electron microscopy (for a compilation, see Foissner and Pfister, 1997; Foissner et al., 1999; Sonntag and Foissner, 2004; Foissner, 2012; Supplementary Table 1). The morphospecies are pragmatically classified according to the numbers of their caudal cilia, i.e., those with one or two cilia in one group and those with three or more cilia in the other group (Foissner et al., 1999). While urotrichs of the latter group are treated by identification keys (Foissner and Pfister, 1997; Foissner et al., 1999), the determination of smaller congeners (less than 30 μm in size) with one or two caudal cilia usually requires the consultation of the scattered original descriptions.

An increasing number of next generation sequencing studies in freshwater habitats produces huge amounts of sequence data, whose annotation depends on the completeness of the reference database. Accordingly, there is an urgent need for populating these databases with taxonomic marker genes (taxonomic DNA barcodes) of morphologically identified *Urotricha* species (Stoeck et al., 2014a,b; Pitsch et al., 2019; Qu et al., 2021). Such high throughput sequencing datasets of freshwater ciliate communities comprise mainly the hypervariable V4 or V9 regions of the small subunit ribosomal DNA (SSU rDNA) as taxonomic marker genes (e.g., Stoeck et al., 2014b; Boenigk et al., 2018; Pröschold et al., 2021; Qu et al., 2021). Particularly, the V4 region has a highly similar phylogenetic resolution as the entire SSU rDNA fragment (Dunthorn et al., 2012). While the SSU rDNA has a resolution at generic and higher taxonomic levels, the large subunit rDNA (LSU) or the internal transcribed spacer sequences (ITS-1 and ITS-2, respectively) are even capable to distinguish congeners (Coleman, 2000, 2003; Pröschold et al., 2021). For instance, the ITS-2 is particularly suitable for differentiating species in the ciliate genera *Paramecium*, *Tetrahymena*, and *Spirostomum* (Coleman, 2000, 2005; Shazib et al., 2016; Zhan et al., 2019). Compensatory base exchanges (CBCs) in the conserved region of the ITS-2, which form pairings in the secondary structure, turned out to coincide with sexual incompatibility, meeting the criteria of the biological species concept (Coleman, 2000, 2003). The choice of markers appropriate for resolving the phylogenetic relationships is, however, taxon-specific (Dunthorn et al., 2012; Stoeck et al., 2014a; Tanabe et al., 2016; Zhan et al., 2019).

The aim of this study was to integrate morphological and molecular data for common and ecologically relevant *Urotricha* species. In particular, representatives of the two *Urotricha* groups were investigated, namely, specimens less than 30 μm in size with one or two caudal cilia and specimens more than 30 μm in size with more than two caudal cilia. For the latter, we additionally searched for appropriate cultivation media and prey items to detect potential food preferences in one of the strains. This integrative approach will provide the basis for future ecological research on this important group of ciliates.

## Materials and Methods

### Origin of the *Urotricha* Strains and Lake Sampling

The *Urotricha* species were collected in three oligo-mesotrophic lakes (Lake Mondsee, Lake Piburg, and Lake Zurich) located in Austria and Switzerland (Table 1). Details on these lakes can be found elsewhere (e.g., Bossard et al., 2001; Dokulil et al., 2006; Sonntag et al., 2011; Ficker et al., 2017; Yankova et al., 2017; Niedrist et al., 2018). Sampling was conducted by boat at the deepest point of each lake (68 m in Lake Mondsee, 25 m in Lake Piburg, and 136 m in Lake Zurich) and the water samples were collected along depth gradients from the surface to above lake bottom by means of a 5-L Schindler-Patalas sampler (Uwitec, Mondsee, Austria) and a plankton net (10-μm net gauze; Uwitec, Mondsee, Austria). Samples were transported in 1-L plastic bottles at ambient lake water temperatures to the laboratory and processed right after sampling.

**TABLE 1:** Characteristics of the study sites (e.g., Bossard et al., 2001; Dokulil et al., 2006; Sonntag et al., 2011; Ficker et al., 2017; Yankova et al., 2017; Niedrist et al., 2018).

	Lake Mondsee	Lake Zurich	Lake Piburg
Coordinates	47°48′28.0′′ N	47°17′08.8′′ N	47°11′42.0′′ N
	13°23′15.0′′ E	08°35′27.6′′ E	10°53′20.0′′ E
Maximum depth (m)	68.3	136.0	24.6
Surface area (km^2^)	14.2	66.8	0.17
Catchment area (km^2^)	247	1,740	1.34
Water retention time (years)	1.7	1.4	1.6
Mixis type	dimictic	monomictic	dimictic
Trophic status	oligo-mesotrophic	oligo-mesotrophic	oligo-mesotrophic

### Cultivation and Cloning Procedures

Mixed cultures were set up from raw water samples enriched with algal food, i.e., SAG 26.80 *Cryptomonas* sp. (Culture Collection of Algae at the University of Göttingen, Germany^1^). For cloning, we picked individual urotrichs with finely drawn glass pipettes under a stereo microscope (Olympus SZ61, Olympus, Vienna, Austria) at up to 45 × magnification. A subsequent purification procedure following Pringsheim (1946) appeared to be crucial for both the molecular analyses and the cultivation of clonal strains. Single urotrich specimens were cultivated in a 5:1 mixture of modified Woods Hole MBL medium (WC; Guillard and Lorenzen, 1972) and Volvic^®^ mineral water, a previously tested cultivation medium (see below). Food algae were kept in modified Bold’s Basal Medium (3NBBM; medium 26a in Schlösser, 1997). All cultures were grown at temperatures of 15–21°C under a light:dark cycle of 12:12 h with a photon flux rate of 50 μmol m^–2^s^–1^. Successfully grown clones were subsequently transferred from the small volumes of 96-well plates into 24-, 12-, and 6-well plates and finally into 50-mL cultivation flasks with a ventilated cap (Biomedica).

### Search for Optimal Media and Food

Specifically for the strain CIL-2017/25, tentatively identified as *U. castalia*, we searched for optimal cultivation media and food items. We inoculated five individuals of *U. castalia* each in a well of a 12-well plate (in triplicates) containing one of the following media: sterile-filtered original lake water (0.2-μm filter; Minisart, Sartorius), Volvic^®^ mineral water, modified Bourrelly medium (Hindák, 1970), WC medium only, 1:1, 1:5, 5:1 v/v mixtures of WC:Volvic^®^, and modified blue-green medium (BG11; Rippka and Herdman, 1992). For testing food preferences, diverse algae were either successfully isolated and cultivated from Lake Mondsee (indicated by “MS” with a strain number) or obtained from a public culture collection (Supplementary Figure 1). In the feeding experiment, we tested the strains MS-2017/1 *Coelastrum* sp., MS-2017/2 *Choricystis* sp., MS-2017/7 *Acutodesmus obliquus*, MS-2017/8 *Cosmarium* sp., and SAG 26.80 *Cryptomonas* sp. in 12-well culture plates with WC:Volvic^®^ medium 5:1 v/v (triplicates). The cultures of the ciliate and the respective algae were subsequently monitored by direct live counts in the plates under a stereomicroscope (Olympus SZ61) at up to 45 × magnification.

### Morphological Investigations

The morphology of the *Urotricha* strains was studied by applying standard methods for revealing the species-specific cell features and producing the drawings (Foissner, 1991, 2014). Living ciliates and algae (for the feeding experiments) were examined under differential interference contrast optics at 40–1,000 × magnification by means of an Olympus BX51 microscope (Olympus, Vienna, Austria). Two digital image analysis systems were employed for documentation and measurements (Jenoptik ProgRes SpeedXT core 5 2.9.0.1., Jenoptik PROGRES Gryphax^®^ Arktur). Additionally, measurements were done by a calibrated eyepiece micrometer. Regarding protargol staining, we applied Foissner’s (1991, 2014) protocol and Skibbe’s (1994) quantitative method (QPS). The specimens were identified by means of the keys published by Foissner and Pfister (1997) and Foissner et al. (1999).

For scanning electron microscopy, the specimens were fixed for 30 min in a modified Párducz’s solution (Párducz, 1967; 4 parts 2% osmium tetroxide and 1 part saturated aqueous mercuric-chloride). After three washing steps in 0.1 M sodium cacodylate buffer, the material was transferred into preparation chambers equipped with Millipore nets of 10-μm mesh size, following the method of Foissner (1991, 2014). After dehydration by an ascending ethanol series (30-50-70-90-96-3 × 100%, 5 min each), critical point drying was performed in a BAL-TEC CPD030. The material was sputter-coated with gold in an AGAR Sputter coater and viewed in a Philips ESEM XL-30.

### Molecular Methods

To receive the DNA of the studied ciliates, we used the isolation method described by Pringsheim (1946). Approximately ten ciliate cells per strain were washed, one specimen each was placed into a well containing fresh medium and starved for a strain-specific period of time (Table 2). The whole genome amplification of single cells was performed, using the REPLI-g^®^ Single Cell method (Qiagen, Catalog no. 150343, Hilden, Germany). The used approach followed the protocol provided by the manufacturer and is briefly described as follows: a single cell was added to 4 μL of phosphate-buffered saline solution (PBS, Qiagen) in precooled 0.2-mL PCR tubes (StarLab). After adding 3 μL of D2 buffer (Qiagen) and flicking, the tubes were incubated for 10 min at 65°C in a Mastercycler (Thermal Mastercycler ^®^nexus gradient, Eppendorf). Afterward, 3 μL of the Stop Solution (Qiagen) and 40 μL of the Mastermix (9 μL H_2_O, 29 μL REPLI-g^®^ Reaction Buffer, 2 μL REPLI-g^®^ DNA Polymerase) were added into the PCR-tube. Finally in the Mastercycler, the following PCR program was applied: 8 h at 30°C and 3 min at 65°C. An aliquot of the 50-μL-mix (1 μL) was taken and diluted 1:10 with H_2_O. Subsequently, 1 μL of the diluted mix was again diluted to obtain a 1:100 dilution. The 1:100 diluted DNA was used to amplify the targeted short DNA regions comprising the V4 and V9 regions of the SSU rDNA, ITS-1, and ITS-2 fragments in two independent PCR-reactions for obtaining the near complete SSU rDNA and ITS sequences with an overlap of approximately 180 bp of both PCR-products. The first part of the SSU rDNA (PCR1) was amplified, using the forward eukaryote-specific primer EAF3 (5′TCGACAATCTGGTTGATCCTGCCAG3′; Marin et al., 2003) and the reverse ciliate-specific primer CilR, which was actually a mix of three primers (CilR1 - 5′TCTG ATCGTCTTTGATCCCTTA, CilR2 - 5′TCTRATCGTCTTTG ATCCCCTA3′, and CilR3 - 5′TCTGATTGTCTTTGATCCCC TA3′; Lara et al., 2011). For the second part of the SSU and the ITS (PCR2), we used the ciliate-specific primer CilF (5′TGGTAGTGTATTGGACWACCA3′; Lara et al., 2007) and the eukaryote-specific primer ITS055R (5′CTCCTTGGT CCGTGTTTCAAGACGGG3′; Marin et al., 2003). Both PCR reactions were conducted in a volume of 50 μL containing 25 μL Taq-PCR-Mastermix (Qiagen, Catalog no. 201445, Hilden, Germany), 24 μL H_2_O, and 0.5 μL of 10 μM of each primer, and were performed in duplicates. The following program was applied for both PCR reactions in an Eppendorf Mastercycler nexus gradient (Eppendorf, Wesseling-Berzdorf, Germany): 5 min initial denaturation at 96°C followed by 30 cycles each comprising 1 min denaturation at 96°C, 2 min primer annealing at 55°C, and 3 min elongation at 68°C, and lastly 10 min final elongation at 68°C. The resulting PCR products were purified, using the Qiagen PCR Purification Kit (Qiagen, Catalog no. 28106, Hilden, Germany) according to the manufacturer’s protocol, and sequenced (Sanger approach), using the primers N82F and 536R for PCR1, and E528F, N920F, N920R, BR, GF, and GR for PCR2 (for primer sequences see Darienko et al., 2019). The resulting sequences of these eight reactions were assembled and corrected into one contig for each *Urotricha* strain.

**TABLE 2:** *Urotricha* strains based on molecular sequences (all strains) and on morphology (marked with an *): species assignment, strain number, isolation details (location, date, collector), cultivation conditions, maximum starvation time before sequencing.

Species	Strain number	Origin, date of isolation, collector	Cultivation medium, temperature	Starvation time (max. min)
*Urotricha agilis*	CIL-2019/8	Lake Piburg, 2011, BS	WC/Volvic^®^ 5:1, 21°C	105
	CIL-2019/9	Lake Piburg, 2011, BS	WC/Volvic^®^ 5:1, 21°C	45
	*CIL-2019/10	Lake Piburg, 2011, BS	WC/Volvic^®^ 5:1, 21°C	20
	CIL-2017/23	Lake Mondsee, 10/31/2017, BK	WC/Volvic^®^ 5:1, 15°C	240
	*CIL -2017/24	Lake Mondsee, 10/31/2017, BK	WC/Volvic^®^ 5:1, 15°C	240
*Urotricha furcata*	CIL-2019/5	Lake Zurich, 2011, BS	WC/Volvic^®^ 5:1, 21°C	80
	*CIL-2019/6	Lake Zurich, 2011, BS	WC/Volvic^®^ 5:1, 21°C	90
	CIL-2019/7	Lake Zurich, 2011, BS	WC/Volvic^®^ 5:1, 21°C	90
	*CIL-2019/13	Lake Zurich, 2011, BS	WC/Volvic^®^ 5:1, 21°C	90
	CIL-2019/14	Lake Zurich, 2011, BS	WC/Volvic^®^ 5:1, 21°C	20
	CIL-2019/15	Lake Zurich, 2011, BS	WC/Volvic^®^ 5:1, 21°C	10
*Urotricha pseudofurcata*	*CIL-2019/3	Lake Zurich, 2014, GD	WC/Volvic^®^ 5:1, 21°C	0
	CIL-2019/4	Lake Zurich, 2014, GD	WC/Volvic^®^ 5:1, 21°C	0
*Urotricha castalia*	*CIL-2019/1	Lake Zurich, 2015, GD	WC/Volvic^®^ 5:1, 21°C	240
	CIL-2019/2	Lake Zurich, 2015, GD	WC/Volvic^®^ 5:1, 21°C	240
	*CIL-2017/25	Lake Mondsee, 10/31/2017, BK	WC/Volvic^®^ 5:1, 15°C	240
	CIL-2017/26	Lake Mondsee, 10/31/2017, BK	WC/Volvic^®^ 5:1, 15°C	240
	*CIL-2017/27	Lake Mondsee, 10/31/2017, BK	WC/Volvic^®^ 5:1, 15°C	240

*BK, Barbara Kammerlander; BS, Bettina Sonntag; DF, Daniela Frantal; GD, Gianna Dirren-Pitsch; WC, Woods Hole MBL medium.*

### Phylogenetic Analyses

The SSU rDNA sequences were compared, using the BLASTn search approach (Altschul et al., 1990). This approach confirmed the affiliation of the new sequences with the Prostomatea. The dataset of this ciliate class was originally compiled for the genus *Coleps* by Pröschold et al. (2021). The *Urotricha* sequences were manually aligned to the secondary structure presented for CIL-2017/25 (Supplementary Figure 2) and were integrated into two datasets: (1) an SSU dataset of 35 prostomatean taxa (length 1,755 bases) to demonstrate the monophyly of the genus *Urotricha* and its phylogenetic position, and (2) an SSU/ITS dataset of our 18 studied *Urotricha* strains (length 2,343 bases) to get a better phylogenetic resolution within this genus. The automated model selection tool implemented in PAUP version 4.0a167 was employed (Swofford, 2002) to find the best fitting evolutionary model for the datasets. The best models (model settings provided in the figure legends), using the Akaike Information Criterion, were chosen (Akaike, 1974). The following phylogenetic analyses were performed with PAUP: Maximum Likelihood, using the best model estimated by PAUP, Neighbor-Joining, and Maximum Parsimony. To find molecular signatures for supporting the assigned species, the secondary structures of the hypervariable V4 and V9 regions of the SSU rDNA and the ITS-2 were reconstructed, using the program mfold, which used the thermodynamic model for folding (Zuker, 2003). The visualization of the structures was conducted by PseudoViewer3 (Byun and Han, 2006). The compensatory base changes (CBCs) were detected, using the program CBCAnalyzer version 1.1 (Wolf et al., 2005).

### Spatial and Temporal Distribution and Response to Environmental Factors

To investigate the global distribution of the urotrich strains, an in-house reference database was assembled consisting of V4 and V9 datasets from freshwater lakes in Austria, Chile, the Czech Republic, Ethiopia, France, Germany, Hungary, Iceland, Italy, Nepal, Norway, Poland, Romania, Slovakia, Spain, Sweden, and Switzerland. Further information on the datasets can be found in the Supplementary Table 2. The retrieved V4 and V9 sequences were then used as an exact-match query and blasted against the reference database to screen for their presence at a specific location.

Additionally, sequence and cell count data of the *Urotricha* species from Lake Mondsee and Lake Zurich spanning over a 1-year period were accumulated to compare the spatial and temporal distribution within both lakes. Information about the sampling strategy, sequencing, and data analyses can be retrieved from Qu et al. (2021). Based on the QPS method of Skibbe (1994), *Urotricha furcata*, *Urotricha agilis*, *Urotricha castalia*, and *Urotricha pseudofurcata* from Lake Mondsee were morphologically identified and counted and *U. agilis*, *U. castalia*, and *U. pseudofurcata* from Lake Zurich. Contour plots were created with the “plotly” package in R for visualizing the occurrence of the *Urotricha* species in the two lakes.

The available environmental data from the study of the freshwater lakes by Boenigk et al. (2018), were used to identify potential ecological preferences of the ciliate species under study (Supplementary Table 3). Spearman’s rank coefficients were calculated to relate *Urotricha* abundance and environmental data. A multiple logistic regression analysis was used to estimate the effect of environmental parameters on the *Urotricha* abundances in those lakes for which all environmental parameters were available (*n* = 85). All values were log-transformed to achieve additivity of their effects and to increase the homogeneity of variances in the response variable (i.e., *Urotricha* abundances). A stepwise procedure was then used to build the final model based on the Akaike Information Criterion (AIC) and the obtained *p*-values. The final model included those environmental parameters, which influenced the *Urotricha* occurrences. All statistical analyses were conducted with the “stats” package in R version 4.0.5.

## Results

### Morphological Characterization and Species Identification

We successfully isolated and cultivated 18 *Urotricha* strains, which we assigned to *Urotricha furcata* Schewiakoff, 1892 and tentatively to *Urotricha agilis* (Stokes, 1886) Kahl, 1930, *Urotricha pseudofurcata* Krainer, 1995, and *Urotricha castalia* Muñoz et al., 1987 (Table 2 and Supplementary Table 4). In the detailed descriptions of our investigated strains, we focussed on the species-specific features. Common urotrich features are thus not repeatedly mentioned, i.e., (i) the central position of the macronucleus and the micronucleus, (ii) the eccentric position of the single contractile vacuole in the unciliated posterior cell portion, (iii) the eccentric position of the one or two caudal cilia at the rear end of the small species, (iv) the pattern of the somatic ciliature with equidistantly arranged, posteriorly shortened meridional rows leaving blank the posterior cell portion, (v) the dikinetids at the anterior ends of the somatic kineties which have a cilium associated only with the posterior basal body, and (vi) the fast jumping movement (Foissner and Pfister, 1997; Foissner et al., 1999).

**FIGURE 1:**
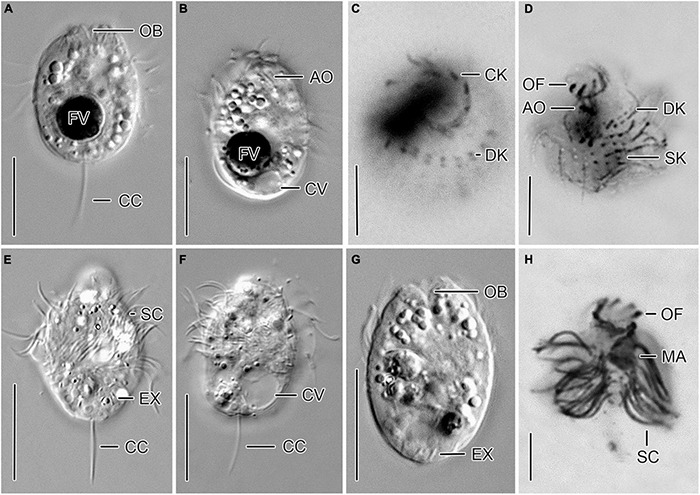
Cryptic species tentatively identified as *Urotricha agilis*. Specimens of strain CIL-2017/24 from Lake Mondsee **(A–D)** and of strain CIL-2019/10 from Lake Piburg **(E–H)** *in vivo* **(A,B,E–G)** and after protargol staining **(C,D,H)**. **(A,B,E–G)** Longitudinal optical sections. **(C,D)** Top view and oblique top view. **(H)** Lateral view. AO, adoral organelles; CC, caudal cilium; CK, circumoral kinety; CV, contractile vacuole; DK, dikinetids at anterior end of somatic kineties; EX, extrusome; FV, food vacuole; MA, macronucleus; OB, oral basket; OF, oral flaps; SC, somatic cilia; SK, somatic kineties. Scale bars 10 μm **(A,B,E–G)**, 5 μm **(C,D,H)**.

**FIGURE 2:**
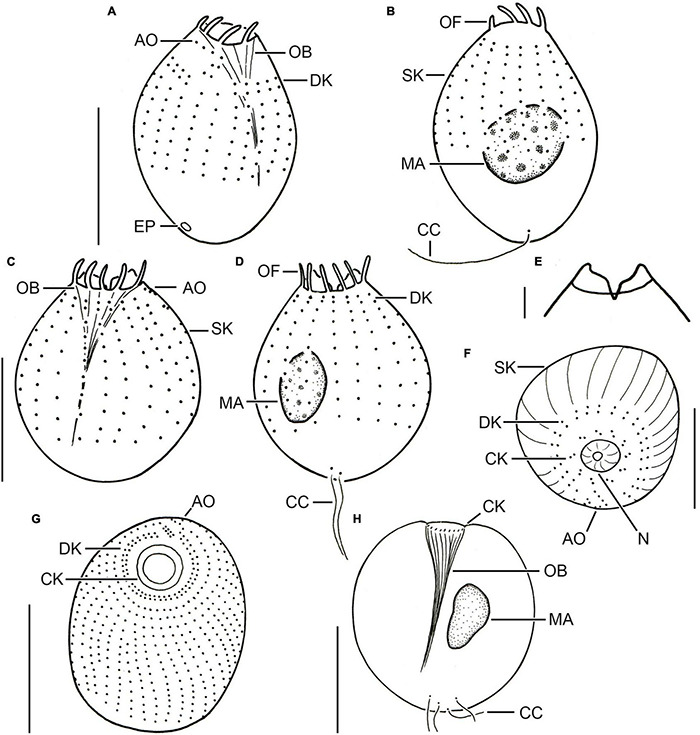
Different urotrich morphotypes after protargol staining. **(A,B)** Ventral and dorsal views of a specimen of strain CIL-2017/24 from Lake Mondsee tentatively identified as *Urotricha agilis.* Occasionally, only the ciliated basal bodies of the adoral organelles stain. **(C–F)** *Urotricha furcata*, specimens of strain CIL-2019/6 from Lake Zurich. Right **(C)** and left **(D)** lateral views of same specimen. Optical section of the protruding oral region **(E)**. Oblique top view **(F)**. **(G,H)** Specimens of strain CIL-2019/1 from Lake Zurich tentatively identified as *U. castalia*. Oblique top view **(G)**; the concentric circles denote the circumoral kinety. Schematic optical section **(H)**. AO, adoral organelles; CC, caudal cilia; CK, circumoral kinety; DK, dikinetids at anterior end of somatic kineties; EP, excretory pore; MA, macronucleus; N, nematodesmata (oral basket rods); OB, oral basket; OF, oral flaps; SK, somatic kineties. Scale bars 10 μm **(A–D,F)**, 2 μm **(E)**, and 20 μm **(G,H)**.

*Urotricha agilis* (Stokes, 1886) Kahl, 1930 (Figures 1A–H, 2A,B, and Supplementary Table 4).

Remarks. The strains CIL-2017/24 and CIL-2019/10 were morphologically and genetically investigated. Despite their morphological similarity, the genetic data (dissimilarity and CBCs) indicate the presence of two cryptic species; hence, their morphometric data are kept separate.

*Urotricha agilis* (Stokes, 1886) Kahl, 1930 - Strain CIL-2017/24 from Lake Mondsee (Figures 1A–D, 2A,B).

Remarks. The strain was studied *in vivo* and after protargol staining; it is genetically highly similar to the strains CIL-2019/8 and CIL-2019/9 from Lake Piburg and strain CIL-2017/23 from Lake Mondsee.

Description. The cell is ellipsoidal to broadly ellipsoidal *in vivo* and in protargol-stained cells. It measures 14–23 × 9–16 μm *in vivo* and 10–16 × 8–14 μm in protargol preparations. The cell length:width ratio is 1.2–1.9:1 *in vivo* and 1.0–1.6:1 in protargol preparations. The unciliated posterior cell portion is not set off plug-like and occupies 17–37%, on average 26% of cell length, being 3–6 μm long in protargol preparations (Figures 2A,B). The globular to broadly ellipsoidal macronucleus measures 3–8 × 3–5 μm *in vivo* and 3–6 × 2–6 μm in protargol preparations; the micronucleus is not recognizable. Since the rod-shaped somatic extrusomes are sparse and merely about 1.5 μm long, they are hardly visible *in vivo*, while not recognizable in protargol preparations. The cytoplasm is colorless and includes refractive lipid droplets 1–3 μm across and often many (colorful) food vacuoles 2–7 μm, on average 5 μm across. The contractile vacuole is up to 8 μm across; its excretory pore is somewhat eccentric (Figure 2A). The somatic cilia are relatively long with 3–6 μm *in vivo* and 3–7 μm in protargol preparations. The somatic ciliature comprises 18–24 kineties consisting of 6–12 cilia in anteriorly unshortened rows. The length of the single caudal cilium is about half the cell length, namely, 6–12 μm *in vivo* and 5–10 μm in protargol preparations (Figures 1A, 2B). The 7–9 oral flaps are 1–4 μm long after protargol preparation (Figures 1C,D, 2A,B). The oral basket is 2–7 μm across distally and 2–6 μm long *in vivo*, while 3–5 μm across distally and 6–12 μm long in protargol preparations, in which the oral basket occupies 29–63%, on average 44% of cell width. The three adoral organelles (“brosse”) are inconspicuous, especially adoral organelles 2 and 3 (Figures 1B,D, 2A): adoral organelle 1 consists of 3-5 dikinetids and is 1.5–2.5 μm in length, adoral organelle 2 consists of two or three dikinetids and is 0.8–1.2 μm in length, and adoral organelle 3 consists of one or two dikinetids and is 0.8-1.0 μm in length. They are placed on a comparatively broad (about 2.5 μm on average) blank stripe, which separates the circumoral kinety and the somatic kineties. Occasionally, only the ciliated basal bodies of the dikinetids constituting the adoral organelles are stained.

*Urotricha agilis* (Stokes, 1886) Kahl, 1930 - Strain CIL-2019/10 from Lake Piburg (Figures 1E–H).

Remarks. The strain was studied *in vivo* and after protargol staining.

Description. The cell is ellipsoidal to broadly ellipsoidal *in vivo* and in protargol-stained cells. It measures 13–19 × 9–14 μm *in vivo* and 9–15 × 6–11 μm in protargol preparations. The cell length:width ratio is 1.1–1.9:1 *in vivo* and 1.0–1.8:1 in protargol preparations. The unciliated posterior cell portion is hardly set off plug-like and occupies 26–48%, on average 37% of cell length, being 3–5 μm long in protargol preparations. The globular macronucleus is 2–6 μm across *in vivo* and measures 2–5 × 1–5 μm in protargol preparations; the micronucleus is not recognizable. Since the rod-shaped somatic extrusomes are sparse and merely about 1.5 μm long, they are hardly visible *in vivo* (Figures 1E,G); in protargol preparations, they are about 1 × 0.2–0.5 μm in size. The cytoplasm is colorless and includes refractive lipid droplets and frequently many (colorful) food vacuoles of 3–6 μm, on average 4 μm across. The contractile vacuole is up to 5 μm across. The somatic cilia are relatively long with 2–6 μm *in vivo* and 4–7 μm in protargol preparations. The somatic ciliature comprises 14–22 kineties consisting of 5–7 cilia in anteriorly unshortened rows. The length of the single caudal cilium is about half the cell length, namely, 5–9 μm *in vivo* and 6–9 μm in protargol preparations (Figures 1E,F). The 8–10 oral flaps are 1.2–2.1 μm long in protargol preparations, in which the circumoral kinety is only incompletely visible (Figure 1H). In protargol preparations, the oral basket is 3–5 μm across distally, and thus comparatively wide, occupying 29–67%, on average 47% of cell width; its length was not measured because it was not visible. The three (*n* = 3) adoral organelles (“brosse”) are obliquely arranged in the blank stripe separating the circumoral kinety and the somatic kineties. Because they are minute, the organelles are even hardly visible in protargol preparations: adoral organelle 1 consists of four or five dikinetids and is 2–3 μm in length, adoral organelle 2 consists of two dikinetids and is about 1 μm in length (*n* = 5), and adoral organelle 3 consists of two dikinetids and is also about 1 μm in length (*n* = 3). The blank stripe is 1.8–3.0 μm broad.

**FIGURE 3:**
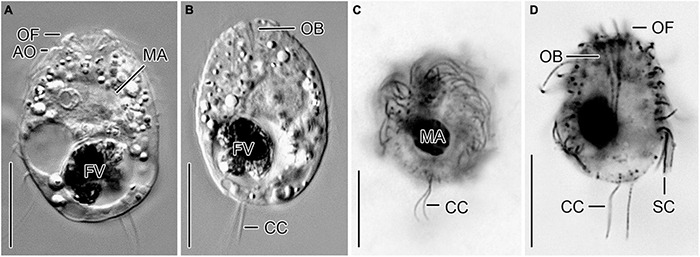
*Urotricha furcata*, specimens of strains CIL-2019/13 *in vivo* **(A,B)** as well as of the non-clonal original culture **(C)** and CIL-2019/6 **(D)** after protargol staining from Lake Zurich. **(A,B,D)** Optical longitudinal sections. **(C)** Oblique posterior polar view. AO, adoral organelles; CC, caudal cilia; FV, food vacuole; MA, macronucleus; OB, oral basket; OF, oral flaps; SC, somatic cilia. Scale bars 10 μm.

*Urotricha furcata* Schewiakoff, 1892 - Strains CIL-2019/6 and CIL-2019/13 (Figures 2C–F, 3A–D, and Supplementary Table 4).

Remarks. Out of the six genetically identical strains, two were morphologically investigated, i.e., CIL-2019/6 was studied in protargol slides and CIL-2019/13 *in vivo*.

Description. The cell is ellipsoidal to broadly ellipsoidal. It measures 17–35 × 10–29 μm *in vivo* and 11–19 × 10–16 μm in protargol preparations. The cell length:width ratio is 1.2–1.9:1 *in vivo* and 0.9–1.7:1 in protargol preparations. The unciliated posterior portion is not set off plug-like and occupies 16–26%, on average 22% of cell length, being 2–4 μm long in protargol preparations (Figures 2C,D, 3A,B). The globular macronucleus measures 4–7 × 3–7 μm *in vivo* and 3–9 × 3–7 μm in protargol preparations; the globular micronucleus is 2–3 μm across *in vivo*, while it is not recognizable in stained specimens. Extrusomes are neither recognizable *in vivo* nor after protargol staining. The cytoplasm is colorless and includes refractive lipid droplets 1–3 μm across and often many (colorful) food vacuoles 2–8 μm, on average 5 μm across. The contractile vacuole is up to 7 μm across. The somatic cilia are relatively long with 3–7 μm *in vivo* and 3–6 μm in protargol preparations. The somatic ciliature comprises 18-24 kineties consisting of 10-14 kinetids in anteriorly unshortened rows. The length of the two caudal cilia is about one third to one half of cell length, namely, 6–11 μm *in vivo* and 4–8 μm in protargol preparations (Figure 2C). The three (*n* = 7) adoral organelles are hardly visible and probably arranged in a V-shaped pattern (Figure 2F): adoral organelle 1 consists of three or four dikinetids and is 1–2.5 μm in length, adoral organelle 2 consists of two or three dikinetids and is about 0.8–1.5 μm in length, and adoral organelle 3 consists of two dikinetids and is about 0.8–1.0 μm in length. The 7-12 oral flaps are 1–3 μm long in protargol preparations. In protargol preparations, the oral basket is 3–6 μm across distally and 4–10 μm long; it occupies 20–40%, on average 30% of cell width (Figures 2C,E).

**FIGURE 4:**
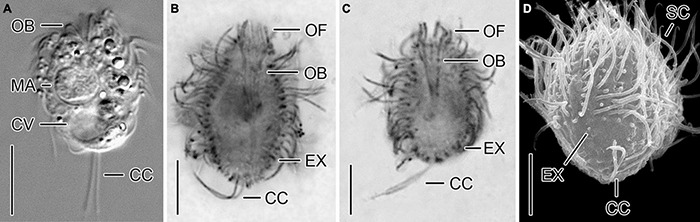
A cryptic species tentatively identified as *Urotricha pseudofurcata*, i.e., specimens of strain CIL-2019/3 from Lake Zurich *in vivo* **(A)**, after protargol staining **(B,C)**, and in the scanning electron microscope **(D)**. **(A–C)** Longitudinal optical sections. **(D)** Oblique posterior polar view. CC, caudal cilia; CV, contractile vacuole; EX, extrusome; MA, macronucleus; OB, oral basket; OF, oral flaps; SC, somatic cilia. Scale bars 10 μm **(A)** and 5 μm **(B–D)**.

*Urotricha pseudofurcata* Krainer, 1995 - Strain CIL-2019/3 (Figures 4A–D and Supplementary Table 4)

Remarks. Out of the two genetically identical strains, CIL-2019/3 and CIL-2019/4, the former was studied morphologically *in vivo* and after protargol staining.

Description. The cell is ellipsoidal to broadly ellipsoidal. It measures 16–24 × 10–19 μm *in vivo* and 9–20 × 6–14 μm in protargol preparations. The cell length:width ratio is 1.2-1.8:1 *in vivo* and in protargol preparations. The unciliated posterior cell portion occupies 15–55%, on average 29% of cell length, being 1–9 μm long in protargol preparations. The globular macronucleus is 3–7 μm across *in vivo* and measures 4–7 × 4–6 μm in protargol preparations; the globular micronucleus is 1–2 μm across *in vivo*, whereas it cannot be distinguished from stained food vacuoles in protargol preparations. The minute rod-shaped extrusomes are invisible *in vivo*, but easily recognizable in protargol preparations, where they are potentially deformed and 0.5–1.0 μm long (Figures 4B,C), and as minute stubs in scanning electron micrographs (Figure 4D). The cytoplasm is colorless and includes refractive lipid droplets 1–3 μm across and often many (colorful) food vacuoles 2–10 μm, on average 5 μm across. The contractile vacuole is up to 6 μm across (Figure 4A). The somatic cilia are relatively long with 3–5 μm *in vivo* and 3–6 μm in protargol preparations. The length of the two caudal cilia is about one third to one half of cell length, namely, 5–9 μm *in vivo* and in protargol preparations (Figures 4A–D). The somatic ciliature comprises 14–24 kineties consisting of 8–14 basal bodies in anteriorly unshortened rows. The probably three adoral organelles (*n* = 1) are hardly visible, especially adoral organelles 2 and 3: adoral organelle 1 consists of 3–5 dikinetids and is 1.5–2.5 μm in length, adoral organelle 2 consists of two or three dikinetids and is about 1–1.5 μm in length (*n* = 7), and adoral organelle 3 probably consists of three dikinetids and is about 1 μm in length (*n* = 1). The 8–12 oral flaps are 1–3 μm long in protargol preparations (Figures 4B,C). The oral basket is 3–5 μm across distally and 4–12 μm long in protargol preparations, occupying 28–75%, on average 45% of cell width (Figure 4C).

**FIGURE 5:**
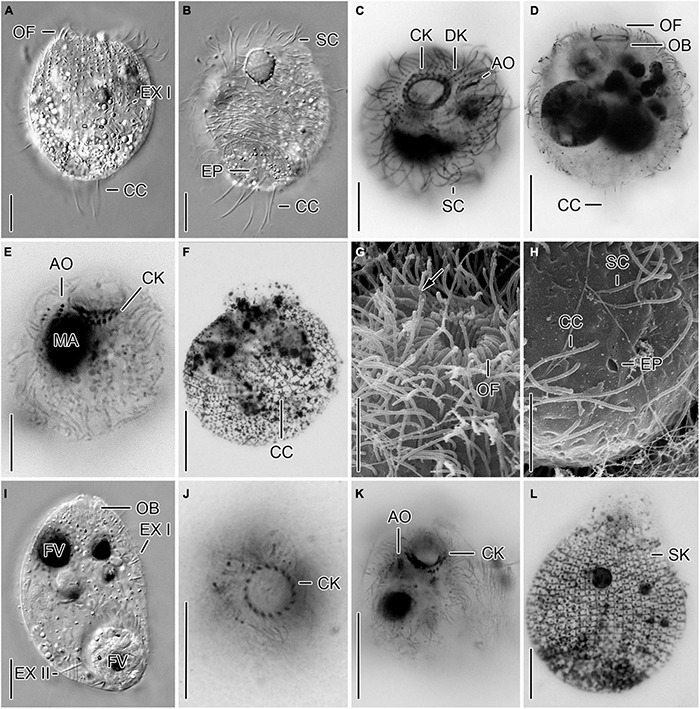
Cryptic species tentatively identified as *Urotricha castalia*. One species **(A–H)** is represented by specimens of strains CIL-2019/2 **(A,B)**, CIL-2019/1 **(C,D,F–H)**, and of the non-clonal original culture **(E)** from Lake Zurich, the other species **(I–L)** by specimens of strains CIL-2017/27 **(I)** and CIL-2017/25 **(J–L)** from Lake Mondsee *in vivo* **(A,B,I)**, after protargol staining **(C–E,J,K)**, after dry silver nitrate staining **(F,L)**, and in the scanning electron microscope **(G,H)**. **(A,E,L)** Lateral views. Occasionally, only the ciliated basal bodies of the adoral organelles stain **(E)**. The silverline pattern is typical **(L)**. **(B)** Ventral view. The excretory pore is within the circle of caudal cilia. **(C,G,K)** Oblique top views. Arrow denotes distinct ridges between adoral organelles. **(D,I)** Longitudinal optical sections. **(F,H)** Oblique posterior polar views. The silverline pattern is composed of polygonal meshes in the unciliated cell portion and the caudal cilia insert in rather circular meshes **(F)**. The excretory pore is within the circle of caudal cilia **(H)**. **(J)** Top view. AO, adoral organelles; CC, caudal cilia; CK, circumoral kinety; DK, dikinetids at anterior end of somatic kineties; EP, excretory pore; EX I, small extrusomes in ciliated anterior cell portion; EX II, large extrusomes in unciliated posterior cell portion; FV, food vacuole; MA, macronucleus; OB, oral basket; OF, oral flaps; SC, somatic cilia; SK, somatic kineties. Scale bars 10 μm **(A–F,I–K)** and 5 μm **(G,H,L)**.

*Urotricha castalia* Muñoz et al., 1987 (Figures 2G,H, 5A–L and Supplementary Table 4).

Remarks. Out of the five genetically analyzed strains, three were morphologically investigated. The genetic data indicate a conspecificity of the strains CIL-2017/25, CIL-2017/26, and CIL-2017/27 from Lake Mondsee, whereas the strains CIL-2019/1 and CIL-2019/2 from Lake Zurich are morphologically similar but genetically distinct from those of Lake Mondsee. Since the two genetic clusters are statistically well-supported and corroborated by CBCs, they probably represent cryptic species whose morphometric data are thus kept separate.

*Urotricha castalia* Muñoz et al., 1987 - Strains CIL-2017/25 and CIL-2017/27 from Lake Mondsee (Figures 5I–L).

Remarks. Of the three genetically identical strains CIL-2017/25, CIL-2017/26, and CIL-2017/27, only the former and latter were morphometrically analyzed *in vivo* and in protargol preparations; strain CIL-2017/25 was also stained following the dry silver nitrate method.

Description. The cell is broadly ellipsoidal. It measures 26–59 × 25–44 μm *in vivo* and 17–29 × 13–23 μm in protargol preparations. The cell length:width ratio is 0.8-1.8:1 *in vivo* and 1.1-1.8:1 in protargol preparations. The unciliated posterior end is slightly set off plug-like and occupies 9–32%, on average 19% of cell length, measuring 2–6 μm in protargol preparations, whereas it is 7–15 μm long in living cells of strain CIL-2017/25. The globular to broadly ellipsoidal macronucleus measures 9–15 × 7–13 μm *in vivo* and is 5–12 μm across in protargol preparations; the globular micronucleus was only observed in strain CIL-2017/27 and has a size of 2–4 × 2–3 μm *in vivo* (*n* = 2) and of 2–3.5 × 1.5–3 in protargol preparations. Two types of rod-shaped somatic extrusomes occur and could only be studied *in vivo*, as they did not stain with protargol: the type I extrusomes are restricted to the ciliated anterior cell portion, where they are rather densely arranged and with a length of 1–3 μm shorter than the type II extrusomes of the unciliated posterior cell portion measuring 3–6 μm (Figure 5I). The cytoplasm is colorless and includes refractive lipid droplets and often many (colorful) food vacuoles 4–7 μm, on average 6 μm across. The contractile vacuole is up to 18 μm across; its excretory pore is encircled by the caudal cilia. The somatic cilia are 2–6 μm long in protargol preparations. The somatic ciliature comprises 32-42 kineties consisting of 13-27 cilia in anteriorly unshortened rows. The length of the 4-7 caudal cilia is about one fourth of the cell length, namely, 7–13 μm *in vivo* and 3–9 μm in protargol preparations. The three adoral organelles are difficult to observe and thus to study; they decrease in size from the anterior to the posterior brosse end; adoral organelle 1 consists of four dikinetids and is 1.2–2.5 μm long, adoral organelle 2 consists of three dikinetids and is 1.2–2.0 μm long, and adoral organelle 3 consists of 1–3 dikinetids and is 0.2–1.2 μm long (Figure 5K). The oral basket is 3–6 μm across distally and 7–18 μm long in protargol preparations, occupying 20–37%, on average 27 and 29% of cell width, respectively. The 15–21 oral flaps are 1.5–2.8 μm long in protargol preparations (Figures 5J,K). The silverline system is typical, i.e., the dry silver nitrate preparation reveals longitudinal rows of rectangular meshes each encompassing a somatic kinetid. Pairs of rows commence with a pentagonal mesh. The meshes are larger and polygonal in the unciliated posterior pole area with the caudal cilia inserting in minute circular meshes (Figure 5L).

*Urotricha castalia* Muñoz et al., 1987 - Strains CIL-2019/1 and CIL-2019/2 from Lake Zurich (Figures 2G,H, 5A–H).

Remarks. Two genetically identical strains were found, i.e., the strains CIL-2019/1 and CIL-2019/2. The former was studied after applying various techniques, i.e., live observation, protargol (QPS and Foissner’s method) and silver nitrate staining as well as scanning electron microscopy. Live specimens of strain CIL-2019/2 were documented (Figures 5A,B).

Description. The cell is broadly ellipsoidal. It measures 25–53 × 26–46 μm *in vivo* and 24–42 × 21–38 μm in protargol preparations. The cell length:width ratio is 0.8-1.4:1 *in vivo* and 1.1-1.4:1 in protargol preparations. The unciliated posterior end is slightly set off plug-like and occupies 11–25%, on average 21% of cell length, measuring 3–9 μm in protargol preparations. The globular macronucleus measures 7–15 × 7–12 μm *in vivo* and 7–14 × 5–13 μm after protargol impregnation; the globular micronucleus was observed in living cells and was 1–5 μm across and only once after protargol impregnation where it measured 3 × 2 μm. Two types of rod-shaped somatic extrusomes occur and could only be studied *in vivo*, as they do not stain with protargol: the type I extrusomes are restricted to the ciliated anterior cell portion, where they are rather densely arranged and with a length of 1–2 μm shorter than the type II extrusomes of the unciliated posterior portion measuring 3–5 μm (Figure 5A). The cytoplasm is colorless and includes refractive lipid droplets 1–3 μm across and often many (colorful) food vacuoles 3–10 μm, on average 6 μm across. The contractile vacuole is up to 17 μm across; its excretory pore is encircled by the caudal cilia as also recognizable in scanning electron micrographs (Figures 5B,H). The somatic cilia are 4–9 μm long *in vivo* and 2–5 μm in protargol preparations. The somatic ciliature comprises 34-46 kineties consisting of 18-30 cilia in anteriorly unshortened rows. Each of the 4-7 caudal cilia inserts in a pit surrounded by an elevated ring and is about one fifth of the cell length, namely, 4–11 μm both *in vivo* and in protargol preparations (Figures 2H, 5A,B,H). The three (*n* = 14) adoral organelles (“brosse”) are difficult to observe and thus to study; they are separated by distinct ridges and decrease in size from the anterior to the posterior brosse end: adoral organelle 1 consists of four or five dikinetids and is 2–3 μm long, adoral organelle 2 consists of three or four dikinetids and is 1–2 μm long, and adoral organelle 3 consists of two or three dikinetids and is 0.5–1.5 μm long (Figures 2G, 5C,E,G). The oral basket is *in vivo* 6–19 μm across distally and 6–23 μm long, occupying 20–60%, on average 40% of cell width, while it is 4–10 μm across and 8–16 μm long in protargol preparations, occupying 20–30%, on average 30% of cell width (Figure 2H). The 19-29, on average 23 oral flaps are 2–4 μm long in protargol preparations (Figures 5A,C–E,G). The silverline system is typical, i.e., the dry silver nitrate preparation reveals longitudinal rows of rectangular meshes each encompassing a somatic kinetid. The meshes are larger and irregular in the unciliated posterior pole area with the caudal cilia inserting in minute circular meshes (Figure 5F).

### Optimal Cultivation Media and Food

Of all media tested, a mixture of WC:Volvic^®^ 5:1 with SAG 26.80 *Cryptomonas* sp. as food resource was most successful for the growth of *U. castalia*; hence, it was also used for all other *Urotricha* strains (Table 2). The medium WC:Volvic^®^ 1:1 was also suitable, but not employed for this study. Overall, the results revealed that *U*. *castalia* was feeding only on the mobile alga SAG 26.80 *Cryptomonas* sp., while not on immobile food items (MS-2017/1 *Coelastrum* sp., MS-2017/2 *Choricystis* sp., MS-2017/8 *Cosmarium* sp., and MS-2017/7 *Acutodesmus obliquus;* Supplementary Figures 1, 3).

### Molecular Phylogeny of the Genus *Urotricha*

**FIGURE 6:**
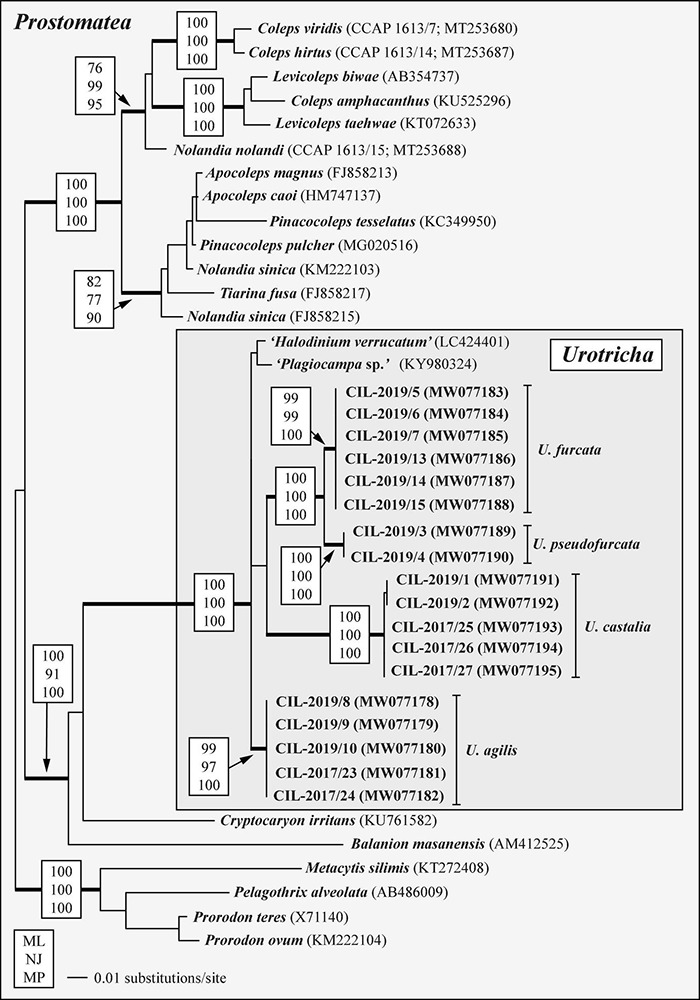
SSU rDNA phylogeny of 35 prostomatean taxa inferred from 1,755 aligned positions of the sequences analyzed by maximum likelihood, neighbor joining, and maximum parsimony methods in PAUP 4.0a167. Best model was GTR + I + Γ model (base frequencies: A 0.2777, C 0.1830, G 0.2486, U 0.2906; rate matrix: A-C 1.3273, A-G 2.7354, A-U 1.5527, C-G 0.2974, C-U 4.7063, G-U 1.0000) with the proportion of invariable sites (*I* = 0.6584) and gamma distribution shape parameter (Γ = 0.6289) as calculated with the automated model selection tool implemented in PAUP. The branches in bold are highly supported in all bootstrap analyses (bootstrap values > 50% calculated with PAUP, using the maximum likelihood, neighbor joining, and maximum parsimony). The GenBank accession numbers in brackets follow the species names. The accession numbers in bold indicate strains investigated in this study.

Phylogenetic analyses of SSU rDNA sequences revealed that all investigated strains of *Urotricha* formed a highly supported monophyletic group together with *Plagiocampa* sp. (KY980324; Zhang et al., 2014) and *Halodinium verrucatum* (LC424401; Gurdebeke et al., 2018), which is placed in a distinct clade. Hence, the identification of the latter two sequences is incorrect or the genus *Urotricha* is not monophyletic. The sister group of *Urotricha* were the Colepidae including the genus *Coleps* (Figure 6).

**FIGURE 7:**
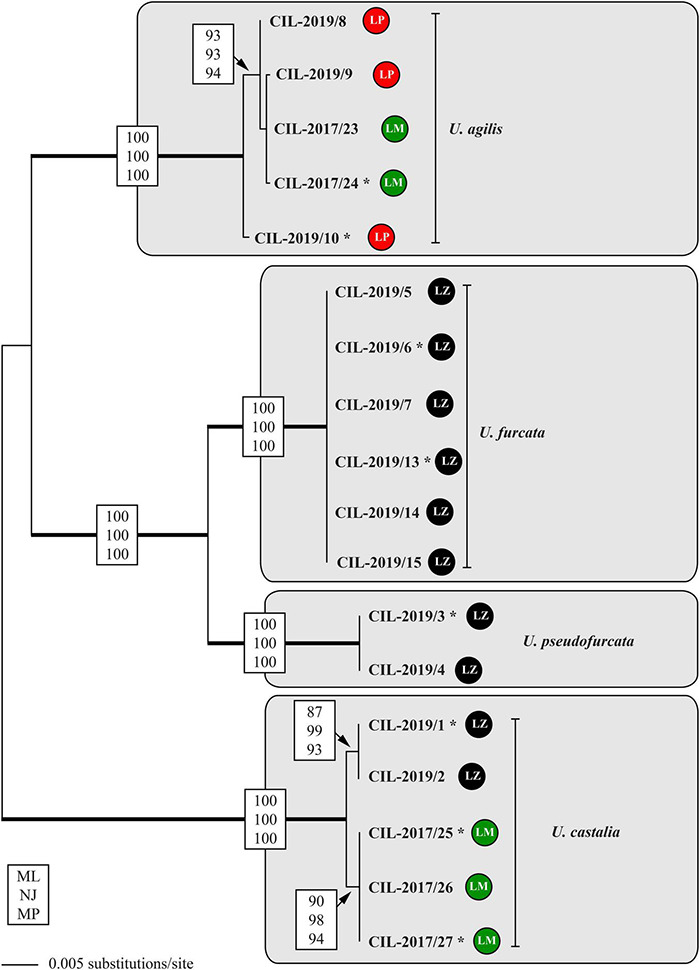
Molecular phylogeny of the genus *Urotricha* inferred from SSU and ITS rDNA sequences, using the maximum likelihood method of PAUP 4.0a167 on a dataset with 2,343 aligned positions of 18 taxa. For the analyses, the best model was calculated with automated model selection tool implemented in PAUP. The setting of the best model was given as follows: GTR + I (base frequencies: A 0.3017, C 0.1700, G 0.2153, T 0.3130; rate matrix A-C 1.3248, A-G 1.4840, A-U 1.8283, C-G 0.5727, C-U 2.8434, G-U 1.0000) with the proportion of invariable sites (*I* = 0.7703). The branches in bold are highly supported in all bootstrap analyses (bootstrap values > 50% calculated with PAUP, using the maximum likelihood, neighbor joining, and maximum parsimony). The circles following the strain designation indicate the strain origins (LP = Lake Piburg, Austria; LM = Lake Mondsee, Austria; LZ = Lake Zurich, Switzerland). Strains labeled with an asterisk were morphologically investigated.

The analyses of the concatenated dataset comprising SSU rDNA and ITS sequences provided a better resolution among the *Urotricha* strains than the analyses of the SSU rDNA dataset alone. The genus *Urotricha* formed four highly supported lineages, i.e., *U. agilis*, *U. furcata*, *U. pseudofurcata*, and *U. castalia* in all bootstrap analyses (Figure 7). The strains from Lake Mondsee tentatively identified as *U. castalia* differed slightly in their SSU rDNA and ITS sequences from the strains isolated from Lake Zurich; both form two well-supported clades, probably representing cryptic species.

The tree topology regarding the strains tentatively identified as *U. agilis* was incongruent with the geography, displaying a grouping of two sequences from Lake Mondsee with two from Lake Piburg. This cluster represented again a statistically supported adelphotaxon to another sequence from Lake Piburg. Hence, at least two genetically distinct strains of *U. agilis* occurred in Lake Piburg (Figure 7). All strains of *U. furcata* and those tentatively identified as *U. pseudofurcata* came from Lake Zurich; they form highly statistically supported adelphotaxa; within each clade, the sequences were identical.

**FIGURE 8:**
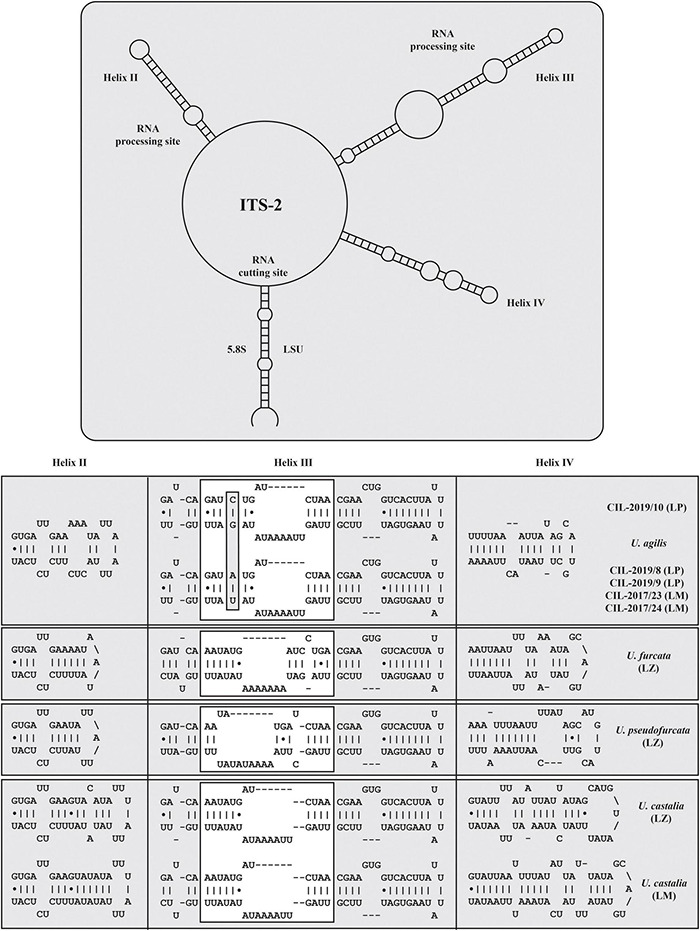
Internal transcribed spacer-2 secondary structures of the investigated *Urotricha* strains. The variable regions are marked by white boxes. The compensatory base changes are highlighted. The structures were calculated with mfold. The line graphic was drawn with PseudoViewer. LP, Lake Piburg population; LM, Lake Mondsee population; LZ, Lake Zurich population.

**FIGURE 9:**
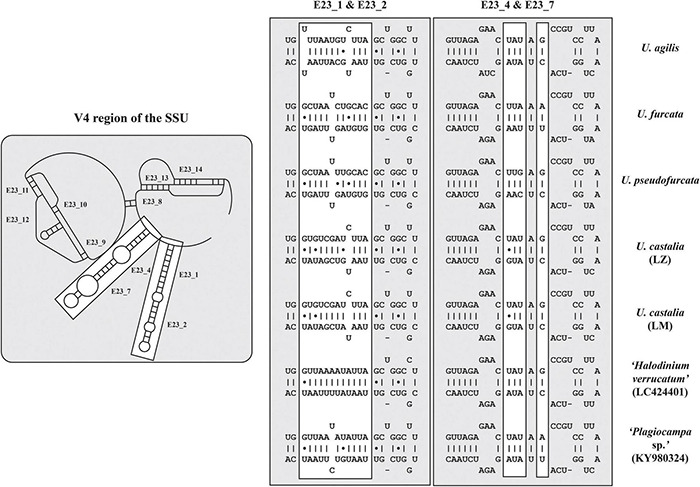
V4 secondary structures of the investigated *Urotricha* strains. The variable regions are marked by white boxes. The structures of E23_1/E23_2 and E23_4/E23_7 were calculated with mfold. The line graphic was drawn with PseudoViewer. LM, Lake Mondsee population; LZ, Lake Zurich population.

The secondary structures of the ITS-1 and ITS-2 sequences were analyzed for detecting compensatory base changes (CBCs) in the four species. Both ITS regions of the urotrichs studied here consisted of only three helices (ITS-1: Helix 1-3; ITS-2: Helix II-IV) as Helix 4 of the ITS-1 and Helix I of the ITS-2 are missing. Using the ITS-2/CBC approach, all species had specific loops and were supported by CBCs and hemi-CBCs (one-sided base changes; HCBCs) (Figure 8 and Table 3). Not only the phylogeny of the concatenated sequence data, but also the detection of CBCs suggested genetically distinct clades within the specimens tentatively identified as *U. agilis* and *U. castalia.* Unfortunately, no ITS sequences of *Urotricha* species beyond those of the present study were available in GenBank. Therefore, we analyzed the variable regions of the SSU, V4, and V9, which were used in high throughput sequencing approaches for the discovery of species distribution. The V9 cannot always discriminate the different *Urotricha* species, i.e., the strains identified as *U. furcata* and tentatively as *U. pseudofurcata* had identical V9 sequences (Supplementary Figure 4). However, the V4 region appeared promising for species-delimitation of the *Urotricha* strains tested and can be considered as a diagnostic barcode region (Figure 9).

**TABLE 3:** Compensatory base changes and hemi-compensatory base changes among the ITS-2 rDNA sequences of the investigated *Urotricha* strains.

				1	2	3	4	5	6	7	8	9	10	11	12	13	14	15	16	17	18
*U. agilis*	CIL-2019/8	1	LP		0	1	0	0	1	1	1	1	1	1	0	0	1	1	0	0	0
	CIL-2019/9	2	LP	0		0	0	0	2	2	2	2	2	2	0	0	0	0	0	0	0
	CIL-2019/10	3	LP	1	1		1	1	1	1	1	1	1	1	1	1	0	0	0	0	0
	CIL-2017/23	4	LM	0	0	1		0	2	2	2	2	2	2	0	0	1	1	0	0	0
	CIL-2017/24	5	LM	0	0	1	0		2	2	2	2	2	2	0	0	1	1	0	0	0
*U. furcata*	CIL-2019/5	6	LZ	2	2	3	2	2		0	0	0	0	0	1	1	2	2	2	2	2
	CIL-2019/6	7	LZ	2	2	3	2	2	0		0	0	0	0	1	1	2	2	2	2	2
	CIL-2019/7	8	LZ	2	2	3	2	2	0	0		0	0	0	1	1	2	2	2	2	2
	CIL-2019/13	9	LZ	2	2	3	2	2	0	0	0		0	0	1	1	2	2	2	2	2
	CIL-2019/14	10	LZ	2	2	3	2	2	0	0	0	0		0	1	1	2	2	2	2	2
	CIL-2019/15	11	LZ	2	2	3	2	2	0	0	0	0	0		1	1	2	2	2	2	2
*U. pseudofurcata*	CIL-2019/3	12	LZ	0	0	1	0	0	2	2	2	2	2	2		0	1	1	2	2	2
	CIL-2019/4	13	LZ	0	0	1	0	0	2	2	2	2	2	2	0		1	1	2	2	2
*U. castalia*	CIL-2019/1	14	LZ	1	1	3	1	1	3	3	3	3	3	3	0	0		0	0	0	0
	CIL-2019/2	15	LZ	1	1	3	1	1	3	3	3	3	3	3	0	0	0		0	0	0
	CIL-2017/25	16	LM	1	1	3	1	1	2	2	2	2	2	2	1	1	1	1		0	0
	CIL-2017/26	17	LM	1	1	3	1	1	2	2	2	2	2	2	1	1	1	1	0		0
	CIL-2017/27	18	LM	1	1	3	1	1	2	2	2	2	2	2	1	1	1	1	0	0	

*The bottom left corner shows the HCBCs, the upper right the CBCs of ITS-2. LP, Lake Piburg, Austria; LM, Lake Mondsee, Austria; LZ, Lake Zurich, Switzerland.*

### Global and Local Distribution of Urotrichs

**FIGURE 10:**
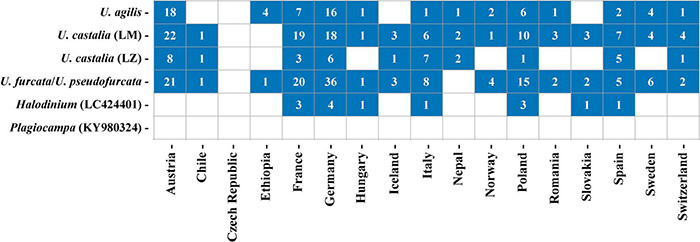
Distribution of the investigated *Urotricha* strains. Blue squares indicate the occurrence of V4- or V9-markers in a country and the numbers give the number of lakes in which the V4- or V9-markers were found per country. Detailed information about the investigated freshwater lakes can be retrieved from Supplementary Table 2. Since *U. furcata* and *U. pseudofurcata* show a 100% sequence similarity in their V9-region, only results for *U. furcata* are shown.

In total, 252 freshwater lakes in 17 different countries were screened for the presence of urotrich V4 or V9 barcode markers (Figure 10 and Supplementary Tables 2, 3). The barcode markers occurred in all investigated countries, except for the Czech Republic, in which, however, only a single lake was sampled. The strains from Lake Mondsee tentatively identified as *U. castalia* and the strains of *U. furcata/U. pseudofurcata* were found in 15 different countries and in most lakes, making them globally and regionally the most widespread strains. The strain from Lake Zurich again tentatively identified as *U. castalia* showed a similar global distribution as the sister clade, but was less common. The strains tentatively identified as *U. agilis* could also be found worldwide with a high occurrence in Austria and Germany (Figure 10). In contrast, LC424401_*Halodinium* appeared to be restricted to Europe, being present in only seven out of 17 countries.

Using Lake Mondsee and Lake Zurich as case studies, the spatial and temporal distribution of *Urotricha* species was studied in greater detail. For this purpose, the cell count data obtained from morphological investigations and the HTS data were compared. Overall, the morphology-based data and HTS data seem to be more divergent for Lake Mondsee than for Lake Zurich (Supplementary Figures 5, 6). In Lake Mondsee, both datasets for *U. agilis* revealed a broad spatial distribution ranging from 0 to 40 m depth. Differences, however, occurred on a temporal scale, as, based on the cell count data, *U. agilis* was mainly present in June and in the winter months, whereas the HTS data showed a presence throughout the year, except for the winter months. A more congruent picture is obtained from the *U. castalia* datasets. Both indicated a broad spatial distribution with high abundances in the upper water layers (0–12 m depth) and a confined seasonal occurrence from June to November/December. Since *U. furcata/U. pseudofurcata* could not be differentiated based on their V9 region (Supplementary Figure 4), the cell count data of *U. furcata* and *U. pseudofurcata* were compared against the single HTS dataset. The latter indicate the presence of *U. furcata/U. pseudofurcata* over all depths and the year. The cell count data, however, revealed morphotype-specific differences, as *U. furcata* preferred upper water layers (0–12 m depth) and warmer months (May to September), while *U. pseudofurcata* was distributed throughout the entire water body, preferably in the winter-spring months (November to May).

In Lake Zurich, the abundance of *U. agilis* demonstrated a more restricted spatial distribution than the HTS data (20 m and 40 m depth, respectively, Supplementary Figure 6), however, apart from the coinciding temporal distribution in both approaches from April to November, cell counts revealed individuals occurring also between June and October, respectively. Equally congruent are the data for *U. castalia*, which revealed an occurrence from 0 to 20 m depth and, in low abundance, also at 120 m depth, however, with a temporal restriction to the warmer months (April to October). The greatest deviations were observed for the cell count and HTS data of *U. furcata/U. pseudofurcata*. Whereas *U. pseudofurcata* showed its presence in 0–40 m water depths and an absence over the winter months, i.e., December to March, the HTS data for *U. furcata/U. pseudofurcata* indicated a distribution all over the entire water column restricted to June through October.

### Effects of Environmental Factors on Urotrichs

Statistical analyses were conducted to reveal whether urotrich ciliate abundances (read numbers) were a function of environmental variables and altitude. In general, hardly any significant correlations between urotrich ciliate abundance and environmental factors could be detected. Only the strains of *U. castalia* from Lake Mondsee correlated, albeit weakly, negatively with K^+^ (cor = –0.22, *p* = 0.04), and *U. furcata/U. pseudofurcata* also only weakly negatively with altitude (cor = – 0.18, *p* = 0.01) and weakly positive with SO_4_^2–^ (cor = 0.23, *p* = 0.03) (Supplementary Figure 7).

A multiple logistic regression analysis was used to model the effect of a set of environmental factors on the abundance of individual urotrich species. The fitted regression models are summarized in Table 4 and Supplementary Table 5. Significant positive correlations were only found for two out of the five tested species. The final model for *U. agilis* included Na^2+^, DOC, conductivity, and SO_4_^2–^ (*p* = 0.005) and the final model for *U. furcata*/*U. pseudofurcata* DP, H^+^, DOC, Ca^2+^, altitude, DRSi, and TP (*p* < 0.001).

**TABLE 4:** Best models of multiple linear regression analyses to explain the relationship between environmental parameters and *Urotricha* abundance data.

Species	Best model	Multiple R-squared	Adj. R-Squared	*p*-Value
*U. agilis*	Na + DOC + conductivity + SO4	0.218	0.179	0.0005
*U. castalia* (LM)	−	−	−	−
*U. castalia* (LZ)	−	−	−	−
*U. furcata/U. pseudofurcata*	DP + [H] + DOC + Ca + altitude + DRSi + TP	0.368	0.311	0.0059
*Halodinium* (LC424401)	−	−	−	−

*LM, Lake Mondsee; LZ, Lake Zurich; DOC, dissolved organic carbon; DP, dissolved phosphorus; DRSi, dissolved reactive silica; TP, total phosphorus.*

## Discussion

### Comparison of the Investigated *Urotricha* Strains With Congeners

*Urotricha agilis* (Stokes, 1886) Kahl, 1930. In the original description, Stokes (1886) mentioned and illustrated a conical cell shape, a single caudal cilium of roughly cell length, kineties occupying the anterior two thirds of the about 14 μm long cell, an eccentric contractile vacuole in the posterior cell portion, and a globular and eccentrically located macronucleus near mid-body. The unique conical cell shape was used by Penard (1922) and Kahl (1930) for identifying the species, which has been redescribed from life and after silver staining by Foissner (1979); further, it had been included in two taxonomic and ecological monographs on freshwater ciliates (Foissner et al., 1994, 1999). Again, the conical shape of the 10–20 μm long ciliate was mentioned as the main distinguishing feature. According to the redescription, the species has 12-14 somatic kineties and about seven cilia/kinetids per kinety (as inferred from the illustrations). Data on the adoral organelles and the circumoral kinety are not available.

In the present study, five strains were genetically analyzed and four of them form a statistically well-supported and by CBCs corroborated clade being sister to strain CIL-2019/10. The latter strain and CIL-2017/24 were also morphometrically studied. Both are highly similar, merely differing in the average number of somatic kineties (14-22, on average 18 vs. 18-24, on average 22) and cilia in an anteriorly unshortened kinety (5-7, on average 6 vs. 6-12, on average 9) as well as in the length of the unciliated posterior cell portion occupying 26–48%, on average 37% vs. 17–37%, on average 26% of cell length. The three tiny adoral organelles were recognizable in few specimens *in vivo* and after protargol staining; however, the number of dikinetids per organelle slightly varied among the strains CIL-2017/24 and CIL-2019/10 (three to five vs. four or five, both on average four in adoral organelle 1, two or three vs. two, both on average two in adoral organelle 2, one or two vs. two, both on average two in adoral organelle 3) (**Figures 1B,C**, 2A).

Our strains match *U. agilis* in cell size (*in vivo* 14–23 μm and 13–19 μm vs. 10–20 μm; Foissner, 1979) and the blank stripe separating the somatic kineties from the circumoral kinety, not present in other congeners of this size class with one caudal cilium (Figures 1C,D). However, the strains are not conical (Figures 1A,B,E–G) and have more somatic kineties (18-24, on average 22 and 14-22, on average 18 vs. 12-14). Strain CIL-2017/24 might also differ by a higher number of kinetids in an unshortened kinety (6-12, on average 9 vs. about 7). Despite these differences, we tentatively assign our strains as two cryptic species to the *Urotricha agilis*-complex. A far-reaching revision requires the analyses of further marker sequences, particularly, of *U. agilis* collected at the type locality or in its surroundings in the United States, which is beyond the focus of this study.

Two species have been considered being synonyms of *U. agilis*, i.e., *Urotricha gyrans* Stokes, 1888 (Foissner, 1979) and *Urotricha nais* Muñoz et al., 1987 (Foissner et al., 1999). Foissner (1979) attributed the slightly different (ellipsoidal) shape of *U. gyrans* to a slight squeezing of the cell by the cover glass pressure. Additionally, Foissner et al. (1999) proposed *U. nais* Muñoz et al., 1987 as a junior synonym of *U. agilis*; yet, both species differ in the numbers of kineties (12-14 vs. 18-21 in *U. nais*). Since *U. nais* also has minute extrusomes, a distinct blank stripe between the somatic kineties and the circumoral kinety, an unciliated posterior portion that is not set off plug-like, and 18-21 somatic kineties, strain CIL-2017/24 and the genetically similar strains CIL-2017/23, CIL-2019/9, and CIL-2019/8 might be conspecific.

*Urotricha furcata* Schewiakoff, 1892. Three freshwater urotrichs with two caudal cilia are known: *Urotricha macrostoma* Foissner, 1983 (cell length *in vivo* 30-40 μm, three adoral membranelles, and minute extrusomes); *U. pseudofurcata* Krainer, 1995 (distinct extrusomes); and *U. furcata* Schewiakoff, 1892 (no extrusomes).

The latter species, originally described from Hawaii by Schewiakoff (1892), has authoritatively been redescribed from life and after silver staining by Foissner and O’Donoghue (1990) from Australia and included in two taxonomic and ecological monographs on freshwater ciliates (Foissner et al., 1994, 1999). Since the Australian specimens matched the original description and the later illustrations by Schewiakoff (1892, 1893), Foissner and O’Donoghue (1990) merely described the stained cell and provided morphometrics. *Urotricha furcata* matches our genetically identical strains in the apparent lack of extrusomes, the cell size *in vivo* (20–30 × 15–20 μm vs. 17–35 × 10–29 μm; Foissner et al., 1999) and after protargol staining (14–20 × 11–15 μm vs. 11–19 × 10–16 μm; Foissner and O’Donoghue, 1990), the cell shape, the number of somatic kineties (22–24 vs. 18–24) and kinetids in unshortended rows (13–21 vs. 10–14), however, not in the number of adoral organelles (two vs. three; Foissner and O’Donoghue, 1990). Despite the latter difference, the strains CIL-2019/6 and CIL-2019/13 are identified as *U. furcata*.

*Urotricha pseudofurcata* Krainer, 1995. As mentioned above, there are three urotrichs with two caudal cilia: *Urotricha macrostoma* Foissner, 1983 (minute extrusomes); *U. furcata* Schewiakoff, 1892 (no extrusomes); and *U. pseudofurcata* Krainer, 1995 (distinct extrusomes). Our strain deviates from *U. macrostoma* in the cell length (*in vivo* 16–24 μm vs. 30–40 μm) and the diameter of the oral opening (4 μm vs. 8 μm; Foissner, 1983) and from *U. furcata* in the presence of extrusomes (vs. absent; see above).

The species description of *U. pseudofurcata* by Krainer (1995) is based on live observations and protargol-stained material from Austria. The strain CIL-2019/3 is most similar to *U. pseudofurcata*, but differs in the number of somatic kineties (15–24 vs. 25–27; Krainer, 1995). Due to the different preparation methods, the length of the rod-shaped extrusomes (0.5–1.5 μm in protargol preparations vs. 3–4 μm *in vivo*; Figures 4B–D) is hardly comparable. Although our strain thus probably represents a distinct species, it is tentatively assigned to the *U. pseudofurcata*-complex until all taxonomically relevant data on our strain and the marker gene sequences for *U. pseudofurcata* collected at the type locality are available.

*Urotricha castalia* Muñoz et al., 1987 (Table 5). The original description by Muñoz et al. (1987) is based on silver carbonate-stained and probably squashed and swollen specimens collected in an artificial pond in the “Parque de Berlin,” Madrid, Spain; their size and shape are thus hardly comparable with specimens prepared with different methods. Later, Foissner and Pfister (1997) investigated supposedly conspecific specimens from an Austrian and a German population and synonymized *U. rotunda* Fernandéz-Leborans and Novillo, 1994 with *U. castalia*.

**TABLE 5:** Comparison of main characters in the *Urotricha castalia*-complex based on stained material (average values given in brackets).

Character	*U. castalia*					Suggested synonym *U. rotunda*
	Type population	Salzburg population	Lake Constance	Lake Zurich	Lake Mondsee	
Somatic kineties, number	47-50	35-46(40)	34-41(38)	34-40(39)	32-42(36)	45-48(46)
Cilia in a somatic kinety, number	16-18	20-28(24)	14-28(20)	18-30(21)	13-27(18)	30-36(33)
Caudal cilia, number	5-7	8-10(9)	4-9(7)	4-7(5)	4-7(5/6)	6-8(7)
Circumoral dikinetids, number	20-25	16-25(21)	17-24(19)	19-29(23)	15-21(18)	22-24(23)
Adoral organelles, number	3	3-5(3)	3	3	3	3
Adoral organelle 1, dikinetid number	5-9	4 or 5(4)	4 or 5(5)	4 or 5(5)	4 (4)	7-10(8)
Adoral organelle 2, dikinetid number	4-6	3-5(4)	3 or 4(4)	3 or 4(3)	3 (3)	6 or 7(6)
Adoral organelle 3, dikinetid number	3-5	2 or 3(2)	2 or 3(3)	2 or 3(2)	1-3(2)	3 or 4(3)
Type I extrusomes, length Type II extrusomes, length	abundant, but diff. types not mentioned	2.5-3 5-6	similar to Salzburg population	1.3-2.2 3-5.3	0.8-2.9 3.4-6	not mentioned not mentioned
Reference	Muñoz et al. (1987)	Foissner and Pfister (1997)		This study		Fernandéz-Leborans and Novillo (1994)

The results of our study obtained from the genetic data, particularly the CBCs, suggest two species in our material, i.e., one species that we found exclusively in Lake Zurich and the other one in Lake Mondsee (Figure 8). They are morphologically very similar, merely differing in the average number of circumoral dikinetids (19-29, on average 23 vs. 15-21, on average 17 and 18, respectively).

Our strains morphologically match the specimens from Austria (Salzburg) and Germany (Lake Constance), especially in the possession of two different types of extrusomes (Figure 5I), but deviate slightly in the number of caudal cilia (4-7, on average 5 in our strains vs. 8-10, on average 9 in the Salzburg population and 4-9, on average 7 in the Lake Constance population; Figures 5A,B,H; Foissner and Pfister, 1997). In the light of the present genetic data, the minute morphological discrepancies might indicate further cryptic species; however, DNA barcodes are not available for the specimens from Salzburg and Lake Constance. Likewise, data on marker sequences and extrusomes are missing for the Spanish type population (Muñoz et al., 1987) and the suggested synonym, *U. rotunda* (Fernandéz-Leborans and Novillo, 1994). These two populations have more somatic kineties than the previously mentioned ones (Table 5). This difference and another discrepancy, i.e., in the number of cilia in a somatic kinety (16-18 vs. 30-36) in the type population and in *U. rotunda* suggest another two species in the *U. castalia*-complex. Eventually, future morphological and genetic studies are required to verify that the minute deviations in the ciliature observed should not be attributed to intraspecific variability, but rather indicate different species. At the current state of knowledge, these species share 4-10 caudal cilia arranged in a circle, usually three adoral organelles, and an ellipsoidal to broadly ellipsoidal cell shape.

### Molecular Markers and Morphology - Indispensable Requirements for Ciliate Identification

Ciliates form a statistically well-supported and specious mono-phylum (Lynn, 2008; Gao et al., 2016, 2017; Adl et al., 2019). Their high diversity, heterogeneous morphology, biogeographic distribution patterns, and phylogenetic relationships are put more and more into a comprehensive perspective. Ciliate (re-) descriptions and taxonomic assignments should be based on thorough studies of the species-specific diagnostic features, the relevant literature, and biogeographic aspects (Agatha et al., 2021). Furthermore, microscopic and molecular approaches need to be linked, which is still a major challenge in ciliate ecology (Warren et al., 2017).

Identification is difficult, particularly in ciliates less than 50 μm in length and in complexes of cryptic species, e.g., those of the genera *Paramecium* and *Coleps* (Sonneborn, 1975; Krenek et al., 2015; Pröschold et al., 2021). In such cases, molecular markers with an appropriate phylogenetic resolution (V4 and V9 of the SSU rDNA, ITS, D1-D2 of the LSU, mitochondrial COI) and their secondary structure might provide clues for species delimitations (Coleman, 2005; Dunthorn et al., 2012; Stoeck et al., 2014a; Shazib et al., 2016; Pitsch et al., 2019; Pröschold et al., 2021). Molecular barcode markers are needed to place a taxon into ecological guilds as closely related taxa might differ distinctly in their ecological functions (Foissner et al., 2003). This becomes crucial when the ecological processes are inferred from HTS samples (Pawlowski et al., 2014; Stoeck et al., 2014b; de Vargas et al., 2015; Massana et al., 2015; Gimmler et al., 2016; Piredda et al., 2018; Grattepanche and Katz, 2020; Qu et al., 2021). The correct taxonomic annotation of OTUs (Operational Taxonomic Units) or ASVs (Amplicon Sequence Variants) relies on publicly accessible databases; the EukRef platform is a curated database, while others, e.g., NCBI, still tend to be incomplete for ciliate species and the data are not curated (Boscaro et al., 2018; del Campo et al., 2018).

The present study combines morphological, ecological, and genetic approaches and offers a valuable basis for future ciliate research (Spanner et al., 2020; Forster et al., 2021; Pröschold et al., 2021; Qu et al., 2021). It provides the first single cell barcodes of morphologically investigated urotrich ciliates. The findings confirmed the resolution of the SSU rDNA gene at the generic level (Figures 6, 7). The hypervariable V4 region of the SSU rDNA had a higher resolution, allowing even the discrimination of the various *Urotricha* strains, whereas the V9 failed in distinguishing the morphospecies assigned to *U. furcata* and *U. pseudofurcata*, respectively (Figure 9 and Supplementary Figure 4). Interestingly, the closely related *Coleps* group could be separated by means of the V9 sequences, while not using the V4, and the phenotypic plasticity among the 19 clones analyzed was considerable (Pröschold et al., 2021). In the SSU rDNA tree of the prostomatids, two *Urotricha*-related sequences differ morphologically, i.e., *Plagiocampa* sp. (KY980324; Zhang et al., 2014) and *H. verrucatum* (LC424401; Gurdebeke et al., 2018). They probably represent misidentifications or cross-contaminations, especially as both the phylogenetic placement and the signatures of the V4 and the V9 gene regions indicate that they are urotrichs (Figures 6, 9). However, a general genetic marker gene region for ciliates requires more taxon-related studies in the future.

Morphology-based species identification of urotrichs alone is difficult, particularly in the small congeners. Apart from their small cell size, their rapid locomotion hampers live observations, especially at high magnifications; thus, silver-staining methods are indispensable but require some experience to be successfully applied (Foissner and Pfister, 1997; Foissner, 2014). These issues might be responsible for the low number of species descriptions since the monograph on the taxonomy and ecology of planktonic freshwater ciliates by Foissner et al. (1999), i.e., only *Urotricha spetai* and *Urotricha psenneri* have been established (Sonntag and Foissner, 2004; Foissner, 2012). Moreover, genetic datasets are missing or could not be correctly assigned. The present data, especially the genetic clues, propose the existence of small (pseudo-cryptic) urotrichs in freshwater habitats, which also await their description (Sonntag et al., unpublished).

### Molecular Ecology and Biogeography

Recently, DNA metabarcoding studies in oceans and lakes have opened up new perspectives on the high diversity and geographic distribution of protists and ciliates in particular (de Vargas et al., 2015; Gimmler et al., 2016; Qu et al., 2021). Such studies are commonly dedicated to the detection of diversity and its correlation with environmental parameters but rarely focus on individual taxa. However, ciliate abundance can hardly be estimated in environmental barcoding approaches based on OTU or ASV counts, because the number of SSU rDNA gene copies varies considerably within and among species (Zhu et al., 2005; Stoeck et al., 2014b). Therefore, morphospecies counts and OTU abundance rarely coincide (Pitsch et al., 2019; Pröschold et al., 2021). However, this aspect has previously been exhaustively discussed (e.g., Stoeck et al., 2014b; Stern et al., 2018). Nevertheless, the comparison of the taxon-specific spatial and temporal distribution patterns inferred from both HTS datasets and morphological surveys (e.g., Pröschold et al., 2021 and Supplementary Figures 5, 6) might provide hints for niche separation and speciation in cryptic species.

Linking taxa/species with more or less known ecological preferences to DNA metabarcoding datasets is essential for inferring food web structures and processes from HTS datasets (e.g., Forster et al., 2021; Pröschold et al., 2021; Qu et al., 2021). Therefore, we performed an experiment for at least one of our barcoded urotrichs, i.e., strain CIL-2017/25 to gain insights into the ciliate’s autecology. These findings can then be implemented into analyses of the spatial and temporal distribution of the population in natural communities. Accordingly, key players in aquatic food webs can be identified and future research questions and experimental approaches on climate change scenarios, for example, directly addressed. Moreover, we aim tagging OTUs with a name and fill up public databases with sequences to identify and record their specific distribution patterns. In environmental studies, individual taxa can serve as indicators for water or soil quality (e.g., Foissner et al., 1999; Stoeck et al., 2018; Dias et al., 2021). Actually, only some ciliates can exclusively be related to specific environmental conditions, namely, the obligate anaerobic Cariacotrichea or the Armophorea (Lynn, 2004; Orsi et al., 2012).

Autecological studies further form the basis for generating knowledge on the functional ecology and diversity by elucidating a protists’ individual placement in an aquatic or soil food web (e.g., Geisen, 2016; Weisse et al., 2016; Qu et al., 2021). In contrast to the marine environment, for many freshwater ciliates at least some ecological information on food items and preferred environmental conditions (temperature, pH, oxygen concentration etc.) is available (see compilation on the autecology of planktonic species in Foissner et al., 1999 and references therein). For example, the *U. castalia*-complex has previously been tested for its food and temperature response and cultures were kept in modified WC medium (Guillard and Lorenzen, 1972; Weisse et al., 2001). Obviously, members of the *U. castalia*-complex grow well on cryptomonad food. However, we assumed that the ciliate preferred motile prey that triggered a kind of hunting behavior; though, this has not been directly tested here. This finding supports the results of Qu et al. (2021) who suggested diverse flagellates as potential food source for two *U. castalia* populations. Apart from studying morphological and molecular characters for species description as emphasized by Warren et al. (2017), we strongly encourage researchers to also include autecological datasets to experimentally address specific research questions on predator-prey relationships or food preferences.

Despite some anecdotal notes on food items and environmental conditions, the successful collection of the desired species in the field and its cultivation under laboratory conditions prior to an experiment are problematic. Particularly, the development of cultivation media with appropriate food items for establishing long-term cultures is tricky. Consequently, only those ciliates that are relatively easily kept under laboratory conditions, such as *Paramecium*, *Tetrahymena*, or *Euplotes* species, are intensively studied. Yet, long-term cultures of planktonic species are more difficult to manage and we aim depositing our strains in public culture collections for future research.

### Size Classes

Classifying planktonic organisms, particularly ciliates, according to their sizes was a common procedure in lake ecology in the 20^th^ century, owing to methodological difficulties caused by the application of certain fixatives (e.g., Lugol’s solution) that obscure the taxonomically relevant features. Admittedly, live observations by means of interference contrast optics and the staining methods compiled by Foissner (1991, 2014) are time-consuming and necessitate some experience. Occasionally, scanning electron microscopy might be useful for displaying surface structures, e.g., the adoral organelles and oral flaps in urotrichs. Yet, in modern taxonomy (species identification and description), these methods are indispensable because they distinctly increase the taxonomic resolution and reliability. The quantitative protargol stain (QPS) does not only reveal species-specific cytologic characters (e.g., ciliary pattern and nuclear apparatus) but allows for estimating taxon abundance (Skibbe, 1994). The application of the QPS therefore provides a useful tool for ecological plankton studies (e.g., Pfister et al., 2002; Sonntag et al., 2006, 2011, 2017; Van Wichelen et al., 2013; Kammerlander et al., 2016).

While the usage of ciliate size classes is frequently misleading in ecological studies, urotrichs represent an exception as their two classes obtain somewhat different placements in the food web. Species more than 70 μm long not only feed on picoplankton and nanoplankton as almost all urotrichs, but are also predators of rotifers (see compilation of species and their food preferences in Foissner et al., 1999). Within the size classes, urotrichs might show species-specific food preferences, necessitating the identification to species level. The about 20 μm small urotrich species have one or two caudal cilia (Foissner et al., 1999; Sonntag and Foissner, 2004), whereas the larger urotrichs have three or more caudal cilia. Additional distinguishing features are the presence (and types) of extrusomes and the ciliary pattern.

The example of the urotrichs, demonstrates the need of solid knowledge on the autecology of the taxa under consideration for deciding whether a size-dependent approach for inferring the food web position is justified. Particularly, in genera comprising mixotrophic and heterotrophic species (e.g., in *Coleps*, *Vorticella*, and *Monodinium*), an identification down to species level is indispensable for estimating carbon flux in the habitat.

## Conclusion

In future ecological surveys, DNA metabarcoding approaches will be central tools for elucidating ciliate or protist diversity. We here provide a toolbox on how to investigate ciliate species regarding their morphology, molecular marker sequences, and cultivation conditions as well as their spatial and temporal distribution and biogeography. Although HTS approaches may identify the high diversity of protist species in a certain environment, the phylogenetic placement of a sequence alone is insufficient for inferring the ecological function of a taxon; this information is still only accessible by extensive live observations and/or feeding experiments. The identification of key players or indicator species reflecting alterations in the environmental conditions, e.g., climate change, by their presence/absence or by their abundance is of utmost importance for the future.

The classical taxonomic methods are unable to discriminate cryptic species, which might populate all habitats in considerable numbers. These morphologically identical or highly similar taxa with their specific environmental requirements are detected by analyses of appropriate marker gene sequences. Therefore, we recommend an integrative approach combining morphologic, genetic, and ecological techniques to unravel this unknown diversity.

## Data Availability Statement

The datasets presented in this study can be found in online repositories. The names of the repository/repositories and accession number(s) can be found in the article/Supplementary Material.

## Author Contributions

BS and TP conceived and designed the study. BS, SA, TP, and DF wrote the main manuscript. DF, BS, BK, GD-P, and LN collected most of the strains and performed the morphometric (live observation, protargol staining) and molecular analyses. KQ carried out the experiments with *U. castalia*. TD isolated and cultivated the algal strains. US cultivated the ciliate strains. BW, SA, DF, and BS performed and analyzed the electron microscopic data. BK, BS, GD-P, LN, and US collected the lake samples and analyzed the spatial and temporal occurrence of the urotrichs. TS and SF studied biogeographic patterns on datasets provided by JB and DB, and conducted logistic regression modeling and related urotrich genotypes to environmental conditions. MG did the ciliate drawings and additional morphometrics. All authors approved the final manuscript and contributed by writing their respective passages.

## Conflict of Interest

The authors declare that the research was conducted in the absence of any commercial or financial relationships that could be construed as a potential conflict of interest. The handling editor declared a past co-authorship with SF.

## Publisher’s Note

All claims expressed in this article are solely those of the authors and do not necessarily represent those of their affiliated organizations, or those of the publisher, the editors and the reviewers. Any product that may be evaluated in this article, or claim that may be made by its manufacturer, is not guaranteed or endorsed by the publisher.

## Footnotes

1
http://sagdb.uni-goettingen.de/detailedList.php?str_number=26.80

## Supplementary Material

Supplementary Figure 1 Algal strains used for feeding experiments with *U. castalia* (strain CIL-2017/25): **(A)** MS-2017/1 *Coelastrum* sp., **(B)** MS-2017/2 *Choricystis* sp., **(C)** MS-2017/7 *Acutodesmus obliquus*, **(D)** SAG 26.80 *Cryptomonas* sp., and **(E)** MS-2018/1 *Cosmarium* sp.; scale bar = 10 μm.

Supplementary Figure 2 Secondary structure of the SSU rRNA of *Urotricha castalia* (strain CIL-2017/25). The variable regions V4 and V9 are highlighted by gray boxes.

Supplementary Figure 3 Survival experiments with *Urotricha castalia* (strain CIL-2017/25) from Lake Mondsee, Austria. **(A)** Test of eight media plus SAG 26.80 *Cryptomonas* sp. as food source: Woods Hole MBL medium (WC), mixtures of WC:Volvic^®^ mineral water (V) in 1:1, 1:5, 5:1 v/v; Volvic^®^ mineral water only; modified Bourrelly medium; modified blue-green medium (BG11); sterile-filtered original lake water. **(B)** Test of five algal strains as food source in WC:V 5:1 v/v: MS-2017/1 *Coelastrum* sp., MS-2017/2 *Choricystis* sp., MS-2017/7 *Acutodesmus obliquus*, MS-2017/8 *Cosmarium* sp., and SAG 26.80 *Cryptomonas* sp.

Supplementary Figure 4 V9 secondary structures of the investigated *Urotricha* strains. The variable regions are marked by white boxes. The structures were calculated with mfold. The line graphic was drawn with PseudoViewer.

Supplementary Figure 5 Comparison of temporal and spatial distribution of *Urotricha* strains in Lake Mondsee between sequence data (V9 region) and morphology-based count data. Using the morphology-based approach, *U. agilis*, *U. castalia*, *U. furcata*, and *U. pseudofurcata* could be identified and counted. From the V9 dataset, only V9 markers from *U. agilis*, *U. castalia*_LM, and *U. furcata/U. pseudofurcata* could be extracted and used for the comparison.

Supplementary Figure 6 Comparison of temporal and spatial distribution of *Urotricha* strains in Lake Zurich between sequence data (V9 region) and morphology-based count data. Using the morphology-based approach, *U. agilis*, *U. castalia*, and *U. pseudofurcata* could be identified and counted. From the V9 dataset, only V9 markers from *U. agilis*, *U. castalia*_LM, and *U. furcata/U. pseudofurcata* could be extracted and used for the comparison.

Supplementary Figure 7 Ecological preferences of the investigated ciliate strains. Read abundances were correlated with and plotted against different environmental parameters. The black lines are regression lines and the gray area indicates the 95%-confidence intervals. Correlations were considered significant when the *p*-Value was <0.05.

Supplementary Table 1 Valid names of *Urotricha* species according to Bánki et al. (2021). Doubtful species were excluded (for details, see Foissner and Pfister, 1997).

Supplementary Table 2 Dataset information on the freshwater lakes used to investigate the distribution of *Urotricha* species under study.

Supplementary Table 3 Occurrence information (presence/absence) of *Urotricha* species in the investigated lakes. x indicates presence, 0 indicates absence, _V4 indicates when V4 barcode markers were used to screen the dataset. Unless indicated otherwise, only V9 barcode markers were used for the search.

Supplementary Table 4 Main morphometric data on the investigated *Urotricha* strains. Measurements in μm. CV = coefficient of variation in %, M = median, IV = *in vivo*, Max = maximum, Mean = arithmetic mean, Meth = applied methods, Min = minimum, n = number of specimens investigated, P = after protargol staining, QPS = after quantitative protargol staining, SD = standard deviation. Corresponding strains are color-coordinated. *Urotricha agilis* (CIL-2017/24, CIL-2019/10), *Urotricha furcata* (CIL-2019/13 and CIL-2019/6), *Urotricha pseudofurcata* (CIL-2019/3), and *Urotricha castalia* (CIL-2017/25, CIL-2017/27, and CIL-2019/1; CIL-48 is the original monoculture of *U. castalia* of which the clonal strain CIL-2019/1 derived).

Supplementary Table 5 Multiple linear regression models of the relationship of *U. agilis* and *U. furcata/U. pseudofurcata* abundances to environmental factors. Letter b refers to a regression coefficient.

## References

[B1] Adl S. M. Bass D. Lane C. E. Lukeš J. Schoch C. L. Smirnov A. (2019). Revisions to the classification, nomenclature, and diversity of eukaryotes. *J. Eukaryot. Microbiol.* 66 4–119. 10.1111/jeu.12691 30257078 PMC6492006

[B2] Agatha S. Ganser M. H. Santoferrara L. F. (2021). The importance of type species and their correct identification: a key example from Tintinnid ciliates (Alveolata, Ciliophora, Spirotricha). *J. Eukaryot. Microbiol.* 68:e12865. 10.1111/jeu.12865 34243218

[B3] Akaike H. (1974). A new look at the statistical model identification. *IEEE Trans. Automat. Contr.* 19 716–723. 10.1109/TAC.1974.1100705

[B4] Altschul S. F. Gish W. Myers E. W. Miller W. Lipman D. J. (1990). Basic local alignment search tool. *J. Mol. Biol.* 215, 403–410. 10.1016/S0022-2836(05)80360-2 2231712

[B5] Bánki O. Roskov Y. Döring M. Ower G. Vandepitte L. Hobern D. (2021). *Catalogue of Life Checklist (Version 2021-12-18)*. Catalogue of Life. 10.48580/d4tm 8892926

[B6] Boenigk J. Wodniok S. Bock C. Beisser D. Hempel C. Grossmann L. (2018). Geographic distance and mountain ranges structure freshwater protist communities on a European scale. *Metabarcoding Metagenom.* 2:e21519. 10.3897/mbmg.2.21519

[B7] Boscaro V. Santoferrara L. F. Zhang Q. Gentekaki E. Syberg-Olsen M. J. del Campo J. (2018). EukRef-Ciliophora: a manually curated, phylogeny-based database of small subunit rRNA gene sequences of ciliates. *Environ. Microbiol.* 20 2218–2230. 10.1111/1462-2920.14264 29727060

[B8] Bossard P. Gammeter S. Lehmann C. Bachofen R. Bürgi H.-R. Steiner D. (2001). Limnological description of the Lakes Zürich, Lucerne, and Cadagno. *Aquat. Sci.* 63 225–249. 10.1007/PL00001353

[B9] Byun Y. Han K. (2006). PseudoViewer. web application and web service for visualizing RNA pseudoknots and secondary structures. *Nucleic Acids Res.* 34 W416–W422. 10.1093/nar/gkl210 16845039 PMC1538805

[B10] Coleman A. W. (2000). The significance of a coincidence between evolutionary landmarks found in mating affinity and a DNA sequence. *Protist* 151 1–9. 10.1078/1434-4610-00002 10896128

[B11] Coleman A. W. (2003). ITS2 is a double-edged tool for eukaryote evolutionary comparisons. *Trends Genet.* 19 370–375. 10.1016/S0168-9525(03)00118-5 12850441

[B12] Coleman A. W. (2005). *Paramecium aurelia* revisited. *J. Eukaryot. Microbiol.* 52 68–77. 10.1111/j.1550-7408.2005.3327r.x 15702983

[B13] Darienko T. Rad-Menéndez C. Campbell C. Pröschold T. (2019). Are there any true marine *Chlorella* species? Molecular phylogenetic assessment and ecology of marine *Chlorella* -like organisms, including a description of *Droopiella* gen. nov. *Syst. Biodivers.* 17 811–829. 10.1080/14772000.2019.1690597 32256217 PMC7077435

[B14] de Vargas C. Audic S. Henry N. Decelle J. Mahé F. Logares R. (2015). Eukaryotic plankton diversity in the sunlit ocean. *Science* 348:1261605. 10.1126/science.1261605 25999516

[B15] del Campo J. Kolisko M. Boscaro V. Santoferrara L. F. Nenarokov S. Massana R. (2018). EukRef: phylogenetic curation of ribosomal RNA to enhance understanding of eukaryotic diversity and distribution. *PLoS Biol.* 16:e2005849. 10.1371/journal.pbio.2005849 30222734 PMC6160240

[B16] Dias R. J. P. de Souza P. M. Rossi M. F. Wieloch A. H. da Silva-Neto I. D. D’Agosto M. (2021). Ciliates as bioindicators of water quality: a case study in the neotropical region and evidence of phylogenetic signals (18S-rDNA). *Environ. Pollut.* 268:115760. 10.1016/j.envpol.2020.115760 33162216

[B17] Diehl S. (2003). The evolution and maintenance of omnivory: dynamic constraints and the role of food quality. *Ecology* 84 2557–2567. 10.1890/02-0399

[B18] Dokulil M. T. Jagsch A. Glen D. G. Anneville O. Jankowski T. Wahl B. (2006). Twenty years of spatially coherent deepwater warming in lakes across Europe related to the North Atlantic Oscillation. *Limnol. Oceanogr.* 51 2787–2793. 10.4319/lo.2006.51.6.2787

[B19] Dunthorn M. Klier J. Bunge J. Stoeck T. (2012). Comparing the hyper-variable V4 and V9 regions of the small subunit rDNA for assessment of ciliate environmental diversity. *J. Eukaryot. Microbiol.* 59 185–187. 10.1111/j.1550-7408.2011.00602.x 22236102

[B20] Fernandéz-Leborans G. M. Novillo A. (1994). Three new species of ciliate in the genera *Pseudocohnilembus*, *Pleuronema*, and *Urotricha* (Ciliophora). *Proc. Biol. Soc. Wash.* 107 221–238.

[B21] Ficker H. Luger M. Gassner H. (2017). From dimictic to monomictic. Empirical evidence of thermal regime transitions in three deep alpine lakes in Austria induced by climate change. *Freshw. Biol.* 62 1335–1345. 10.1111/fwb.12946

[B22] Foissner W. (1979). Ökologische und systematische Studien über das Neuston alpiner Kleingewässer, mit besonderer Berücksichtigung der Ciliaten. *Int. Rev. Hydrobiol. Hydrogr.* 64 99–140. 10.1002/iroh.19790640109

[B23] Foissner W. (1983). Taxonomische studien über die ciliaten des Großglocknergebietes (Hohe Tauern, Österreich) I. familien holophryidae, prorodontidae, plagiocampidae, colepidae, enchelyidae und Lacrymariidae nov. fam. *Ann. Nat. Mus. Wien* 84 49–85.

[B24] Foissner W. (1991). Basic light and scanning electron microscopic methods for taxonomic studies of ciliated protozoa. *Eur. J. Protistol.* 27 313–330. 10.1016/S0932-4739(11)80248-8 23194842

[B25] Foissner W. (2012). *Urotricha spetai* nov. spec., a new plankton ciliate (Ciliophora, Prostomatea) from a fishpond in the Seidlwinkel Valley, Rauris, Austrian Central Alps. *Verh. Zool. Bot. Ges. Österreich* 148/149 173–184.

[B26] Foissner W. (2014). An update of ‘basic light and scanning electron microscopic methods for taxonomic studies of ciliated protozoa’. *Int. J. Syst. Evol. Microbiol.* 64 271–292. 10.1099/ijs.0.057893-0 24198058

[B27] Foissner W. O’Donoghue P. J. (1990). Morphology and infraciliature of some freshwater ciliates (Protozoa: ciliophora) from Western and South Australia. *Invertebr. Taxon.* 3 661–696.

[B28] Foissner W. Pfister G. (1997). Taxonomic and ecologic revision of urotrichs (Ciliophora, Prostomatida) with three or more caudal cilia, including a user-friendly key. *Limnologica* 27 311–347.

[B29] Foissner W. Berger H. Kohmann F. (1994). *Taxonomische und ökologische Revision der Ciliaten des Saprobiensystems. Band III: Hymenostomata, Prostomatida, Nassulida.* Munich: Informationsberichte des Bayer. Landesamtes für Wasserwirtschaft.

[B30] Foissner W. Berger H. Schaumburg J. (1999). *Identification and Ecology of Limnetic Plankton Ciliates.* Munich: Landesamt für Wasserwirtschaft.

[B31] Foissner W. Strüder-Kypke M. van der Staay G. W. M. Moon-van der Staay S.-Y. Hackstein J. H. P. (2003). Endemic ciliates (Protozoa, Ciliophora) from tank bromeliads (Bromeliaceae): a combined morphological, molecular, and ecological study. *Eur. J. Protistol.* 39 365–372. 10.1078/0932-4739-00005

[B32] Forster D. Qu Z. Pitsch G. Bruni E. P. Kammerlander B. Pröschold T. (2021). Lake ecosystem robustness and resilience inferred from a climate-stressed protistan plankton network. *Microorganisms* 9:549. 10.3390/microorganisms9030549 PMC8001626 33800927

[B33] Gao F. Huang J. Zhao Y. Li L. Liu W. Miao M. (2017). Systematic studies on ciliates (Alveolata, Ciliophora) in China. Progress and achievements based on molecular information. *Eur. J. Protistol.* 61 409–423. 10.1016/j.ejop.2017.04.009 28545995

[B34] Gao F. Warren A. Zhang Q. Gong J. Miao M. Sun P. (2016). The all-data-based evolutionary hypothesis of ciliated protists with a revised classification of the Phylum Ciliophora (Eukaryota, Alveolata). *Sci. Rep.* 6:24874. 10.1038/srep24874 27126745 PMC4850378

[B35] Geisen S. (2016). The bacterial-fungal energy channel concept challenged by enormous functional versatility of soil protists. *Soil Biol. Biochem.* 102 22–25. 10.1016/j.soilbio.2016.06.013

[B36] Gimmler A. Korn R. de Vargas C. Audic S. Stoeck T. (2016). The Tara Oceans voyage reveals global diversity and distribution patterns of marine planktonic ciliates. *Sci. Rep.* 6:33555. 10.1038/srep33555 27633177 PMC5025661

[B37] Grattepanche J.-D. Katz L. A. (2020). Top-down and bottom-up controls on microeukaryotic diversity (i.e., amplicon analyses of SAR lineages) and function (i.e., metatranscriptome analyses) assessed in microcosm experiments. *Front. Mar. Sci.* 6:e2182. 10.3389/fmars.2019.00818

[B38] Guillard R. R. L. Lorenzen C. J. (1972). Yellow-green algae with chlorophyllide C 2. *J. Phycol.* 8 10–14. 10.1111/j.1529-8817.1972.tb03995.x

[B39] Gurdebeke P. R. Mertens K. N. Takano Y. Yamaguchi A. Bogus K. Dunthorn M. (2018). The affiliation of *Hexasterias problematica* and *Halodinium verrucatum* sp. nov. to ciliate cysts based on molecular phylogeny and cyst wall composition. *Eur. J. Protistol.* 66 115–135. 10.1016/j.ejop.2018.09.002 30261410

[B40] Hindák F. (1970). Culture collection of algae at laboratory of algology in trebon. *Arch. Hydrobiol. Algol. Stud.* 2/3(Suppl. 39) 86–126.

[B41] Holyoak M. Sachdev S. (1998). Omnivory and the stability of simple food webs. *Oecologia* 117 413–419. 10.1007/s004420050675 28307921

[B42] Kahl A. (1930). “Urtiere oder Protozoa I: wimpertiere oder Ciliata (Infusoria). Eine Bearbeitung der freilebenden und ectocommensalen Infusorien der Erde, unter Ausschluß der marinen Tintinnidae. 1. Allgemeiner Teil und Prostomata,” in *Die Tierwelt Deutschlands und der Angrenzenden Meeresteile Nach Ihren Merkmalen und Nach Ihrer Lebensweise*, ed. Dahl F. (Jena: G. Fischer), 1–180.

[B43] Kammerlander B. Koinig K. A. Rott E. Sommaruga R. Tartarotti B. Trattner F. (2016). Ciliate community structure and interactions within the planktonic food web in two alpine lakes of contrasting transparency. *Freshw. Biol.* 61 1950–1965. 10.1111/fwb.12828 27840457 PMC5082529

[B44] Krainer K.-H. (1995). Taxonomische Untersuchungen an neuen und wenig bekannten planktischen Ciliaten (Protozoa: ciliophora) aus Baggerseen in Österreich. *Lauterbornia* 21 39–68.

[B45] Krenek S. Berendonk T. U. Fokin S. I. (2015). New Paramecium (Ciliophora, Oligohymenophorea) congeners shape our view on its biodiversity. *Org. Divers. Evol.* 15 215–233. 10.1007/s13127-015-0207-9

[B46] Lara E. Berney C. Harms H. Chatzinotas A. (2007). Cultivation-independent analysis reveals a shift in ciliate 18S rRNA gene diversity in a polycyclic aromatic hydrocarbon-polluted soil. *FEMS Microbiol. Ecol.* 62 365–373. 10.1111/j.1574-6941.2007.00387.x 17949434

[B47] Lara E. Mitchell E. A. D. Moreira D. López Garcia P. (2011). Highly diverse and seasonally dynamic protist community in a pristine peat bog. *Protist* 162 14–32. 10.1016/j.protis.2010.05.003 20692868

[B48] Lynn D. H. (2004). Morphology or molecules: how do we identify the major lineages of ciliates (Phylum Ciliophora). *Eur. J. Protistol.* 39 356–364.

[B49] Lynn D. H. (2008). *The Ciliated Protozoa. Characterization, Classification, and Guide to the Literature.* Dordrecht: Springer.

[B50] Marin B. Palm A. Klingberg M. Melkonian M. (2003). Phylogeny and taxonomic revision of plastid-containg euglenophytes based on SSU rDNA sequence comparisons and synapomorphic signatures in the SSU rRNA secondary structure. *Protist* 154 99–145. 10.1016/j.protis.2009.10.002 12812373

[B51] Massana R. Gobet A. Audic S. Bass D. Bittner L. Boutte C. (2015). Marine protist diversity in European coastal waters and sediments as revealed by high-throughput sequencing. *Environ. Microbiol.* 17 4035–4049. 10.1111/1462-2920.12955 26119494

[B52] Muñoz A. Téllez C. Fernández-Galiano D. (1987). Morphology and infraciliature in Urotricha nais sp. n. and *Urotricha castalia* sp. n. (Ciliophora, Prorodontida). *Acta Protozool.* 263 197–204. + Plate I.

[B53] Niedrist G. H. Psenner R. Sommaruga R. (2018). Climate warming increases vertical and seasonal water temperature differences and inter-annual variability in a mountain lake. *Clim. Change* 151 473–490. 10.1007/s10584-018-2328-6

[B54] Orsi W. Edgcomb V. Faria J. Foissner W. Fowle W. H. Hohmann T. (2012). Class Cariacotrichea, a novel ciliate taxon from the anoxic Cariaco Basin, Venezuela. *Int. J. Syst. Evol. Microbiol.* 62 1425–1433. 10.1099/ijs.0.034710-0 21841005

[B55] Párducz B. (1967). Ciliary movement and coordination in ciliates. *Int. Rev. Cytol.* 21 91–128. 10.1016/S0074-7696(08)60812-8 4961084

[B56] Pawlowski J. Lejzerowicz F. Esling P. (2014). Next-generation environmental diversity surveys of foraminifera: preparing the future. *Biol. Bull.* 227 93–106. 10.1086/BBLv227n2p93 25411369

[B57] Penard E. (1922). *Études sur les Infusoires d’eau Douce.* Genève: Georg & Cie.

[B58] Pfister G. Auer B. Arndt H. (2002). Pelagic ciliates (Protozoa, Ciliophora) of different brackish and freshwater lakes - a community analysis at the species level. *Limnologica* 32 147–168. 10.1016/S0075-9511(02)80005-6

[B59] Piredda R. Claverie J.-M. Decelle J. de Vargas C. Dunthorn M. Edvardsen B. (2018). Diatom diversity through HTS-metabarcoding in coastal European seas. *Sci. Rep.* 8:18059. 10.1038/s41598-018-36345-9 30584235 PMC6305388

[B60] Pitsch G. Bruni E. P. Forster D. Qu Z. Sonntag B. Stoeck T. (2019). Seasonality of planktonic freshwater ciliates: are analyses based on V9 regions of the 18S rRNA gene correlated with morphospecies counts? *Front. Microbiol.* 10:248. 10.3389/fmicb.2019.00248 30837972 PMC6389714

[B61] Posch T. Eugster B. Pomati F. Pernthaler J. Pitsch G. Eckert E. M. (2015). Network of interactions between ciliates and phytoplankton during spring. *Front. Microbiol.* 6:1289. 10.3389/fmicb.2015.01289 26635757 PMC4653745

[B62] Pringsheim E. G. (1946). *Pure Cultures of Algae. Their Preparation and Maintenance.* London: Cambridge University Press.

[B63] Pröschold T. Rieser D. Darienko T. Kammerlander B. Pitsch G. Bruni E. P. (2021). An integrative approach sheds new light onto the systematics and ecology of the widespread ciliate genus Coleps (Ciliophora, Prostomatea). *Sci. Rep.* 11:5916. 10.1038/s41598-021-84265-y 33723272 PMC7960993

[B64] Qu Z. Forster D. Bruni E. P. Frantal D. Kammerlander B. Nachbaur L. (2021). Aquatic food webs in deep temperate lakes. Key species establish through their autecological versatility. *Mol. Ecol.* 30 1053–1071. 10.1111/mec.15776 33306859

[B65] Rippka R. Herdman H. (1992). *Pasteur Culture Collection of Cyanobacteria Catalogue & Taxonomic Handbook. 1. Catalogue of Strains.* Paris: Institute Pasteur.

[B66] Schewiakoff W. (1892). Ueber die geographische verbreitung der süsswasser-protozoen. *Verh. Naturw. Med. Ver. Heidelb. (N. S.)* 4 544–567.

[B67] Schewiakoff W. (1893). Über die geographische verbreitung der süsswasser-protozoën. *Zap. Imp. Akad. Nauk SSSR (Sér. 7)* 41 1–201.

[B68] Schlösser U. C. (1997). Additions to the culture collection of algae since 1994. *Bot. Acta* 110 424–429. 10.1111/j.1438-8677.1997.tb00659.x

[B69] Shazib S. U. A. Vd’ačný P. Kim J. H. Jang S. W. Shin M. K. (2016). Molecular phylogeny and species delimitation within the ciliate genus *Spirostomum* (Ciliophora, Postciliodesmatophora, Heterotrichea), using the internal transcribed spacer region. *Mol. Phylogen. Evol.* 102 128–144. 10.1016/j.ympev.2016.05.041 27261253

[B70] Sichrowsky U. Schabetsberger R. Sonntag B. Stoyneva M. Maloney A. E. Nelson D. B. (2014). Limnological characterization of volcanic crater lakes on Uvea Island (Wallis and Futuna, South Pacific). *Pac. Sci.* 68 333–343. 10.2984/68.3.3

[B71] Šimek K. Grujčić V. Nedoma J. Jezberová J. Šorf M. Anna Matoušů A. (2019). Microbial food webs in hypertrophic fishponds: omnivorous ciliate taxa are major protistan bacterivores. *Limnol. Oceanogr.* 64 2295–2309. 10.1002/lno.11260

[B72] Skibbe O. (1994). An improved quantitative protargol stain for ciliates and other planktonic protists. *Arch. Hydrobiol.* 130 339–347.

[B73] Sonneborn T. M. (1975). The *Paramecium aurelia* complex of fourteen sibling species. *Trans. Am. Microsc. Soc.* 94 155–178.

[B74] Sonntag B. Foissner W. (2004). *Urotricha psenneri* n. sp. and *Amphileptus piger* (Vuxanovici, 1962) n. comb., two planktonic ciliates (Protozoa, Ciliophora) from an oligotrophic lake in Austria. *J. Eukaryot. Microbiol.* 51 670–677. 10.1111/j.1550-7408.2004.tb00607.x 15666725

[B75] Sonntag B. Kammerlander B. Summerer M. (2017). Bioaccumulation of ultraviolet sunscreen compounds (mycosporine-like amino acids) by the heterotrophic freshwater ciliate *Bursaridium* living in alpine lakes. *Inland Waters* 7 55–64. 10.1080/20442041.2017.1294348 28690781 PMC5478918

[B76] Sonntag B. Posch T. Klammer S. Teubner K. Psenner R. (2006). Phagotrophic ciliates and flagellates in an oligotrophic, deep, alpine lake: contrasting variability with seasons and depths. *Aquat. Microb. Ecol.* 43 193–207. 10.3354/ame043193

[B77] Sonntag B. Summerer M. Sommaruga R. (2011). Are freshwater mixotrophic ciliates less sensitive to solar UV radiation than heterotrophic ones? *J. Eukaryot. Microbiol.* 58 196–202. 10.1111/j.1550-7408.2011.00540.x 21414057 PMC3182536

[B78] Spanner C. Darienko T. Biehler T. Sonntag B. Pröschold T. (2020). Endosymbiotic green algae in *Paramecium bursaria*: a new isolation method and a simple diagnostic PCR approach for the identification. *Diversity* 12:240. 10.3390/d12060240

[B79] Stern R. Kraberg A. Bresnan E. Kooistra W. H. C. F. Lovejoy C. Montresor M. (2018). Molecular analyses of protists in long-term observation programmes – current status and future perspectives. *J. Plankton Res.* 40 519–536. 10.1093/plankt/fby035

[B80] Stoeck T. Kochems R. Forster D. Lejzerowicz F. Pawlowski J. (2018). Metabarcoding of benthic ciliate communities shows high potential for environmental monitoring in salmon aquaculture. *Ecol. Indic.* 85, 153–164. 10.1016/j.ecolind.2017.10.04

[B81] Stoeck T. Przybos E. Dunthorn M. (2014a). The D1-D2 region of the large subunit ribosomal DNA as barcode for ciliates. *Mol. Ecol. Resour.* 14 458–468. 10.1111/1755-0998.12195 24165195

[B82] Stoeck T. Breiner H.-W. Filker S. Ostermaier V. Kammerlander B. Sonntag B. (2014b). A morphogenetic survey on ciliate plankton from a mountain lake pinpoints the necessity of lineage-specific barcode markers in microbial ecology. *Environ. Microbiol.* 16 430–444. 10.1111/1462-2920.12194 23848238 PMC4208686

[B83] Stokes A. C. (1886). Some new infusoria from American fresh waters. *Ann. Mag. Nat. Hist.* 17 98–112. 10.1080/00222938609460121 + Plate I,

[B84] Swofford D. L. (2002). *PAUP* Phylogenetic Analysis Using Parsimony (*and Other Methods), Version 4.0b10.* Sunderland, MA: Sinauer Associates.

[B85] Tanabe A. S. Nagai S. Hida K. Yasuike M. Fujiwara A. Nakamura Y. (2016). Comparative study of the validity of three regions of the 18S-rRNA gene for massively parallel sequencing-based monitoring of the planktonic eukaryote community. *Mol. Ecol. Res.* 16 402–414. 10.1111/1755-0998.12459 26309223

[B86] Tirok K. Gaedke U. (2006). Spring weather determines the relative importance of ciliates, rotifers and crustaceans for the initiation of the clear-water phase in a large, deep lake. *J. Plankton Res.* 28 361–373. 10.1093/plankt/fbi121

[B87] Van Wichelen J. Johansson L. S. Vanormelingen P. Declerck S. A. J. Torben L. Lauridsen T. L. (2013). Planktonic ciliate community structure in shallow lakes of lowland Western Europe. *Eur. J. Protistol.* 49 538–551. 10.1016/j.ejop.2013.06.001 23890772

[B88] Warren A. Patterson D. J. Dunthorn M. Clamp J. C. Achilles-Day U. E. M. Aescht E. (2017). Beyond the “Code”: a guide to the description and documentation of biodiversity in ciliated protists (Alveolata, Ciliophora). *J. Eukaryot. Microbiol.* 64 539–554. 10.1111/jeu.12391 28061024 PMC5697677

[B89] Weisse T. Frahm A. (2001). Species-specific interactions between small planktonic ciliates (*Urotricha* spp.) and rotifers (*Keratella* spp.). *J. Plankton Res.* 23 1329–1338. 10.1093/plankt/23.12.1329

[B90] Weisse T. Frahm A. (2002). Direct and indirect impact of two common rotifer species (*Keratella* spp.) on two abundant ciliate species (*Urotricha furcata*, *Balanion planctonicum*). *Freshw. Biol.* 47 53–64. 10.1046/j.1365-2427.2002.00780.x

[B91] Weisse T. Anderson R. Arndt H. Calbet A. Hansen P. J. Montagnes D. J. S. (2016). Functional Ecology of aquatic phagotrophic protists – concepts, limitations, and perspectives. *Eur. J. Protistol.* 55 50–74. 10.1016/j.ejop.2016.03.003 27094869

[B92] Weisse T. Karstens N. Meyer V. C. L. Janke L. Lettner S. Teichgräber K. (2001). Niche separation in common prostome freshwater ciliates: the effect of food and temperature. *Aquat. Microb. Ecol.* 26 167–179. 10.3354/ame026167

[B93] Weisse T. Müller H. Pinto-Coelho R. M. Schweizer A. Springmann D. Baldringer G. (1990). Response of the microbial loop to the phytoplankton spring bloom in a large prealpine lake. *Limnol. Oceanogr.* 35 781–794.

[B94] Wolf M. Friedrich J. Dandekar T. Müller T. (2005). CBCAnalyzer. inferring phylogenies based on compensatory base changes in RNA secondary structures. *In Silico Biol.* 5 291–294. 15996120

[B95] Yankova Y. Neuenschwander S. Köster O. Posch T. (2017). Abrupt stop of deep water turnover with lake warming. Drastic consequences for algal primary producers. *Sci. Rep.* 7:13770. 10.1038/s41598-017-13159-9 29062037 PMC5653828

[B96] Zhan Z. Li J. Xu K. (2019). Ciliate environmental diversity can be underestimated by the V4 region of SSU rDNA. Insights from species delimitation and multilocus phylogeny of *Pseudokeronopsis* (Protist, Ciliophora). *Microorganisms* 7:493. 10.3390/microorganisms7110493 31717798 PMC6920991

[B97] Zhang Q. Yi Z. Fan X. Warren A. Gong J. Song W. (2014). Further insights into the phylogeny of two ciliate classes Nassophorea and Prostomatea (Protista, Ciliophora). *Mol. Phylogenet. Evol.* 70 162–170. 10.1016/j.ympev.2013.09.015 24075983

[B98] Zhu F. Massana R. Not F. Marie D. Vaulot D. (2005). Mapping of picoeucaryotes in marine ecosystems with quantitative PCR of the 18S rRNA gene. *FEMS Microbiol. Ecol.* 52 79–92. 10.1016/j.femsec.2004.10.006 16329895

[B99] Zuker M. (2003). Mfold web server for nucleic acid folding and hybridization prediction. *Nucleic Acids Res.* 31 3406–3415. 10.1093/nar/gkg595 12824337 PMC169194

