# Supplementary figures and images for: Molecular Data Reveal a Cryptic Diversity in the Genus Urotricha (Alveolata, Ciliophora, Prostomatida), a Key Player in Freshwater Lakes, With Remarks on Morphology, Food Preferences, and Distribution
Source: Front Microbiol. 2022 Feb 4;12:787290. doi: 10.3389/fmicb.2021.787290 (PMC8854374; doi:10.3389/fmicb.2021.787290)

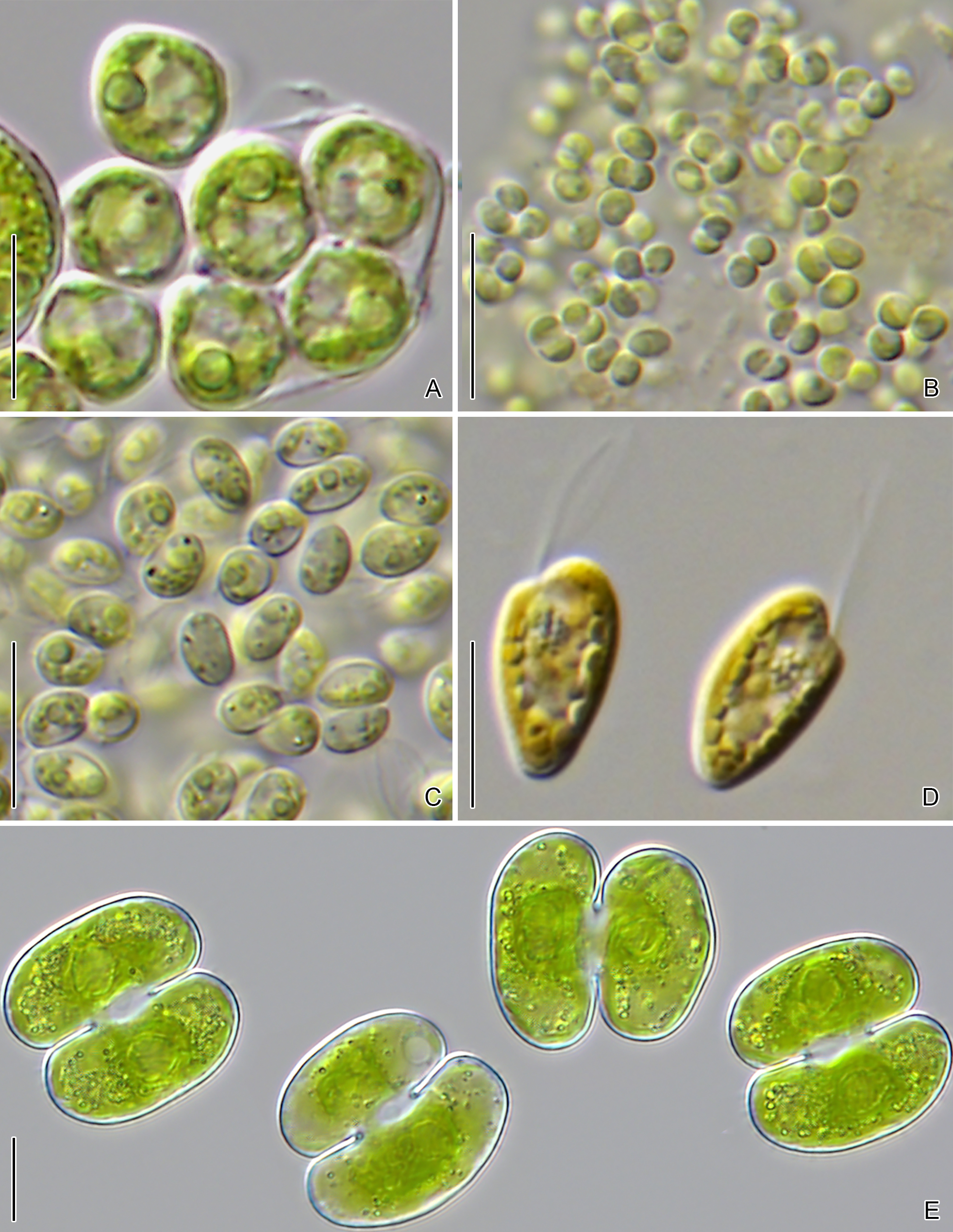

Supplement: Supplementary Figure 1 — Algal strains used for feeding experiments with U. castalia (strain CIL-2017/25): (A) MS-2017/1 Coelastrum sp., (B) MS-2017/2 Choricystis sp., (C) MS-2017/7 Acutodesmus obliquus, (D) SAG 26.80 Cryptomonas sp., and (E) MS-2018/1 Cosmarium sp.; scale bar = 10 μm. [file Image_1.JPEG]

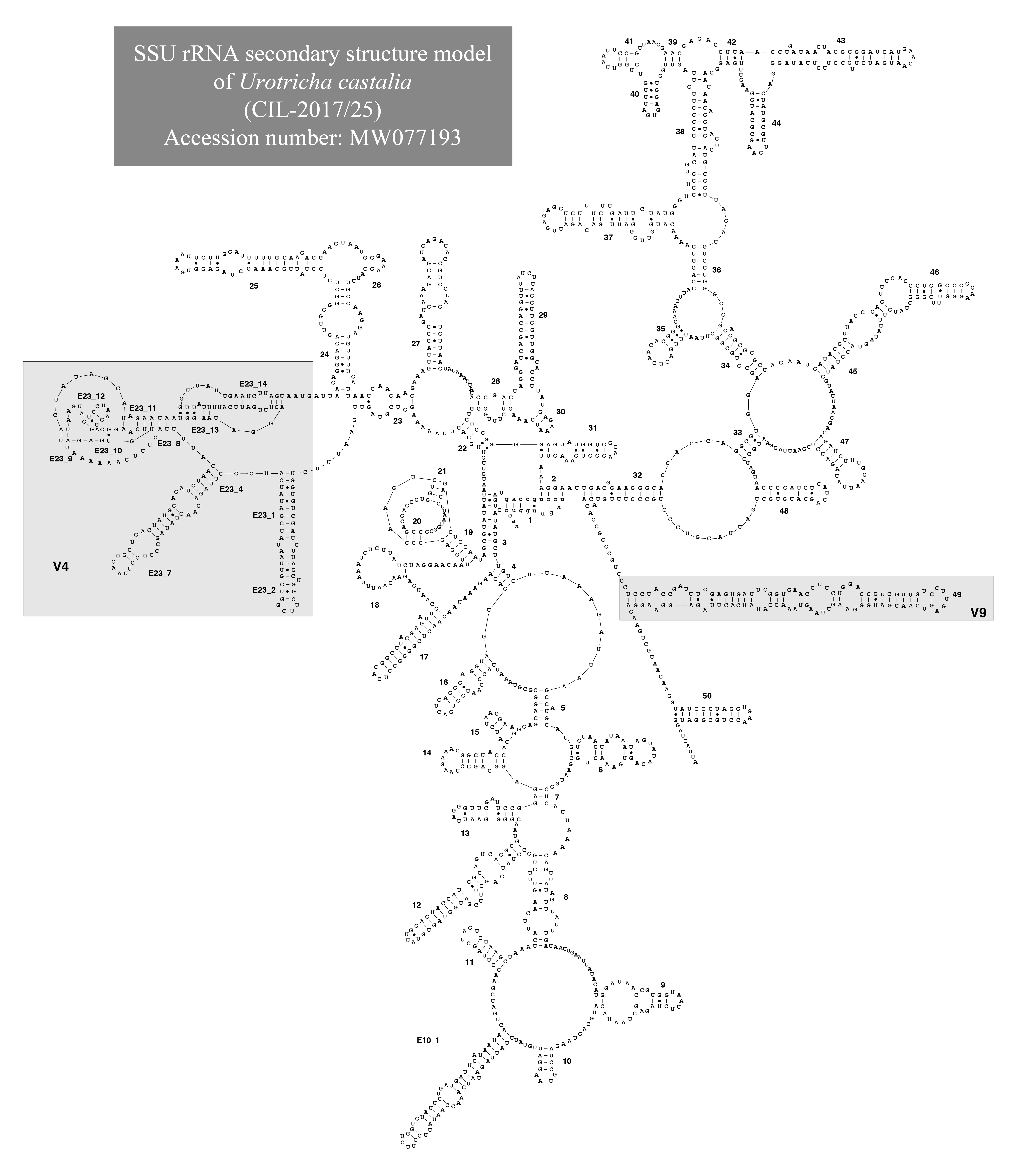

Supplement: Supplementary Figure 2 — Secondary structure of the SSU rRNA of Urotricha castalia (strain CIL-2017/25). The variable regions V4 and V9 are highlighted by gray boxes. [file Image_2.TIF]

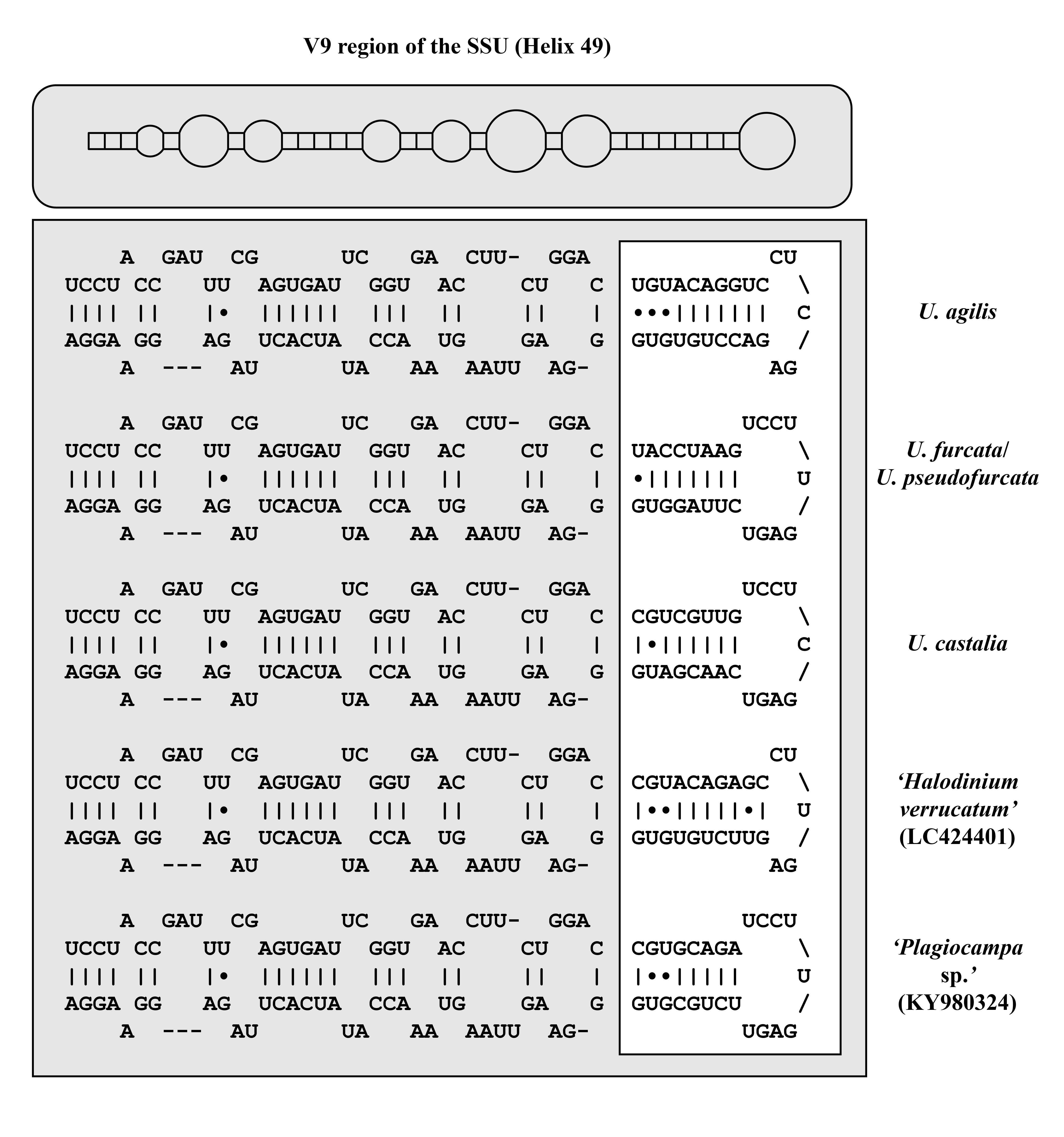

Supplement: Supplementary Figure 3 — Survival experiments with Urotricha castalia (strain CIL-2017/25) from Lake Mondsee, Austria. (A) Test of eight media plus SAG 26.80 Cryptomonas sp. as food source: Woods Hole MBL medium (WC), mixtures of WC:Volvic® mineral water (V) in 1:1, 1:5, 5:1 v/v; Volvic® mineral water only; modified Bourrelly medium; modified blue-green medium (BG11); sterile-filtered original lake water. (B) Test of five algal strains as food source in WC:V 5:1 v/v: MS-2017/1 Coelastrum sp., MS-2017/2 Choricystis sp., MS-2017/7 Acutodesmus obliquus, MS-2017/8 Cosmarium sp., and SAG 26.80 Cryptomonas sp. [file Image_3.TIF]

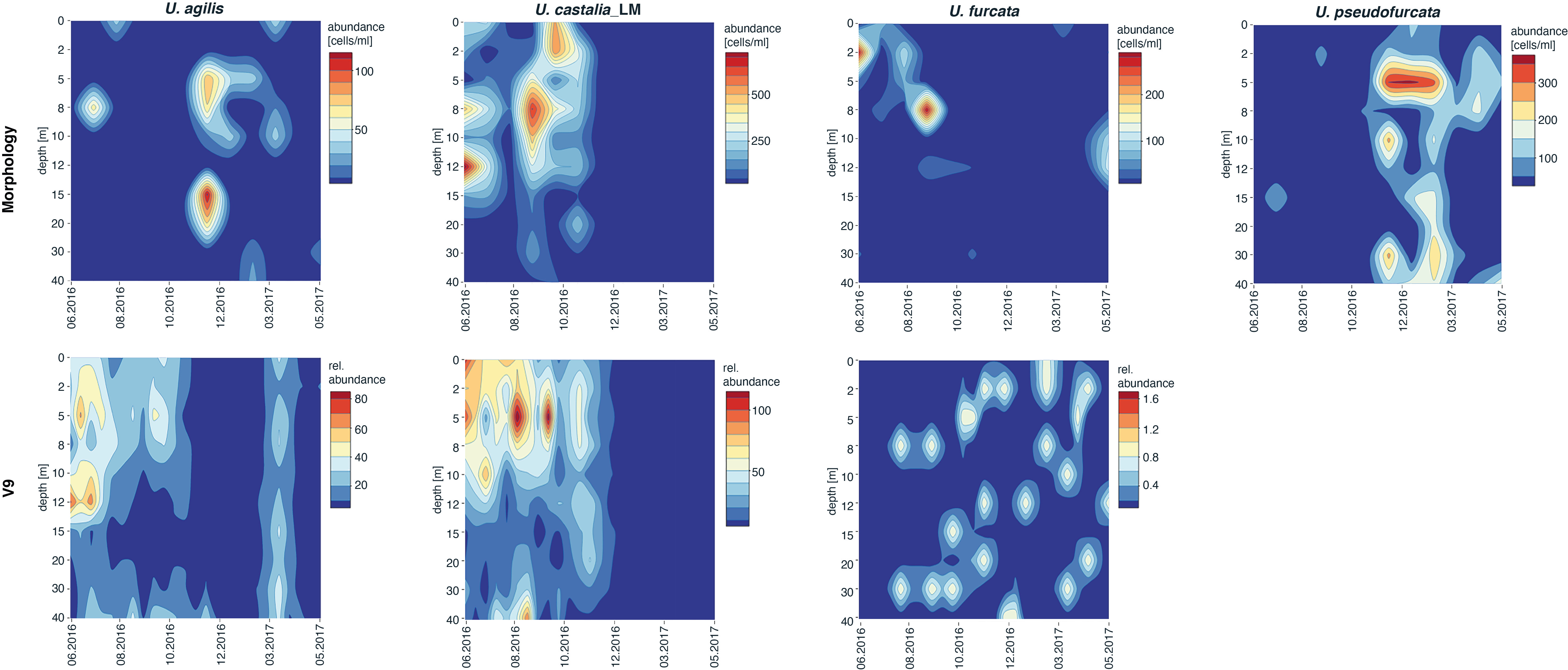

Supplement: Supplementary Figure 4 — V9 secondary structures of the investigated Urotricha strains. The variable regions are marked by white boxes. The structures were calculated with mfold. The line graphic was drawn with PseudoViewer. [file Image_4.JPEG]

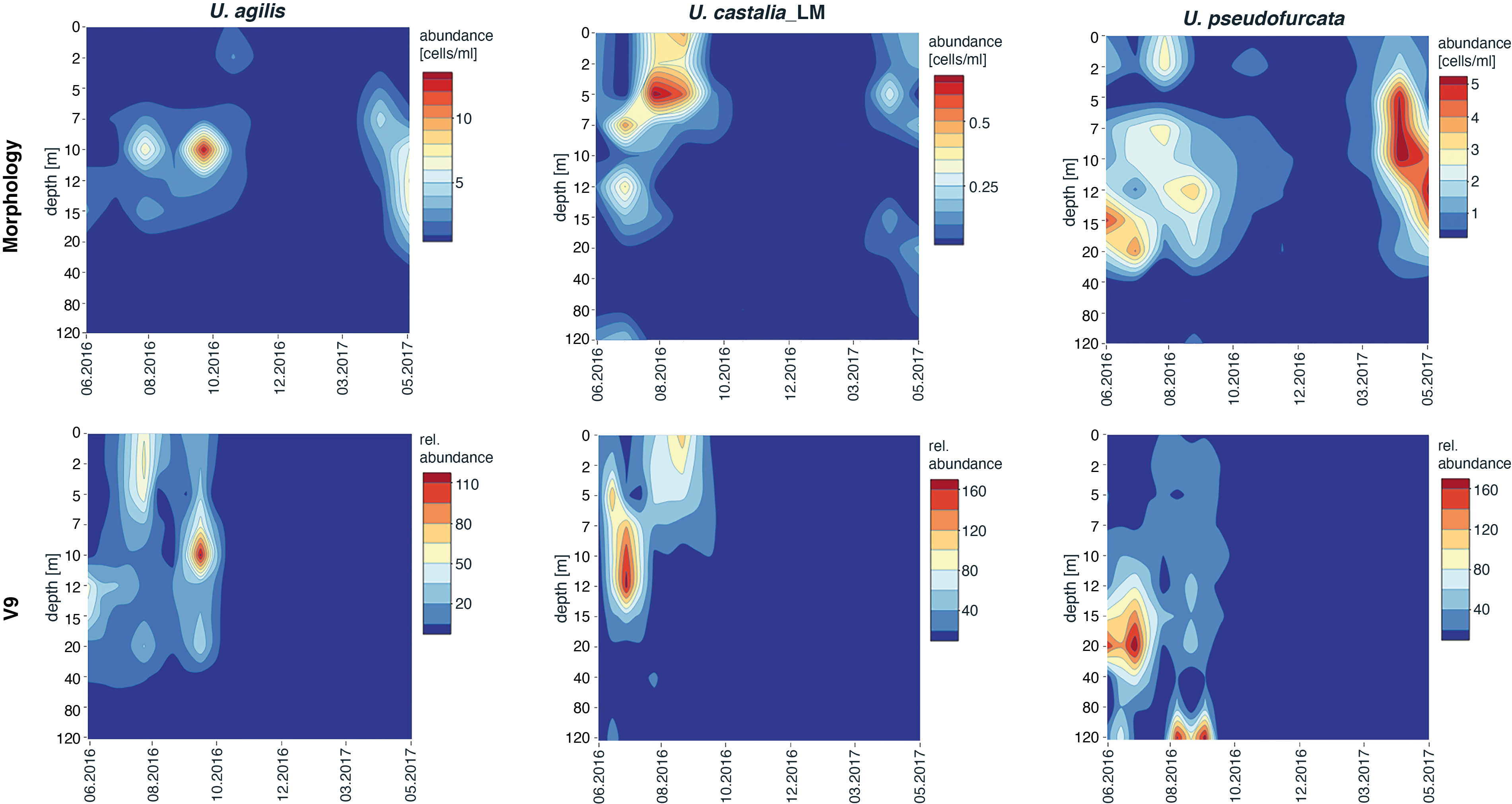

Supplement: Supplementary Figure 5 — Comparison of temporal and spatial distribution of Urotricha strains in Lake Mondsee between sequence data (V9 region) and morphology-based count data. Using the morphology-based approach, U. agilis, U. castalia, U. furcata, and U. pseudofurcata could be identified and counted. From the V9 dataset, only V9 markers from U. agilis, U. castalia_LM, and U. furcata/U. pseudofurcata could be extracted and used for the comparison. [file Image_5.JPEG]

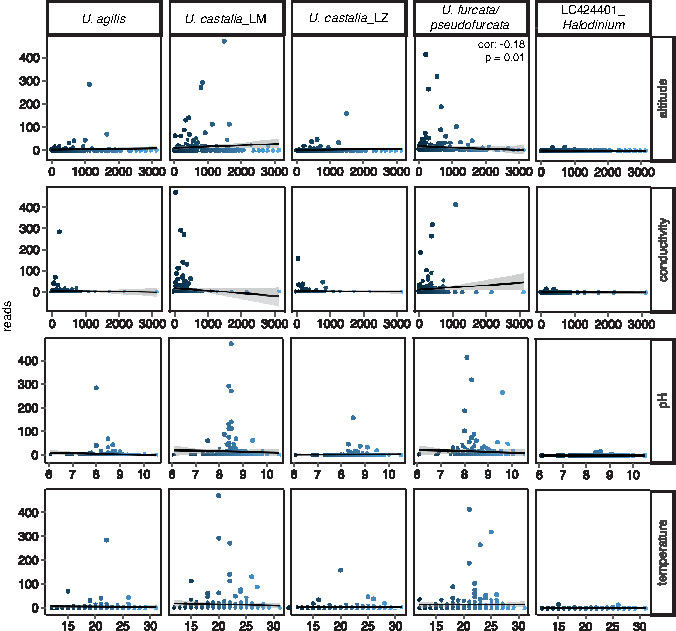

Supplement: Supplementary Figure 6 — Comparison of temporal and spatial distribution of Urotricha strains in Lake Zurich between sequence data (V9 region) and morphology-based count data. Using the morphology-based approach, U. agilis, U. castalia, and U. pseudofurcata could be identified and counted. From the V9 dataset, only V9 markers from U. agilis, U. castalia_LM, and U. furcata/U. pseudofurcata could be extracted and used for the comparison. [file Image_6.JPEG]

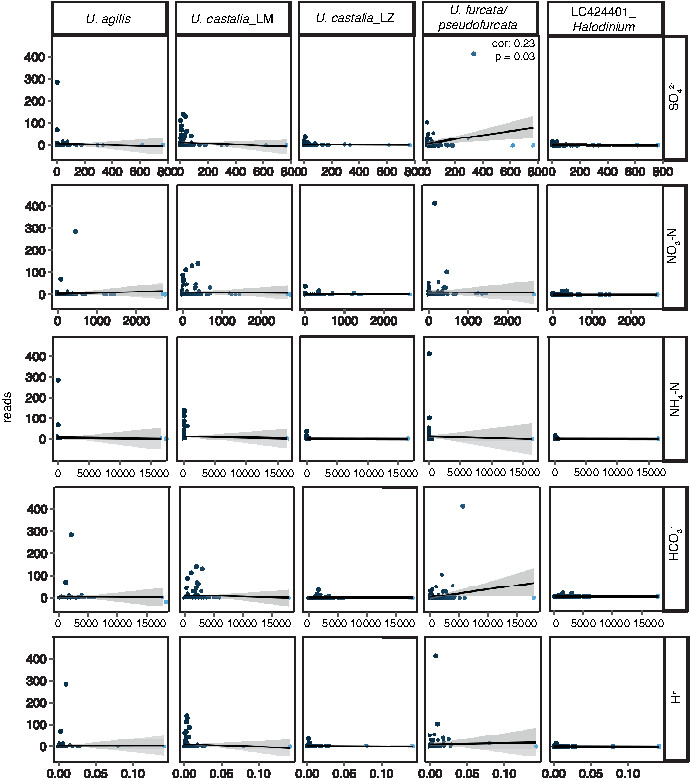

Supplement: Supplementary Figure 7 — Ecological preferences of the investigated ciliate strains. Read abundances were correlated with and plotted against different environmental parameters. The black lines are regression lines and the gray area indicates the 95%-confidence intervals. Correlations were considered significant when the p-Value was <0.05. [file Image_7.JPEG]

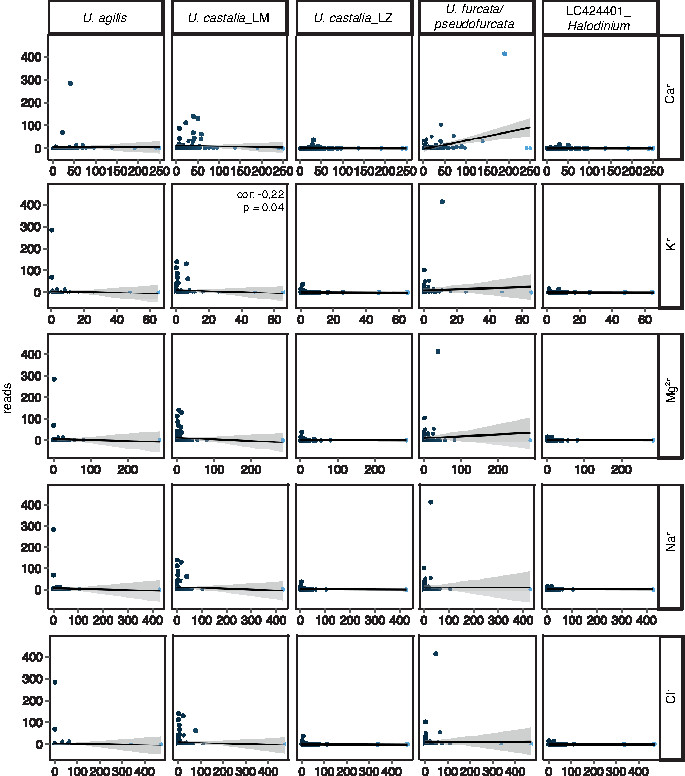

Supplement: Supplementary file 13 [file Image_8.JPEG]

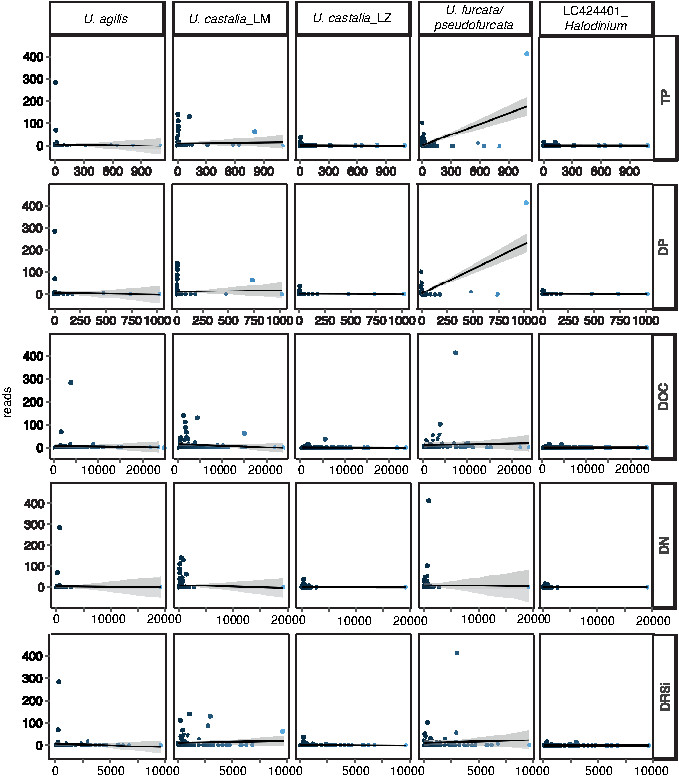

Supplement: Supplementary file 14 [file Image_9.JPEG]

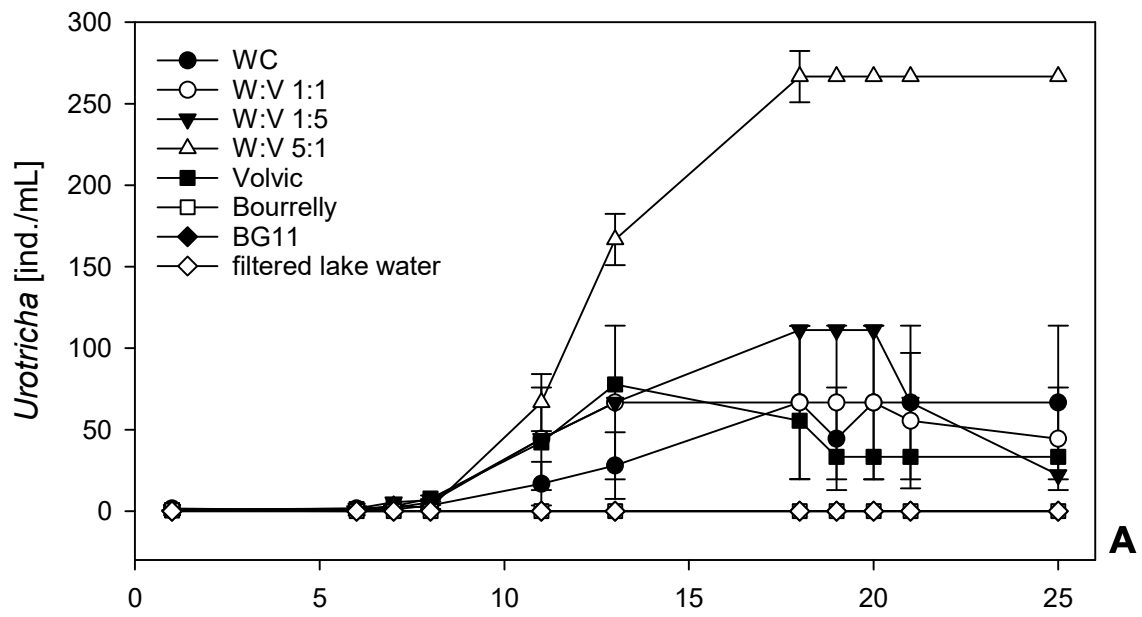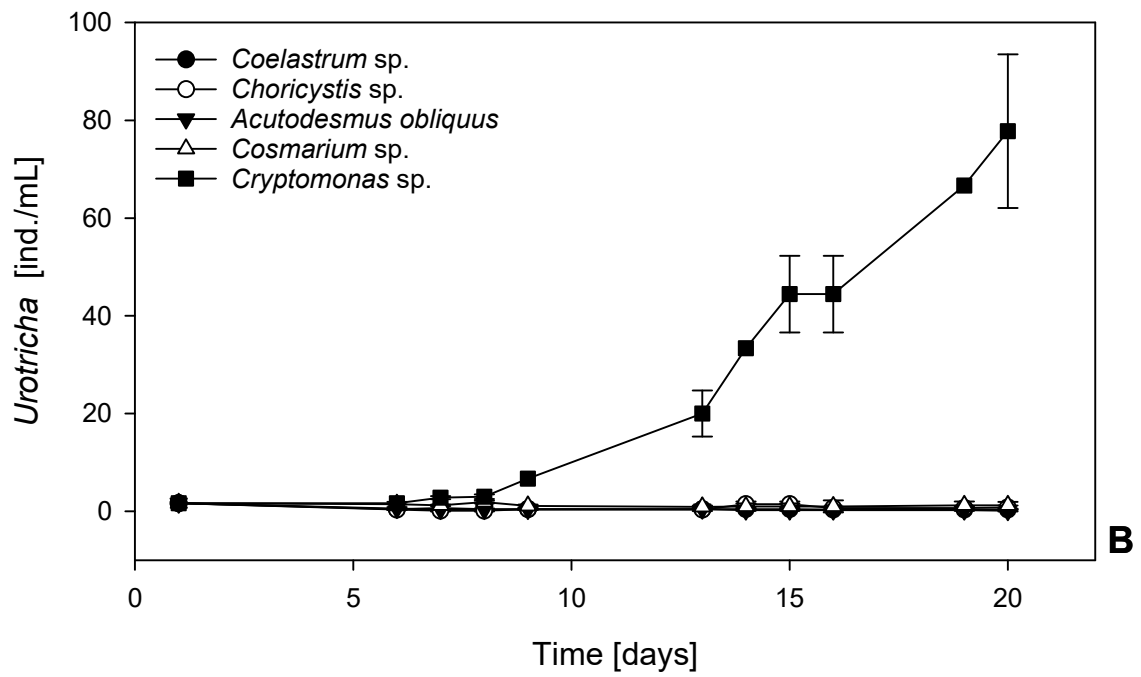

Supplement: Supplementary file 15 [file Image_10.PDF]
