# Supplementary material for: Molecular Data Reveal a Cryptic Diversity in the Genus Urotricha (Alveolata, Ciliophora, Prostomatida), a Key Player in Freshwater Lakes, With Remarks on Morphology, Food Preferences, and Distribution
Source: Front Microbiol. 2022 Feb 4;12:787290. doi: 10.3389/fmicb.2021.787290 (PMC8854374; doi:10.3389/fmicb.2021.787290)
Supplement: Supplementary Table 1 — Valid names of Urotricha species according to Bánki et al. (2021). Doubtful species were excluded (for details, see Foissner and Pfister, 1997). [file Table_1.DOCX]

| **Accepted name** | **Possible synonym** | **Comments** | **Caudal**  **Cilia #** | **Habitat** |
| --- | --- | --- | --- | --- |
| *Urotricha agilis* (Stokes, 1886) Kahl, 1930 | *Urotricha gyrans* (Stokes, 1887) Foissner. 1979 |  | 1 | freshwater |
|  | *Urotricha nais* Muñoz et al., 1987 |  | 1 | freshwater |
| *Urotricha baikalensis* Alekperov et al., 2012 |  |  | 1 | freshwater |
| *Urotricha corlissiana* Song and Wilbert, 1989 | *Urotricha platystoma* Stokes, 1886 |  | 1 | freshwater |
| *Urotricha discolor* Kahl, 1930 |  |  | 1 | freshwater |
| *Urotricha dragescoi* Foissner, 1984 | *Urotricha armata* Kahl, 1927 sensu Dragesco et al., 1974 |  | 1 | freshwater |
| *Urotricha farcta* Claparède and Lachmann, 1859 | *Urotricha fareta* Claparède and Lachmann, 1859 (misspelling) |  | 1 | freshwater |
|  | *Urotricha gyrans* (Stokes, 1887) Foissner, 1979 |  | 1 | freshwater |
|  | *Urotricha minkewicki* Schouteden, 1906 |  | 1 | freshwater |
|  | *Urotricha minkewickzi* Schouteden, 1906 (misspelling) |  | 1 | freshwater |
|  | *Urotricha parvula* Penard, 1922 |  | 1 | freshwater |
| *Urotricha globosa* Schewiakoff, 1892 |  |  | 1 | freshwater |
| *Urotricha gracilis* Penard, 1922 |  |  | 1 | freshwater |
| *Urotricha lagenula* Ehrenberg-Kent, 1881 |  |  | 1 | freshwater |
| *Urotricha lemani* Foissner et al., 1994 | *Urotricha armata* Dragesco, 1960 | *Urotricha lemani* is the replacement name for the junior primary homonym *U. armata* Dragesco, 1960 | 1 | freshwater |
| *Urotricha nais* Muñoz et al., 1987 | Probably synonym of *Urotricha agilis* (Stokes, 1886) Kahl, 1930 |  | 1 | freshwater |
| *Urotricha ondina* Muñoz et al., 1989 |  |  | 1 | freshwater |
| *Urotricha ovata* Kahl, 1926 | Possibly, a synonym of *Urotricha farcta* Claparède and Lachmann, 1859 |  | 1 | freshwater |
| *Urotricha platystoma* Stokes, 1886 | *Urotricha armata*, Kahl, 1927 |  | 1 | freshwater |
|  | *Urotricha corlissiana* Song and Wilbert, 1989 |  | 1 | freshwater |
| *Urotricha psenneri* Sonntag and Foissner, 2004 |  |  | 1 | freshwater |
| *Urotricha pusilla* Penard, 1922 |  | Kahl (1930) doubted that this species was an *Urotricha* | 1 | freshwater |
| *Urotricha ristoi* Krainer, 1995 |  |  | 1 | freshwater |
| *Urotricha sphaerica* Grolière, 1977 |  |  | 1 | freshwater |
| *Urotricha synuraphaga* Kahl, 1927 |  |  | 1 | freshwater |
| *Urotricha vitrea* Martín-Gonzalez et al., 1985 |  |  | 1 | freshwater |
| *Urotricha furcata* Schewiakoff, 1892 |  |  | 2 | freshwater |
| *Urotricha macrostoma* Foissner, 1983 |  |  | 2 | freshwater |
| *Urotricha pseudofurcata* Krainer, 1995 |  |  | 2 | freshwater |
| *Urotricha cyrtonucleata* Martin and Montagnes, 1993 |  |  | 3 | marine,  brackish |
| *Urotricha spetai* Foissner, 2012 |  |  | 4 | freshwater |
| *Urotricha matthesi matthesi* Krainer, 1995 | *Urotricha matthesi*, Krainer, 1995 |  | 3-4 | freshwater |
| *Urotricha matthesi tristicha* Foissner and Pfister, 1997 |  |  | 4-5 | freshwater |
| *Urotricha tricha* Wang and Nie, 1933 |  |  | 4, rarely 5-6 | freshwater |
| *Urotricha castalia* Muñoz et al., 1987 | *Urotricha rotunda*, Fernandez-Leborans and Novillo, 1994 |  | 4-9 | freshwater |
| *Urotricha antarctica* Wilbert and Song, 2008 |  |  | ca. 6 | marine,  brackish |
| *Urotricha multisetosa* Wang & Nie, 1933 | *Urotricha faurei* Dragesco et al., 1974 |  | At least 10 | freshwater |
| *Urotricha apsheronica* Alekperov, 1984 |  |  | 12-16 | freshwater |
| *Urotricha pelagica* Kahl, 1935 |  | Redescribed by Foissner and Pfister (1997) who mentioned high similarity to *U. apsheronica* | 14-18 | freshwater |
| *Urotricha terricola* Alekperov and Musayev, 1988 |  | Redescription required according to Foissner and Pfister (1997) | 16 | soil |
| *Urotricha simonsbergeri* Foissner et al., 1999 |  |  | ca. 25 | freshwater |
| *Urotricha venatrix* (Kahl, 1935) Foissner and Pfister, 1997 |  |  | 27-35 | freshwater |
