# Supplementary material for: Molecular Data Reveal a Cryptic Diversity in the Genus Urotricha (Alveolata, Ciliophora, Prostomatida), a Key Player in Freshwater Lakes, With Remarks on Morphology, Food Preferences, and Distribution
Source: Front Microbiol. 2022 Feb 4;12:787290. doi: 10.3389/fmicb.2021.787290 (PMC8854374; doi:10.3389/fmicb.2021.787290)
Supplement: Supplementary Table 4 — Main morphometric data on the investigated Urotricha strains. Measurements in μm. CV = coefficient of variation in %, M = median, IV = in vivo, Max = maximum, Mean = arithmetic mean, Meth = applied methods, Min = minimum, n = number of specimens investigated, P = after protargol staining, QPS = after quantitative protargol staining, SD = standard deviation. Corresponding strains are color-coordinated. Urotricha agilis (CIL-2017/24, CIL-2019/10), Urotricha furcata (CIL-2019/13 and CIL-2019/6), Urotricha pseudofurcata (CIL-2019/3), and Urotricha castalia (CIL-2017/25, CIL-2017/27, and CIL-2019/1; CIL-48 is the original monoculture of U. castalia of which the clonal strain CIL-2019/1 derived). [file Table_4.DOCX]

| Character | Strain | Meth. | Mean | M | SD | SE | CV | Min | Max | n |
| --- | --- | --- | --- | --- | --- | --- | --- | --- | --- | --- |
| Cell, length | CIL-2017/24 | IV | 17.9 | 18.1 | 2.0 | 0.4 | 10.9 | 14.1 | 22.8 | 21 |
|  |  | P | 13.8 | 13.9 | 1.3 | 0.2 | 9.3 | 10.3 | 16.3 | 33 |
|  | CIL-2019/10 | IV | 16.4 | 16.5 | 1.7 | 0.4 | 10.4 | 13.3 | 19.2 | 21 |
|  |  | P | 11.4 | 11.0 | 1.5 | 0.3 | 13.2 | 8.7 | 14.5 | 21 |
|  | CIL-2019/13 | IV | 22.4 | 21.7 | 3.9 | 0.8 | 17.2 | 16.6 | 35.2 | 21 |
|  | CIL-2019/6 | P | 16.0 | 16.3 | 2.0 | 0.4 | 12.7 | 10.7 | 18.5 | 23 |
|  | CIL-2019/3 | IV | 19.8 | 19.9 | 2.3 | 0.5 | 11.4 | 15.7 | 23.8 | 21 |
|  |  | P | 12.8 | 12.2 | 2.4 | 0.5 | 19.1 | 8.5 | 19.8 | 23 |
|  | CIL-2017/25 | IV | 38.7 | 38.9 | 5.6 | 1.2 | 14.5 | 26.1 | 50.6 | 21 |
|  |  | P | 21.8 | 22.1 | 2.8 | 0.6 | 12.7 | 17.7 | 28.2 | 23 |
|  | CIL-2017/27 | IV | 49.1 | 48.7 | 6.2 | 1.4 | 12.6 | 37.9 | 59.2 | 21 |
|  |  | P | 23.6 | 23.9 | 3.4 | 0.7 | 14.5 | 17.0 | 29.0 | 23 |
|  | CIL-2019/1 | IV | 39.7 | 39.9 | 7.7 | 1.7 | 19.3 | 25.4 | 53.3 | 21 |
|  |  | P | 30.1 | 29.1 | 4.1 | 0.8 | 13.7 | 24.4 | 41.5 | 27 |
| Cell, width | CIL-2017/24 | IV | 11.7 | 11.7 | 1.8 | 0.4 | 15.3 | 8.9 | 16.1 | 21 |
|  |  | P | 10.6 | 10.5 | 1.6 | 0.3 | 14.9 | 8.0 | 13.5 | 33 |
|  | CIL-2019/10 | IV | 11.8 | 11.6 | 1.4 | 0.3 | 12.1 | 9.1 | 14.4 | 21 |
|  |  | P | 8.0 | 8.0 | 1.3 | 0.3 | 16.4 | 6.0 | 11.0 | 21 |
|  | CIL-2019/13 | IV | 14.7 | 14.3 | 3.7 | 0.8 | 25.1 | 10.3 | 28.5 | 21 |
|  | CIL-2019/6 | P | 13.2 | 13.4 | 2.0 | 0.4 | 15.5 | 10.4 | 16.3 | 23 |
|  | CIL-2019/3 | IV | 13.7 | 12.7 | 2.4 | 0.5 | 17.9 | 10.4 | 18.5 | 21 |
|  |  | P | 9.3 | 8.6 | 2.2 | 0.5 | 23.8 | 6.2 | 14.2 | 23 |
|  | CIL-2017/25 | IV | 31.4 | 30.3 | 4.7 | 1.0 | 14.9 | 24.9 | 43.8 | 21 |
|  |  | P | 15.8 | 15.7 | 2.0 | 0.4 | 12.4 | 13.3 | 19.4 | 23 |
|  | CIL-2017/27 | IV | 35.9 | 37.3 | 6.2 | 1.4 | 17.4 | 25.1 | 43.6 | 21 |
|  |  | P | 17.8 | 17.6 | 2.4 | 0.5 | 13.3 | 14.1 | 22.9 | 23 |
|  | CIL-2019/1 | IV | 35.5 | 35.8 | 6.2 | 1.4 | 17.5 | 25.5 | 46.4 | 21 |
|  |  | P | 25.9 | 25.4 | 4.1 | 0.8 | 15.9 | 20.7 | 37.6 | 27 |
| Cell length: width, | CIL-2017/24 | IV | 1.5 | 1.5 | 0.2 | 0.0 | 10.9 | 1.2 | 1.9 | 21 |
| ratio |  | P | 1.3 | 1.3 | 0.2 | 0.0 | 12.0 | 1.0 | 1.6 | 33 |
|  | CIL-2019/10 | IV | 1.4 | 1.4 | 0.2 | 0.0 | 14.1 | 1.1 | 1.9 | 21 |
|  |  | P | 1.5 | 1.4 | 0.2 | 0.0 | 12.6 | 1.1 | 1.8 | 21 |
|  | CIL-2019/13 | IV | 1.5 | 1.5 | 0.2 | 0.0 | 11.1 | 1.2 | 1.9 | 21 |
|  | CIL-2019/6 | P | 1.2 | 1.2 | 0.2 | 0.0 | 15.5 | 0.9 | 1.7 | 23 |
|  | CIL-2019/3 | IV | 1.5 | 1.5 | 0.2 | 0.0 | 11.0 | 1.2 | 1.8 | 21 |
|  |  | P | 1.4 | 1.4 | 0.1 | 0.0 | 10.0 | 1.2 | 1.8 | 23 |
|  | CIL-2017/25 | IV | 1.2 | 1.2 | 0.2 | 0.0 | 14.0 | 0.8 | 1.6 | 21 |
|  |  | P | 1.4 | 1.4 | 0.2 | 0.0 | 12.3 | 1.1 | 1.8 | 23 |
|  | CIL-2017/27 | IV | 1.4 | 1.3 | 0.2 | 0.0 | 12.9 | 1.2 | 1.8 | 21 |
|  |  | P | 1.3 | 1.3 | 0.1 | 0.0 | 10.1 | 1.1 | 1.6 | 23 |
|  | CIL-2019/1 | IV | 1.1 | 1.2 | 0.2 | 0.0 | 17.6 | 0.8 | 1.4 | 21 |
|  |  | P | 1.2 | 1.1 | 0.1 | 0.0 | 7.8 | 1.1 | 1.4 | 27 |
| Distance between circumoral kinety and somatic kineties | CIL-2017/24 | IV | - | - | - | - | - | - | - | - |
|  |  | P | 2.3 | 2.0 | 0.8 | 0.2 | 35.9 | 0.5 | 4.0 | 21 |
|  | CIL-2019/10 | IV | - | - | - | - | - | - | - | - |
|  |  | P | 2.3 | 2.0 | 0.4 | 0.1 | 17.3 | 1.8 | 3.0 | 21 |
|  | CIL-2019/13 | IV | - | - | - | - | - | - | - | - |
|  | CIL-2019/6 | P | 1.0 | 1.0 | 0.5 | 0.1 | 51.6 | 0.0 | 1.5 | 21 |
|  | CIL-2019/3 | IV | - | - | - | - | - | - | - | - |
|  |  | P | 1.2 | 1.0 | 1.3 | 0.1 | 24.7 | 1.0 | 2.0 | 21 |
|  | CIL-2017/25 | IV | - | - | - | - | - | - | - | - |
|  |  | P | 0.6 | 0.5 | 0.4 | 0.1 | 61.0 | 0.1 | 1.0 | 21 |
|  | CIL-2017/27 | IV | - | - | - | - | - | - | - | - |
|  |  | P | 0.8 | 1.0 | 0.3 | 0.1 | 36.0 | 0.3 | 1.0 | 7 |
|  | CIL-2019/1 | IV | - | - | - | - | - | - | - | - |
|  |  | P | 1.0 | 1.0 | 0.3 | 0.1 | 34.8 | 0.5 | 2.0 | 21 |
| Unciliated | CIL-2017/24 | IV | - | - | - | - | - | - | - | - |
| posterior portion, |  | P | 3.5 | 3.4 | 0.8 | 0.2 | 23.1 | 2.5 | 5.7 | 21 |
| length | CIL-2019/10 | IV | - | - | - | - | - | - | - | - |
|  |  | P | 4.2 | 4.0 | 0.6 | 0.1 | 13.3 | 3.0 | 5.0 | 19 |
|  | CIL-2019/13 | IV | - | - | - | - | - | - | - | - |
|  | CIL-2019/6 | P | 3.4 | 3.5 | 0.6 | 0.1 | 18.3 | 2.3 | 4.4 | 21 |
|  | CIL-2019/3 | IV | - | - | - | - | - | - | - | - |
|  |  | P | 3.5 | 3.2 | 1.5 | 0.3 | 42.3 | 1.0 | 9.0 | 21 |
|  | CIL-2017/25 | IV | 10.1 | 9.1 | 2.5 | 0.5 | 24.9 | 6.8 | 15.0 | 21 |
|  |  | P | 4.2 | 4.3 | 0.8 | 0.2 | 18.1 | 3.0 | 5.7 | 21 |
|  | CIL-2017/27 | IV | - | - | - | - | - | - | - | - |
|  |  | P | 4.1 | 3.9 | 1.0 | 0.2 | 25.2 | 2.4 | 6.4 | 21 |
|  | CIL-2019/1 | IV | - | - | - | - | - | - | - | - |
|  |  | P | 6.1 | 6.3 | 1.5 | 0.4 | 24.0 | 3.4 | 9.3 | 11 |
| Caudal cilia, length | CIL-2017/24 | IV | 9.0 | 8.7 | 1.7 | 0.4 | 19.3 | 6.3 | 12.1 | 21 |
|  |  | P | 7.3 | 7.4 | 1.4 | 0.3 | 18.9 | 5.2 | 9.7 | 21 |
|  | CIL-2019/10 | IV | 6.8 | 6.8 | 1.0 | 0.2 | 15.3 | 4.8 | 8.7 | 21 |
|  |  | P | 7.0 | 7.0 | 0.7 | 0.1 | 9.7 | 6.0 | 8.5 | 21 |
|  | CIL-2019/13 | IV | 7.9 | 7.7 | 1.3 | 0.3 | 15.9 | 5.5 | 10.8 | 21 |
|  | CIL-2019/6 | P | 6.0 | 5.9 | 1.1 | 0.2 | 19.1 | 4.0 | 8.0 | 21 |
|  | CIL-2019/3 | IV | 6.4 | 6.3 | 1.1 | 0.2 | 16.5 | 4.8 | 8.8 | 21 |
|  |  | P | 6.1 | 5.7 | 1.2 | 0.3 | 19.9 | 4.5 | 8.7 | 21 |
|  | CIL-2017/25 | IV | 9.4 | 9.3 | 1.1 | 0.2 | 12.0 | 7.3 | 12.6 | 21 |
|  |  | P | 5.9 | 6.0 | 1.1 | 0.2 | 19.4 | 3.1 | 8.5 | 21 |
|  | CIL-2017/27 | IV | 8.4 | 8.6 | 0.9 | 0.2 | 10.7 | 6.7 | 9.8 | 21 |
|  |  | P | 6.4 | 6.7 | 1.1 | 0.2 | 16.6 | 4.6 | 8.3 | 21 |
|  | CIL-2019/1 | IV | 8.0 | 8.3 | 1.5 | 0.3 | 19.2 | 4.3 | 10.7 | 21 |
|  |  | P | 5.9 | 5.1 | 1.6 | 0.3 | 27.3 | 4.4 | 10.6 | 21 |
| Caudal cilia, number | CIL-2017/24 | IV | 1.0 | 1.0 | 0.0 | 0.0 | 0.0 | 1.0 | 1.0 | 21 |
|  |  | P | 1.0 | 1.0 | 0.0 | 0.0 | 0.0 | 1.0 | 1.0 | 21 |
|  | CIL-2019/10 | IV | 1.0 | 1.0 | 0.0 | 0.0 | 0.0 | 1.0 | 1.0 | 21 |
|  |  | P | 1.0 | 1.0 | 0.0 | 0.0 | 0.0 | 1.0 | 1.0 | 21 |
|  | CIL-2019/13 | IV | 2.0 | 2.0 | 0.0 | 0.0 | 0.0 | 2.0 | 2.0 | 21 |
|  | CIL-2019/6 | P | 2.0 | 2.0 | 0.0 | 0.0 | 0.0 | 2.0 | 2.0 | 21 |
|  | CIL-2019/3 | IV | 2.0 | 2.0 | 0.0 | 0.0 | 0.0 | 2.0 | 2.0 | 21 |
|  |  | P | 2.0 | 2.0 | 0.0 | 0.0 | 0.0 | 2.0 | 2.0 | 21 |
|  | CIL-2017/25 | IV | 4.5 | 4.0 | 0.8 | 0.2 | 18.2 | 4.0 | 7.0 | 21 |
|  |  | P | 5.1 | 5.0 | 1.0 | 0.2 | 19.5 | 4.0 | 7.0 | 21 |
|  | CIL-2017/27 | IV | 4.4 | 4.0 | 0.8 | 0.2 | 18.3 | 4.0 | 7.0 | 21 |
|  |  | P | 6.0 | 6.0 | 1.1 | 0.2 | 18.0 | 4.0 | 7.0 | 21 |
|  | CIL-2019/1 | IV | 4.7 | 4.0 | 0.9 | 0.2 | 19.6 | 4.0 | 7.0 | 21 |
|  |  | P | 5.4 | 5.0 | 1.3 | 0.3 | 23.3 | 4.0 | 7.0 | 19 |
| Somatic cilia, length | CIL-2017/24 | IV | 4.5 | 4.6 | 0.8 | 0.2 | 17.7 | 3.0 | 6.2 | 21 |
|  |  | P | 4.4 | 4.3 | 0.9 | 0.2 | 19.9 | 2.9 | 6.5 | 21 |
|  | CIL-2019/10 | IV | 4.1 | 4.2 | 1.0 | 0.2 | 24.7 | 2.1 | 6.3 | 21 |
|  |  | P | 5.5 | 6.0 | 0.8 | 0.2 | 15.1 | 4.0 | 6.5 | 21 |
|  | CIL-2019/13 | IV | 5.6 | 5.9 | 1.0 | 0.2 | 17.1 | 3.2 | 6.8 | 21 |
|  | CIL-2019/6 | P | 4.1 | 4.0 | 0.8 | 0.2 | 19.5 | 2.6 | 5.7 | 23 |
|  | CIL-2019/3 | IV | 4.1 | 4.2 | 0.5 | 0.1 | 13.2 | 2.8 | 5.1 | 21 |
|  |  | P | 4.1 | 4.1 | 0.7 | 0.2 | 17.1 | 3.1 | 6.0 | 21 |
|  | CIL-2017/25 | IV | - | - | - | - | - | - | - | - |
|  |  | P | 3.7 | 3.7 | 0.6 | 0.1 | 16.0 | 2.8 | 5.1 | 21 |
|  | CIL-2017/27 | IV | - | - | - | - | - | - | - | - |
|  |  | P | 3.7 | 3.6 | 0.9 | 0.2 | 24.0 | 2.3 | 5.8 | 21 |
|  | CIL-2019/1 | IV | 5.7 | 5.4 | 1.6 | 0.3 | 27.8 | 3.6 | 8.6 | 21 |
|  |  | P | 3.6 | 3.5 | 0.7 | 0.2 | 19.2 | 2.2 | 4.8 | 21 |
| Somatic kineties, number | CIL-2017/24 | IV | - | - | - | - | - | - | - | - |
|  |  | P | 21.6 | 22.0 | 1.6 | 0.4 | 7.5 | 18.0 | 24.0 | 21 |
|  | CIL-2019/10 | IV | - | - | - | - | - | - | - | - |
|  |  | P | 17.7 | 18.0 | 2.2 | 0.5 | 12.5 | 14.0 | 22.0 | 21 |
|  | CIL-2019/13 | IV | - | - | - | - | - | - | - | - |
|  | CIL-2019/6 | P | 21.7 | 22.0 | 1.7 | 0.4 | 8.0 | 18 | 24 | 21 |
|  | CIL-2019/3 | IV | - | - | - | - | - | - | - | - |
|  |  | P | 19.2 | 18.0 | 2.7 | 0.6 | 14.1 | 14.0 | 24.0 | 21 |
|  | CIL-2017/25 | IV | - | - | - | - | - | - | - | - |
|  |  | P | 35.8 | 35.0 | 2.1 | 0.5 | 5.8 | 34.0 | 42.0 | 21 |
|  | CIL-2017/27 | IV | 36.0 | - | 0.0 | 0.0 | 0.0 | 36.0 | 36.0 | 2 |
|  |  | P | 36.0 | 36.0 | 2.9 | 0.6 | 8.0 | 32.0 | 42.0 | 21 |
|  | CIL-2019/1 | IV | 39.0 | 40.0 | 2.0 | 0.7 | 5.1 | 34.0 | 40.0 | 9 |
|  |  | P | 38.2 | - | 3.3 | 0.7 | 8.8 | 34.0 | 46.0 | 20 |
| Cilia in an anteriorly unshortened somatic kinety, number | CIL-2017/24 | IV | 9.1 | 9.0 | 1.2 | 0.3 | 13.0 | 7.0 | 12.0 | 21 |
|  |  | P | 8.8 | 8.0 | 1.2 | 0.3 | 13.9 | 6.0 | 11.0 | 21 |
|  | CIL-2019/10 | IV | - | - | - | - | - | - | - | - |
|  |  | P | 5.8 | 6.0 | 0.8 | 0.2 | 14.4 | 5.0 | 7.0 | 21 |
|  | CIL-2019/13 | IV | - | - | - | - | - | - | - | - |
|  | CIL-2019/6 | P | 11.4 | 11.0 | 0.9 | 0.2 | 7.9 | 10.0 | 14.0 | 21 |
|  | CIL-2019/3 | IV | - | - | - | - | - | - | - | - |
|  |  | P | 9.6 | 9.0 | 1.7 | 0.4 | 18.0 | 8.0 | 14.0 | 21 |
|  | CIL-2017/25 | IV | - | - | - | - | - | - | - | - |
|  |  | P | 18.0 | 18.0 | 3.2 | 0.7 | 17.7 | 13.0 | 27.0 | 21 |
|  | CIL-2017/27 | IV | - | - | - | - | - | - | - | - |
|  |  | P | 19.0 | 19.0 | 2.8 | 0.6 | 14.9 | 13.0 | 27.0 | 21 |
|  | CIL-2019/1 | IV | - | - | - | - | - | - | - | - |
|  |  | P | 21.2 | - | 3.1 | 0.7 | 14.6 | 18.0 | 30.0 | 18 |
| Adoral organelle1, length | CIL-2017/24 | IV | - | - | - | - | - | - | - | - |
|  |  | P | 2.0 | 2.0 | 0.2 | 0.0 | 11.5 | 1.5 | 2.5 | 21 |
|  | CIL-2019/10 | IV | - | - | - | - | - | - | - | - |
|  |  | P | 2.1 | 2.0 | 0.3 | 0.1 | 12.7 | 2.0 | 3.0 | 21 |
|  | CIL-2019/13 | IV | - | - | - | - | - | - | - | - |
|  | CIL-2019/6 | P | 1.9 | 2.0 | 0.4 | 0.1 | 22.3 | 1.0 | 2.5 | 17 |
|  | CIL-2019/3 | IV | - | - | - | - | - | - | - | - |
|  |  | P | 2.0 | 2.0 | 0.2 | 0.0 | 11.2 | 1.5 | 2.5 | 21 |
|  | CIL-2017/25 | IV | - | - | - | - | - | - | - | - |
|  |  | P | 1.9 | 2.0 | 0.2 | 0.0 | 11.6 | 1.5 | 2.0 | 21 |
|  | CIL-2017/27 | IV | - | - | - | - | - | - | - | - |
|  |  | P | 2.0 | - | 0.3 | 0.1 | 16.5 | 1.2 | 2.5 | 14 |
|  | CIL-2019/1 | IV | - | - | - | - | - | - | - | - |
|  | + CIL-48 | P+QPS | 2.4 | - | 0.4 | 0.1 | 17.8 | 2.0 | 3.0 | 14 |
| Adoral organelle 2, length | CIL-2017/24 | IV | - | - | - | - | - | - | - | - |
|  |  | P | 0.9 | - | 0.1 | 0.0 | 12.2 | 0.8 | 1.2 | 18 |
|  | CIL-2019/10 | IV | - | - | - | - | - | - | - | - |
|  |  | P | 1.0 | 1.0 | 0.0 | 0.0 | 0.0 | 1.0 | 1.0 | 5 |
|  | CIL-2019/13 | IV | - | - | - | - | - | - | - | - |
|  | CIL-2019/6 | P | 1.1 | - | 0.2 | 0.1 | 21.3 | 0.8 | 1.5 | 10 |
|  | CIL-2019/3 | IV | - | - | - | - | - | - | - | - |
|  |  | P | 1.4 | 1.5 | 0.2 | 0.1 | 13.2 | 1.0 | 1.5 | 7 |
|  | CIL-2017/25 | IV | - | - | - | - | - | - | - | - |
|  |  | P | 1.5 | 1.5 | 0.2 | 0.0 | 14.6 | 1.2 | 2.0 | 21 |
|  | CIL-2017/27 | IV | - | - | - | - | - | - | - | - |
|  |  | P | 1.3 | 1.2 | 0.3 | 0.1 | 23.5 | 1.0 | 2.0 | 9 |
|  | CIL-2019/1 | IV | - | - | - | - | - | - | - | - |
|  | + CIL-48 | P+QPS | 1.7 | - | 0.3 | 0.1 | 18.8 | 1.0 | 2.0 | 14 |
| Adoral organelle 3, length | CIL-2017/24 | IV | - | - | - | - | - | - | - | - |
|  |  | P | 0.9 | - | 0.1 | 0.0 | 10.7 | 0.8 | 1.0 | 14 |
|  | CIL-2019/10 | IV | - | - | - | - | - | - | - | - |
|  |  | P | 1.0 | 1.0 | 0.0 | 0.0 | 0.0 | 1.0 | 1.0 | 3 |
|  | CIL-2019/13 | IV | - | - | - | - | - | - | - | - |
|  | CIL-2019/6 | P | 1.0 | - | 0.1 | 0.0 | 8.4 | 0.8 | 1.0 | 6 |
|  | CIL-2019/3 | IV | - | - | - | - | - | - | - | - |
|  |  | P | - | - | - | - | - | 1.5 | 1.5 | 1 |
|  | CIL-2017/25 | IV | - | - | - | - | - | - | - | - |
|  |  | P | 0.9 | 1.0 | 0.2 | 0.0 | 20.9 | 0.2 | 1.2 | 19 |
|  | CIL-2017/27 | IV | - | - | - | - | - | - | - | - |
|  |  | P | 0.9 | - | 0.3 | 0.1 | 28.6 | 0.5 | 1.0 | 4 |
|  | CIL-2019/1 | IV | - | - | - | - | - | - | - | - |
|  | + CIL-48 | P+QPS | 1.0 | - | 0.3 | 0.1 | 31.9 | 0.5 | 1.5 | 14 |
| Dikinetids in adoral organelle 1, number | CIL-2017/24 | IV | - | - | - | - | - | - | - | - |
|  |  | P | 4.1 | 4.0 | 0.4 | 0.1 | 10.7 | 3.0 | 5.0 | 21 |
|  | CIL-2019/10 | IV | - | - | - | - | - | - | - | - |
|  |  | P | 4.0 | 4.0 | 0.2 | 0.0 | 5.4 | 4.0 | 5.0 | 21 |
|  | CIL-2019/13 | IV | - | - | - | - | - | - | - | - |
|  | CIL-2019/6 | P | 3.7 | 4.0 | 0.5 | 0.1 | 13.0 | 3.0 | 4.0 | 19 |
|  | CIL-2019/3 | IV | - | - | - | - | - | - | - | - |
|  |  | P | 4.0 | 4.0 | 0.3 | 0.1 | 7.9 | 3.0 | 5.0 | 21 |
|  | CIL-2017/25 | IV | - | - | - | - | - | - | - | - |
|  |  | P | 4.0 | 4.0 | 0.0 | 0.0 | 0.0 | 4.0 | 4.0 | 21 |
|  | CIL-2017/27 | IV | - | - | - | - | - | - | - | - |
|  |  | P | 4.0 | - | 0.0 | 0.0 | 0.0 | 4.0 | 4.0 | 14 |
|  | CIL-2019/1 | IV | - | - | - | - | - | - | - | - |
|  | + CIL-48 | P+QPS | 4.5 | - | 0.5 | 0.1 | 11.5 | 4.0 | 5.0 | 14 |
| Dikinetids in adoral organelle 2, number | CIL-2017/24 | IV | - | - | - | - | - | - | - | - |
|  |  | P | 2.1 | - | 0.2 | 0.1 | 11.5 | 2.0 | 3.0 | 18 |
|  | CIL-2019/10 | IV | - | - | - | - | - | - | - | - |
|  |  | P | 2.0 | 2.0 | 0.0 | 0.0 | 0.0 | 2.0 | 2.0 | 5 |
|  | CIL-2019/13 | IV | - | - | - | - | - | - | - | - |
|  | CIL-2019/6 | P | 2.4 | - | 0.5 | 0.1 | 21.3 | 2.0 | 3.0 | 12 |
|  | CIL-2019/3 | IV | - | - | - | - | - | - | - | - |
|  |  | P | 2.8 | - | 0.5 | 0.3 | 18.2 | 2.0 | 3.0 | 4 |
|  | CIL-2017/25 | IV | - | - | - | - | - | - | - | - |
|  |  | P | 3.0 | 3.0 | 0.0 | 0.0 | 0.0 | 3.0 | 3.0 | 21 |
|  | CIL-2017/27 | IV | - | - | - | - | - | - | - | - |
|  |  | P | 3.0 | 3.0 | 0.0 | 0.0 | 0.0 | 3.0 | 3.0 | 9 |
|  | CIL-2019/1 | IV | - | - | - | - | - | - | - | - |
|  | + CIL-48 | P+QPS | 3.2 | - | 0.4 | 0.1 | 13.2 | 3.0 | 4.0 | 14 |
| Dikinetids in adoral organelle 3, number | CIL-2017/24 | IV | - | - | - | - | - | - | - | - |
|  |  | P | 1.9 | 2.0 | 0.3 | 0.1 | 13.4 | 1.0 | 2.0 | 15 |
|  | CIL-2019/10 | IV | - | - | - | - | - | - | - | - |
|  |  | P | 2.0 | 2.0 | 0.0 | 0.0 | 0.0 | 2.0 | 2.0 | 3 |
|  | CIL-2019/13 | IV | - | - | - | - | - | - | - | - |
|  | CIL-2019/6 | P | 2.0 | 2.0 | 0.0 | 0.0 | 0.0 | 2.0 | 2.0 | 7 |
|  | CIL-2019/3 | IV | - | - | - | - | - | - | - | - |
|  |  | P | - | - | - | - | - | 3.0 | 3.0 | 1 |
|  | CIL-2017/25 | IV | - | - | - | - | - | - | - | - |
|  |  | P | 2.0 | 2.0 | 0.3 | 0.1 | 16.7 | 1.0 | 3.0 | 19 |
|  | CIL-2017/27 | IV | - | - | - | - | - | - | - | - |
|  |  | P | 2.0 | - | 0.0 | 0.0 | 0.0 | 2.0 | 2.0 | 4 |
|  | CIL-2019/1 | IV | - | - | - | - | - | - | - | - |
|  | + CIL-48 | P+QPS | 2.4 | - | 0.5 | 0.1 | 21.1 | 2.0 | 3.0 | 14 |
| Circumoral dikinetids/oral flaps, number | CIL-2017/24 | IV | - | - | - | - | - | - | - | - |
|  |  | P | 8.2 | 8.0 | 0.5 | 0.1 | 6.2 | 7.0 | 9.0 | 21 |
|  | CIL-2019/10 | IV | - | - | - | - | - | - | - | - |
|  |  | P | 8.9 | 9.0 | 0.8 | 0.2 | 9.0 | 8.0 | 10.0 | 21 |
|  | CIL-2019/13 | IV | - | - | - | - | - | - | - | - |
|  | CIL-2019/6 | P | 10.0 | 10.0 | 1.3 | 0.3 | 13.2 | 7.0 | 12.0 | 21 |
|  | CIL-2019/3 | IV | - | - | - | - | - | - | - | - |
|  |  | P | 10.2 | 10.0 | 1.1 | 0.2 | 11.1 | 8.0 | 12.0 | 21 |
|  | CIL-2017/25 | IV | - | - | - | - | - | - | - | - |
|  |  | P | 17.8 | 18.0 | 1.4 | 0.3 | 7.7 | 15.0 | 21.0 | 21 |
|  | CIL-2017/27 | IV | - | - | - | - | - | - | - | - |
|  |  | P | 16.6 | 17.0 | 1.3 | 0.3 | 7.7 | 15.0 | 19.0 | 21 |
|  | CIL-2019/1 | IV | - | - | - | - | - | - | - | - |
|  |  | P | 23.0 | 22.0 | 2.7 | 0.6 | 12.0 | 19.0 | 29.0 | 21 |
| Oral flaps, length | CIL-2017/24 | IV | - | - | - | - | - | - | - | - |
|  |  | P | 1.7 | 1.7 | 0.6 | 0.1 | 32.0 | 1.2 | 3.8 | 21 |
|  | CIL-2019/10 | IV | - | - | - | - | - | - | - | - |
|  |  | P | 1.4 | 1.3 | 0.2 | 0.1 | 16.8 | 1.2 | 2.1 | 21 |
|  | CIL-2019/13 | IV | - | - | - | - | - | - | - | - |
|  | CIL-2019/6 | P | 2.0 | 2.1 | 0.4 | 0.1 | 21.8 | 1.2 | 2.9 | 23 |
|  | CIL-2019/3 | IV | - | - | - | - | - | - | - | - |
|  |  | P | 2.0 | 1.8 | 0.7 | 0.1 | 33.0 | 0.9 | 3.3 | 21 |
|  | CIL-2017/25 | IV | - | - | - | - | - | - | - | - |
|  |  | P | 2.2 | 2.2 | 0.3 | 0.1 | 14.6 | 1.6 | 2.8 | 21 |
|  | CIL-2017/27 | IV | - | - | - | - | - | - | - | - |
|  |  | P | 2.0 | 1.9 | 0.3 | 0.1 | 14.2 | 1.5 | 2.7 | 21 |
|  | CIL-2019/1 | IV | - | - | - | - | - | - | - | - |
|  |  | P | 2.6 | 2.4 | 0.6 | 0.1 | 21.3 | 1.8 | 3.9 | 21 |
| Macronucleus, length | CIL-2017/24 | IV | 4.3 | 4.3 | 1.1 | 0.2 | 25.0 | 2.7 | 7.7 | 21 |
|  |  | P | 4.2 | 3.8 | 1.0 | 0.2 | 24.0 | 2.4 | 6.2 | 21 |
|  | CIL-2019/10 | IV | 3.9 | 3.8 | 1.0 | 0.2 | 25.9 | 1.8 | 6.3 | 21 |
|  |  | P | 2.9 | 3.0 | 0.8 | 0.2 | 28.8 | 1.5 | 5.0 | 21 |
|  | CIL-2019/13 | IV | 5.0 | 4.8 | 0.8 | 0.2 | 15.4 | 4.0 | 6.7 | 21 |
|  | CIL-2019/6 | P | 5.3 | 5.4 | 0.9 | 0.2 | 17.9 | 3.4 | 6.6 | 21 |
|  | CIL-2019/3 | IV | 4.6 | 4.6 | 1.0 | 0.2 | 21.5 | 2.7 | 6.9 | 21 |
|  |  | P | 5.0 | 4.8 | 1.0 | 0.3 | 19.9 | 3.7 | 7.2 | 9 |
|  | CIL-2017/25 | IV | 10.8 | 10.6 | 1.4 | 0.3 | 13.4 | 8.5 | 15.1 | 21 |
|  |  | P | 8.0 | 8.0 | 1.6 | 0.3 | 19.9 | 5.3 | 10.9 | 21 |
|  | CIL-2017/27 | IV | 10.4 | 9.7 | 1.4 | 0.3 | 13.1 | 8.8 | 13.3 | 21 |
|  |  | P | 7.8 | 7.2 | 1.7 | 0.4 | 21.8 | 5.3 | 12.1 | 21 |
|  | CIL-2019/1 | IV | 10.0 | 9.9 | 2.2 | 0.5 | 21.4 | 6.6 | 14.9 | 21 |
|  |  | P | 9.1 | - | 1.8 | 0.4 | 20.3 | 6.5 | 13.6 | 24 |
| Macronucleus, width | CIL-2017/24 | IV | 4.0 | 3.9 | 0.7 | 0.1 | 16.6 | 2.8 | 5.2 | 21 |
|  |  | P | 4.1 | 4.0 | 1.0 | 0.2 | 23.8 | 2.7 | 5.8 | 21 |
|  | CIL-2019/10 | IV | 4.1 | 4.2 | 0.9 | 0.2 | 22.7 | 1.8 | 6.2 | 21 |
|  |  | P | 3.2 | 3.0 | 1.0 | 0.2 | 32.2 | 1.0 | 5.0 | 21 |
|  | CIL-2019/13 | IV | 4.9 | 4.6 | 1.0 | 0.2 | 21.1 | 3.3 | 7.2 | 21 |
|  | CIL-2019/6 | P | 4.9 | 4.5 | 1.4 | 0.3 | 27.7 | 3.3 | 9.0 | 21 |
|  | CIL-2019/3 | IV | 4.5 | 4.5 | 1.1 | 0.2 | 24.4 | 2.5 | 6.8 | 21 |
|  |  | P | 4.7 | 4.5 | 0.8 | 0.3 | 16.5 | 3.6 | 6.0 | 9 |
|  | CIL-2017/25 | IV | 8.9 | 8.7 | 1.4 | 0.3 | 15.6 | 7.3 | 12.5 | 21 |
|  |  | P | 7.1 | 7.1 | 1.2 | 0.3 | 17.0 | 5.6 | 10.3 | 21 |
|  | CIL-2017/27 | IV | 10.7 | 10.8 | 1.9 | 0.4 | 18.1 | 8.2 | 13.4 | 21 |
|  |  | P | 7.5 | 7.4 | 1.7 | 0.4 | 22.9 | 5.2 | 12.1 | 21 |
|  | CIL-2019/1 | IV | 9.5 | 9.7 | 1.2 | 0.3 | 13.1 | 6.9 | 12.1 | 21 |
|  |  | P | 8.4 | - | 1.8 | 0.4 | 21.0 | 5.1 | 12.6 | 24 |
| Anterior cell end to macronucleus, distance | CIL-2017/24 | IV | - | - | - | - | - | - | - | - |
|  |  | P | 10.0 | 10.5 | 2.8 | 0.6 | 27.8 | 0.7 | 13.9 | 21 |
|  | CIL-2019/10 | IV | - | - | - | - | - | - | - | - |
|  |  | P | 3.4 | 3.0 | 1.0 | 0.2 | 28.4 | 2.0 | 6.0 | 21 |
|  | CIL-2019/13 | IV | - | - | - | - | - | - | - | - |
|  | CIL-2019/6 | P | 6.3 | 6.0 | 2.3 | 0.5 | 35.8 | 2.0 | 11.0 | 21 |
|  | CIL-2019/3 | IV | - | - | - | - | - | - | - | - |
|  |  | P | 4.4 | 5.0 | 1.0 | 0.2 | 23.4 | 3.0 | 7.0 | 21 |
|  | CIL-2017/25 | IV | - | - | - | - | - | - | - | - |
|  |  | P | 8.5 | 8.0 | 3.4 | 0.7 | 40.1 | 5.0 | 20.0 | 21 |
|  | CIL-2017/27 | IV | - | - | - | - | - | - | - | - |
|  |  | P | 8.8 | 8.0 | 3.2 | 0.7 | 35.9 | 4.0 | 15.0 | 21 |
|  | CIL-2019/1 | IV | - | - | - | - | - | - | - | - |
|  |  | P | 9.4 | 9.0 | 4.3 | 0.9 | 45.5 | 4.0 | 19.0 | 21 |
| Micronucleus, length | CIL-2017/24 | IV | - | - | - | - | - | - | - | - |
|  |  | P | - | - | - | - | - | - | - | - |
|  | CIL-2019/10 | IV | - | - | - | - | - | - | - | - |
|  |  | P | - | - | - | - | - | - | - | - |
|  | CIL-2019/13 | IV | 2.2 | 2.2 | 0.3 | 0.1 | 13.2 | 1.8 | 2.8 | 21 |
|  | CIL-2019/6 | P | - | - | - | - | - | - | - | - |
|  | CIL-2019/3 | IV | 1.7 | 1.7 | 0.3 | 0.1 | 19.0 | 0.9 | 2.3 | 21 |
|  |  | P | - | - | - | - | - | - | - | - |
|  | CIL-2017/25 | IV | - | - | - | - | - | - | - | - |
|  |  | P | - | - | - | - | - | - | - | - |
|  | CIL-2017/27 | IV | 3.0 | - | 1.3 | 0.9 | 44.6 | 2.0 | 3.9 | 2 |
|  |  | P | 2.6 | 2.5 | 0.5 | 0.1 | 18.9 | 2.0 | 3.5 | 21 |
|  | CIL-2019/1 | IV | 2.7 | 2.5 | 0.8 | 0.2 | 29.8 | 1.5 | 4.7 | 21 |
|  |  | P | - | - | - | - | - | 3.0 | 3.0 | 1 |
| Micronucleus, width | CIL-2017/24 | IV | - | - | - | - | - | - | - | - |
|  |  | P | - | - | - | - | - | - | - | - |
|  | CIL-2019/10 | IV | - | - | - | - | - | - | - | - |
|  |  | P | - | - | - | - | - | - | - | - |
|  | CIL-2019/13 | IV | 2.2 | 2.1 | 0.3 | 0.1 | 13.6 | 1.6 | 2.7 | 21 |
|  | CIL-2019/6 | P | - | - | - | - | - | - | - | - |
|  | CIL-2019/3 | IV | 1.7 | 1.7 | 0.3 | 0.1 | 19.7 | 1.1 | 2.4 | 21 |
|  |  | P | - | - | - | - | - | - | - | - |
|  | CIL-2017/25 | IV | - | - | - | - | - | - | - | - |
|  |  | P | - | - | - | - | - | - | - | - |
|  | CIL-2017/27 | IV | 2.6 | - | 0.6 | 0.5 | 25.0 | 2.1 | 3.0 | 2 |
|  |  | P | 2.0 | 2.0 | 0.4 | 0.1 | 19.9 | 1.5 | 3.0 | 21 |
|  | CIL-2019/1 | IV | 2.6 | 2.6 | 0.7 | 0.2 | 28.2 | 1.4 | 4.5 | 21 |
|  |  | P | - | - | - | - | - | 2.0 | 2.0 | 1 |
| Oral basket, distal diameter | CIL-2017/24 | IV | 4.0 | 3.8 | 1.2 | 0.3 | 29.4 | 2.2 | 6.9 | 21 |
|  |  | P | 3.6 | 3.5 | 0.5 | 0.1 | 14.9 | 2.7 | 4.9 | 21 |
|  | CIL-2019/10 | IV | - | - | - | - | - | - | - | - |
|  |  | P | 3.6 | 3.5 | 0.7 | 0.2 | 19.5 | 2.5 | 5.0 | 19 |
|  | CIL-2019/13 | IV | - | - | - | - | - | - | - | - |
|  | CIL-2019/6 | P | 4.2 | 4.1 | 0.6 | 0.1 | 14.5 | 3.2 | 5.7 | 21 |
|  | CIL-2019/3 | IV |  |  |  |  |  |  |  |  |
|  |  | P | 4.1 | 4.0 | 0.6 | 0.1 | 14.4 | 3.0 | 5.2 | 23 |
|  | CIL-2017/25 | IV | - | - | - | - | - | - | - | - |
|  |  | P | 4.6 | 4.8 | 0.6 | 0.1 | 13.0 | 3.6 | 5.8 | 21 |
|  | CIL-2017/27 | IV | - | - | - | - | - | - | - | - |
|  |  | P | 4.7 | 4.8 | 0.7 | 0.1 | 13.9 | 3.4 | 6.0 | 21 |
|  | CIL-2019/1 | IV | 12.7 | 11.7 | 3.9 | 0.8 | 30.4 | 5.7 | 18.7 | 21 |
|  |  | P | 6.8 | - | 1.3 | 0.3 | 18.8 | 4.3 | 10.1 | 24 |
| Oral basket, length | CIL-2017/24 | IV | 3.6 | 3.5 | 1.2 | 0.3 | 32.4 | 2.2 | 6.4 | 21 |
|  |  | P | 8.4 | 8.0 | 1.5 | 0.3 | 17.5 | 5.9 | 11.5 | 21 |
|  | CIL-2019/10 | IV | - | - | - | - | - | - | - | - |
|  |  | P | - | - | - | - | - | - | - | - |
|  | CIL-2019/13 | IV | - | - | - | - | - | - | - | - |
|  | CIL-2019/6 | P | 5.5 | 5.1 | 1.5 | 0.3 | 27.4 | 3.9 | 10.1 | 21 |
|  | CIL-2019/3 | IV |  |  |  |  |  |  |  |  |
|  |  | P | 8.4 | 9.0 | 2.5 | 0.8 | 30.2 | 3.9 | 12.0 | 21 |
|  | CIL-2017/25 | IV | - | - | - | - | - | - | - | - |
|  |  | P | 10.1 | 9.5 | 2.2 | 0.5 | 21.8 | 6.8 | 15.1 | 21 |
|  | CIL-2017/27 | IV | - | - | - | - | - | - | - | - |
|  |  | P | 10.3 | 9.5 | 2.4 | 0.5 | 23.0 | 7.2 | 17.6 | 21 |
|  | CIL-2019/1 | IV | 14.3 | 13.3 | 3.9 | 0.8 | 27.1 | 5.6 | 23.0 | 21 |
|  |  | P | 11.9 | - | 2.0 | 0.4 | 17.1 | 8.3 | 16.2 | 24 |
| Oral basket width:cell width, in % | CIL-2017/24 | IV | 0.3 | 0.4 | 0.1 | 0.0 | 30.4 | 0.2 | 0.6 | 21 |
|  |  | P | 0.3 | 0.4 | 0.1 | 0.0 | 19.1 | 0.2 | 0.4 | 21 |
|  | CIL-2019/10 | IV | 0.3 | 0.3 | 0.1 | 0.0 | 20.2 | 0.2 | 0.3 | 3 |
|  |  | P | 0.5 | - | 0.1 | 0.0 | 22.0 | 0.3 | 0.7 | 20 |
|  | CIL-2019/13 | IV | - | - | - | - | - | - | - | - |
|  | CIL-2019/6 | P | 0.3 | 0.3 | 0.0 | 0.0 | 14.0 | 0.2 | 0.4 | 21 |
|  | CIL-2019/3 | IV | - | - | - | - | - | - | - | - |
|  |  | P | 0.4 | 0.4 | 0.1 | 0.0 | 26.8 | 0.3 | 0.8 | 21 |
|  | CIL-2017/25 | IV | - | - | - | - | - | - | - | - |
|  |  | P | 0.3 | 0.3 | 0.0 | 0.0 | 13.6 | 0.2 | 0.4 | 21 |
|  | CIL-2017/27 | IV | - | - | - | - | - | - | - | - |
|  |  | P | 0.3 | 0.3 | 0.0 | 0.0 | 13.8 | 0.2 | 0.3 | 21 |
|  | CIL-2019/1 | IV | 0.4 | 0.4 | 0.1 | 0.0 | 32.9 | 0.2 | 0.6 | 21 |
|  |  | P | 0.3 | 0.3 | 0.0 | 0.0 | 14.4 | 0.2 | 0.3 | 21 |
| Somatic extrusomes, type 1, length | CIL-2017/24 | IV | - | - | - | - | - | 1.4 | 1.4 | 1 |
|  |  | P | - | - | - | - | - | - | - | - |
|  | CIL-2019/10 | IV | - | - | - | - | - | 1.5 | 1.5 | 1 |
|  |  | P | 1.1 | - | 0.2 | 0.0 | 16.1 | 1.0 | 1.5 | 16 |
|  | CIL-2019/13 | IV | - | - | - | - | - | - | - | - |
|  | CIL-2019/6 | P | - | - | - | - | - | - | - | - |
|  | CIL-2019/3 | IV | - | - | - | - | - | - | - | - |
|  |  | P | 0.9 | 0.8 | 0.2 | 0.0 | 25.2 | 0.6 | 1.5 | 21 |
|  | CIL-2017/25 | IV | 2.3 | 2.2 | 0.4 | 0.1 | 17.0 | 1.7 | 2.9 | 21 |
|  |  | P | - | - | - | - | - | - | - | - |
|  | CIL-2017/27 | IV | 2.4 | 2.6 | 0.5 | 0.1 | 19.0 | 0.8 | 2.9 | 21 |
|  |  | P | - | - | - | - | - | - | - | - |
|  | CIL-2019/1 | IV | 1.8 | 1.8 | 0.2 | 0.0 | 12.1 | 1.3 | 2.2 | 21 |
|  |  | P | - | - | - | - | - | - | - | - |
| Extrusomes type 2, restricted to unciliated posterior cell portion, length | CIL-2017/25 | IV | 4.9 | 4.8 | 0.6 | 0.1 | 12.7 | 4.0 | 6.2 | 18 |
|  |  | P | - | - | - | - | - | - | - | - |
|  | CIL-2017/27 | IV | 4.4 | - | 0.7 | 0.2 | 15.2 | 3.4 | 5.4 | 18 |
|  |  | P | - | - | - | - | - | - | - | - |
|  | CIL-2019/1 | IV | 4.1 | 4.1 | 0.7 | 0.1 | 16.4 | 3.0 | 5.3 | 21 |
|  |  | P | - | - | - | - | - | - | - | - |
