# Supplementary material for: Molecular Data Reveal a Cryptic Diversity in the Genus Urotricha (Alveolata, Ciliophora, Prostomatida), a Key Player in Freshwater Lakes, With Remarks on Morphology, Food Preferences, and Distribution
Source: Front Microbiol. 2022 Feb 4;12:787290. doi: 10.3389/fmicb.2021.787290 (PMC8854374; doi:10.3389/fmicb.2021.787290)
Supplement: Supplementary file 16 [file Data_Sheet_1.pdf]

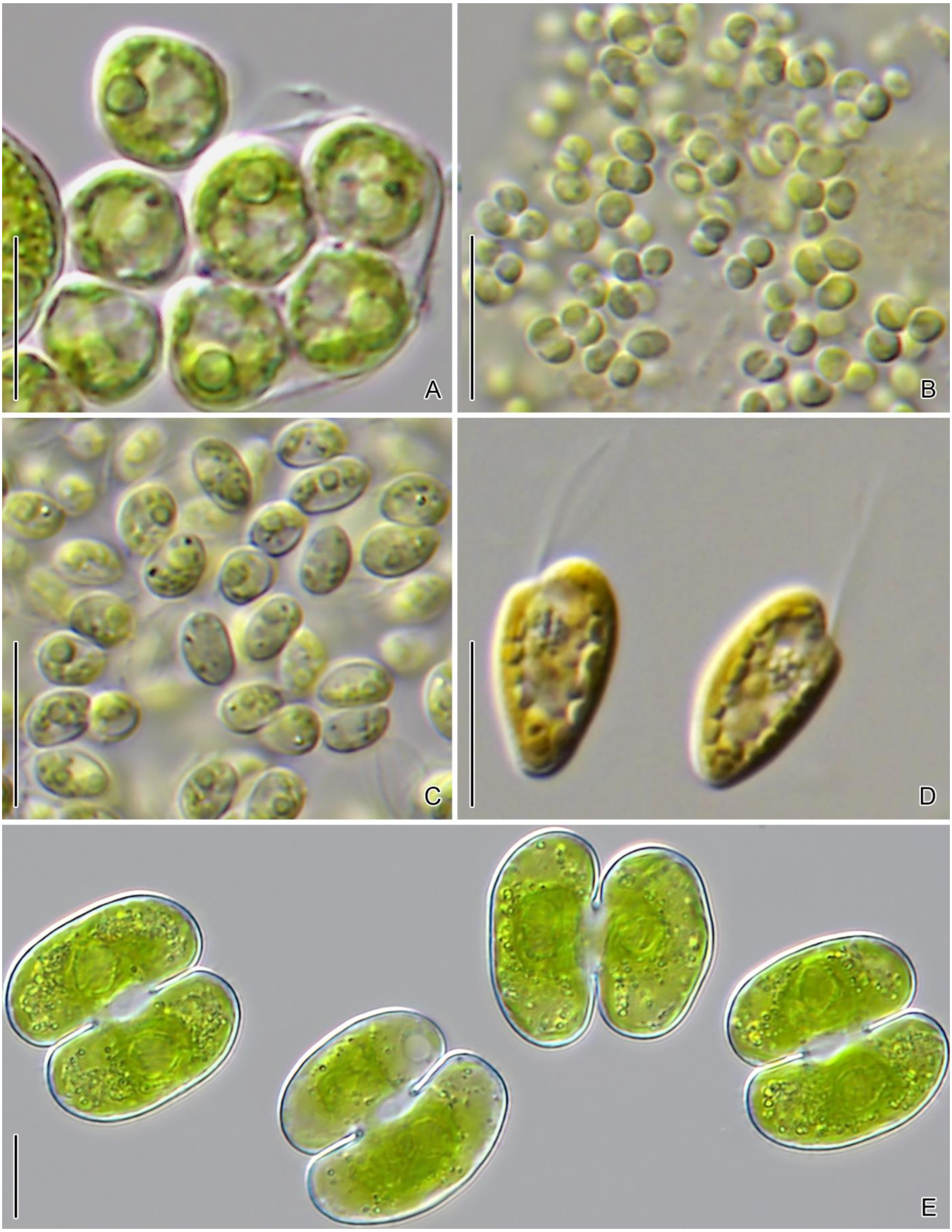

Supplementary Figure 1 | Algal strains used for feeding experiments with *U. castalia* (strain CIL-2017/25): (**A**) MS-2017/1 *Coelastrum* sp., (**B**) MS-2017/2 *Choricystis* sp., (**C**) MS-2017/7 *Acutodesmus obliquus*, (**D**) SAG 26.80 *Cryptomonas* sp., and (**E**) MS-2018/1 *Cosmarium* sp.; scale bar = 10 mm.

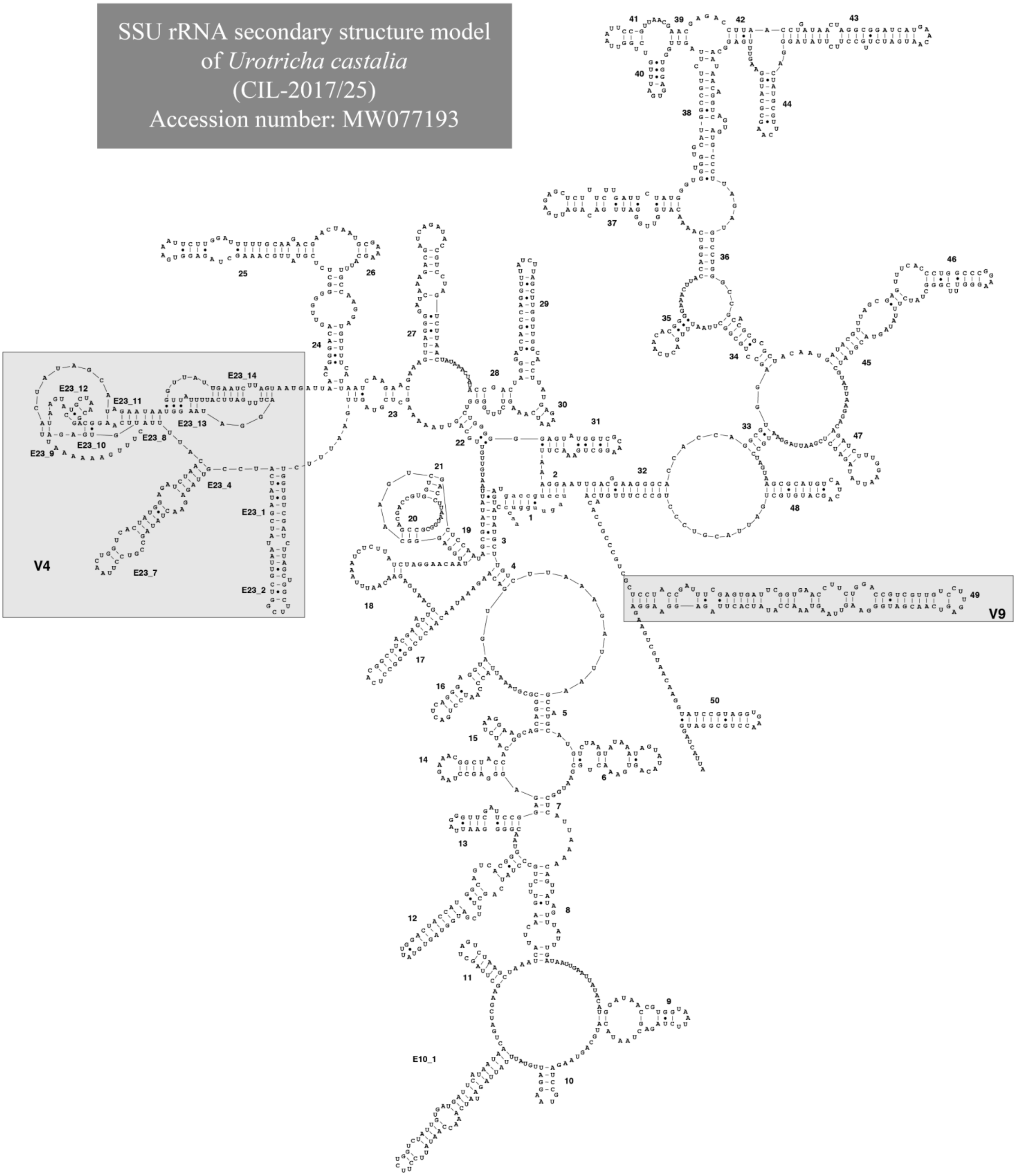

Supplementary Figure 2 | Secondary structure of the SSU rRNA of *Urotricha castalia* (strain CIL-2017/25). The variable regions V4 and V9 are highlighted by gray boxes.

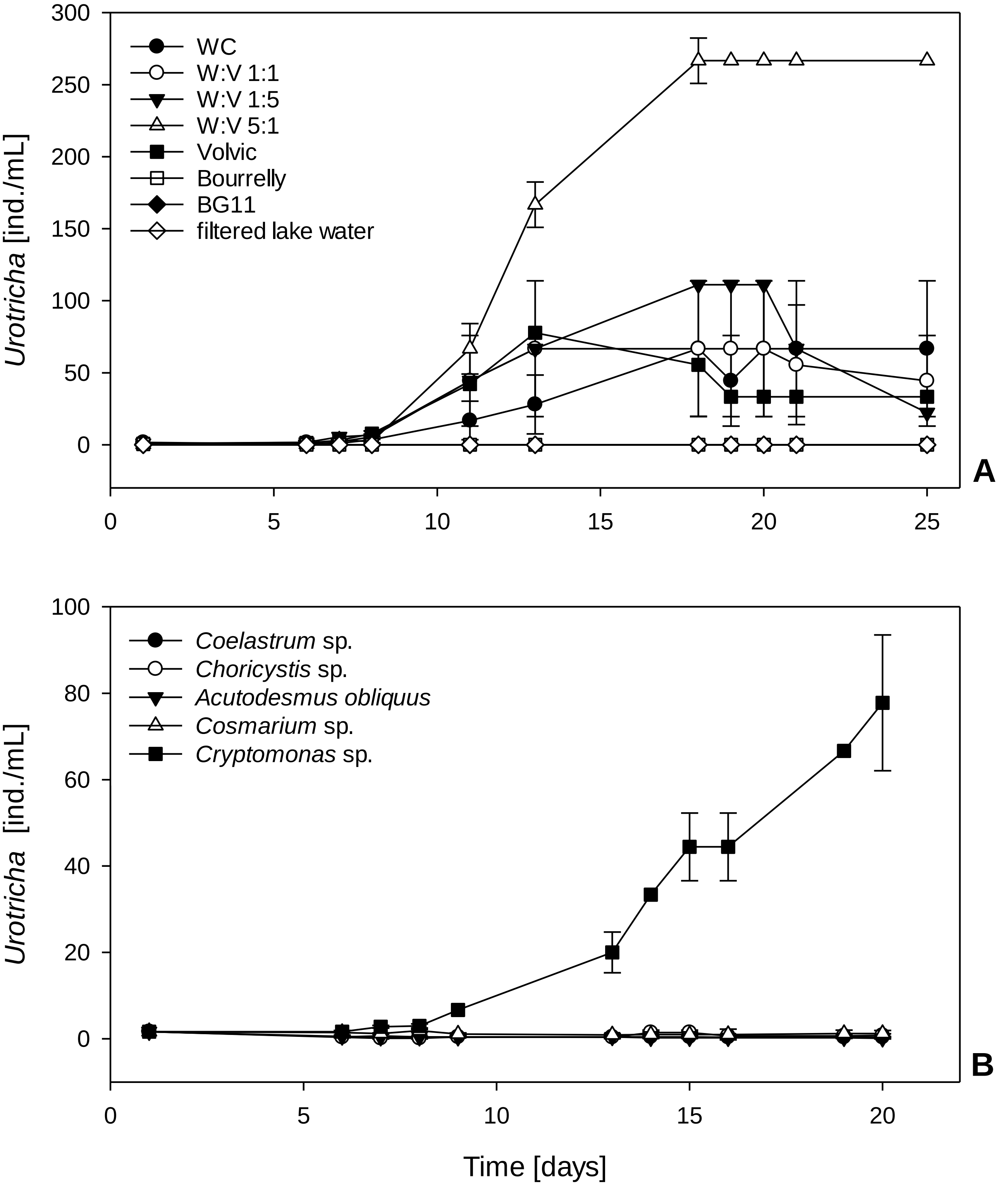

Supplementary Figure 3 | Survival experiments with *Urotricha castalia* (strain CIL-2017/25) from Lake Mondsee, Austria. (**A**) Test of eight media plus SAG 26.80 *Cryptomonas* sp. as food source: Woods Hole MBL medium (WC), mixtures of WC:Volvic® mineral water (V) in 1:1, 1:5, 5:1 v/v; Volvic® mineral water only; modified Bourrelly medium; modified blue-green medium (BG11); sterile-filtered original lake water. (**B**) Test of five algal strains as food source in WC:V 5:1 v/v: MS-2017/1 *Coelastrum* sp., MS-2017/2 *Choricystis* sp., MS-2017/7 *Acutodesmus obliquus*, MS-2017/8 *Cosmarium* sp., and SAG 26.80 *Cryptomonas* sp.

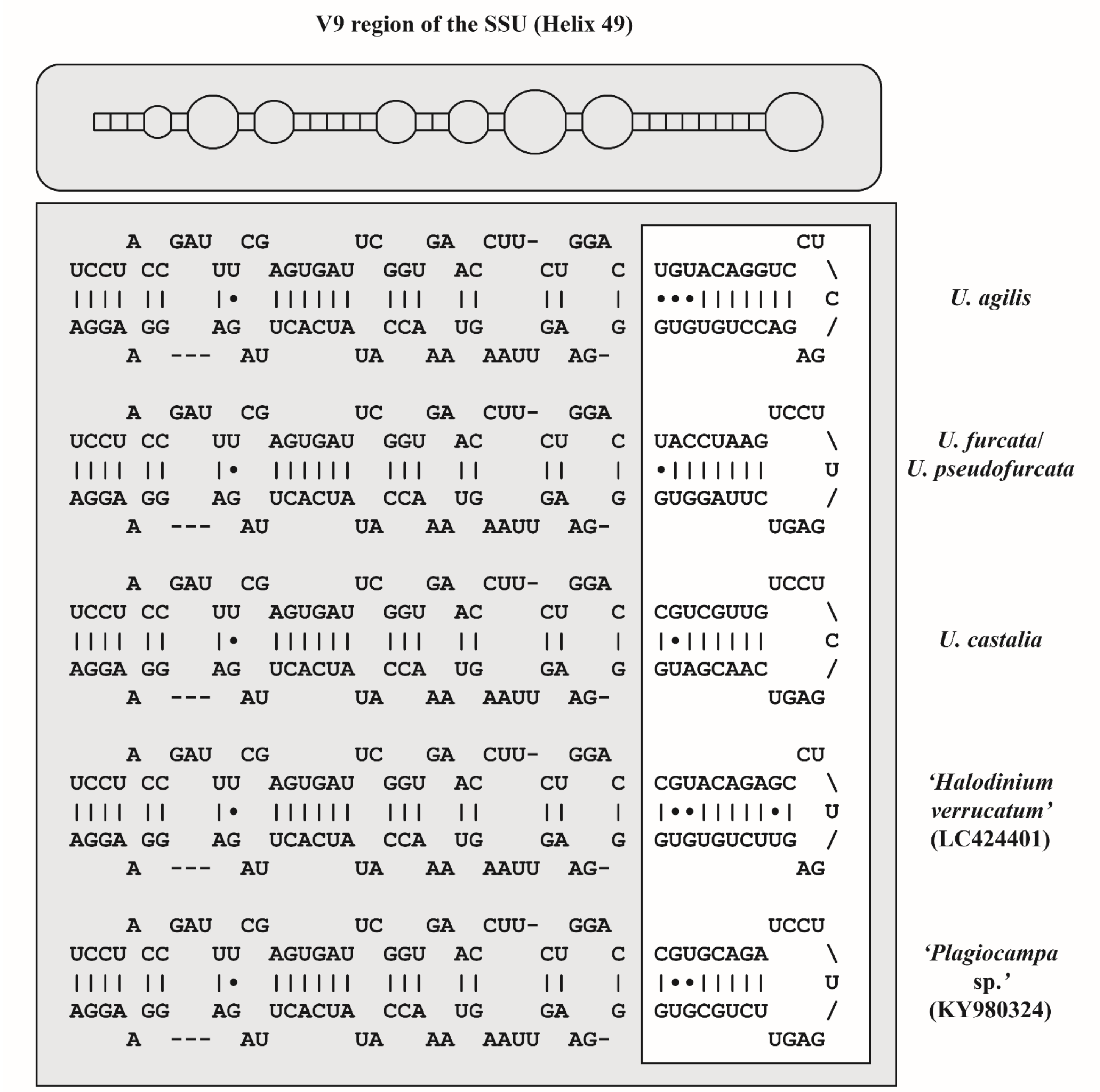

Supplementary Figure 4 | V9 secondary structures of the investigated *Urotricha* strains. The variable regions are marked by white boxes. The structures were calculated with mfold. The line graphic was drawn with PseudoViewer.

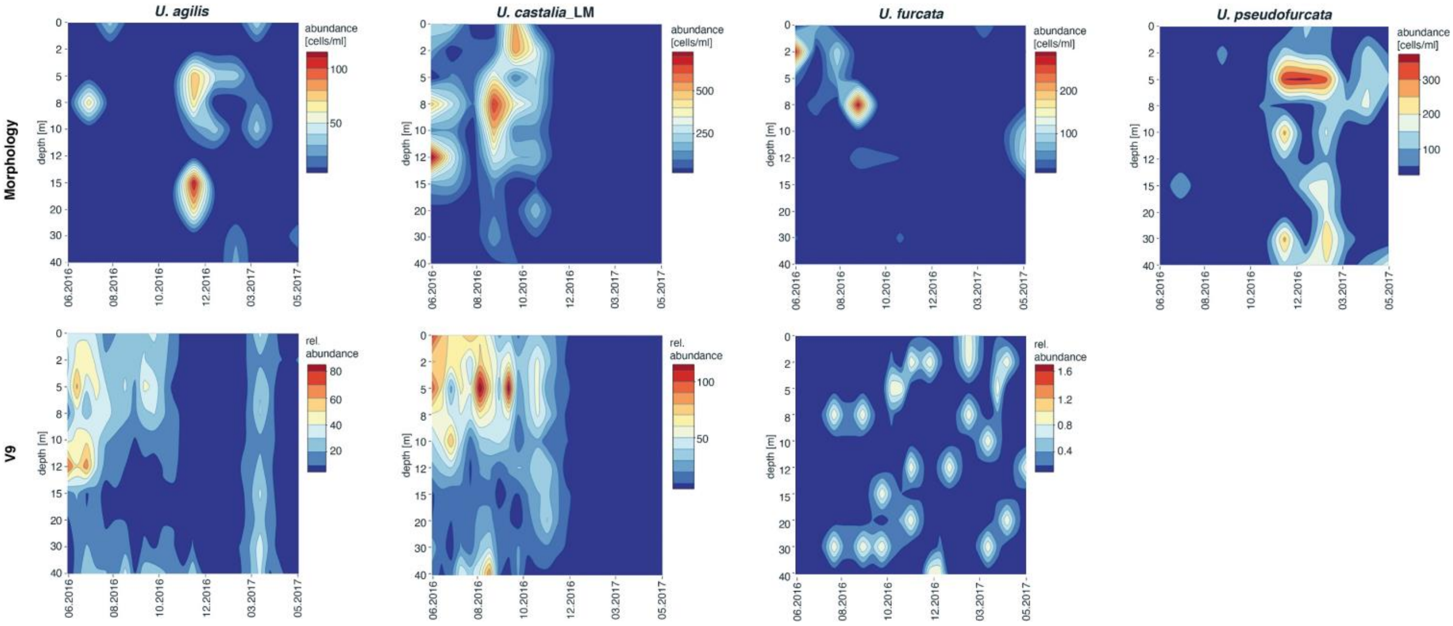

Supplementary Figure 5 | Comparison of temporal and spatial distribution of *Urotricha* strains in Lake Mondsee between sequence data (V9 region) and morphology-based count data. Using the morphology-based approach, *U. agilis*, *U. castalia*, *U. furcata*, and *U. pseudofurcata* could be identified and counted. From the V9 dataset, only V9 markers from *U. agilis*, *U. castalia*_LM, and *U. furcata*/*U. pseudofurcata* could be extracted and used for the comparison.

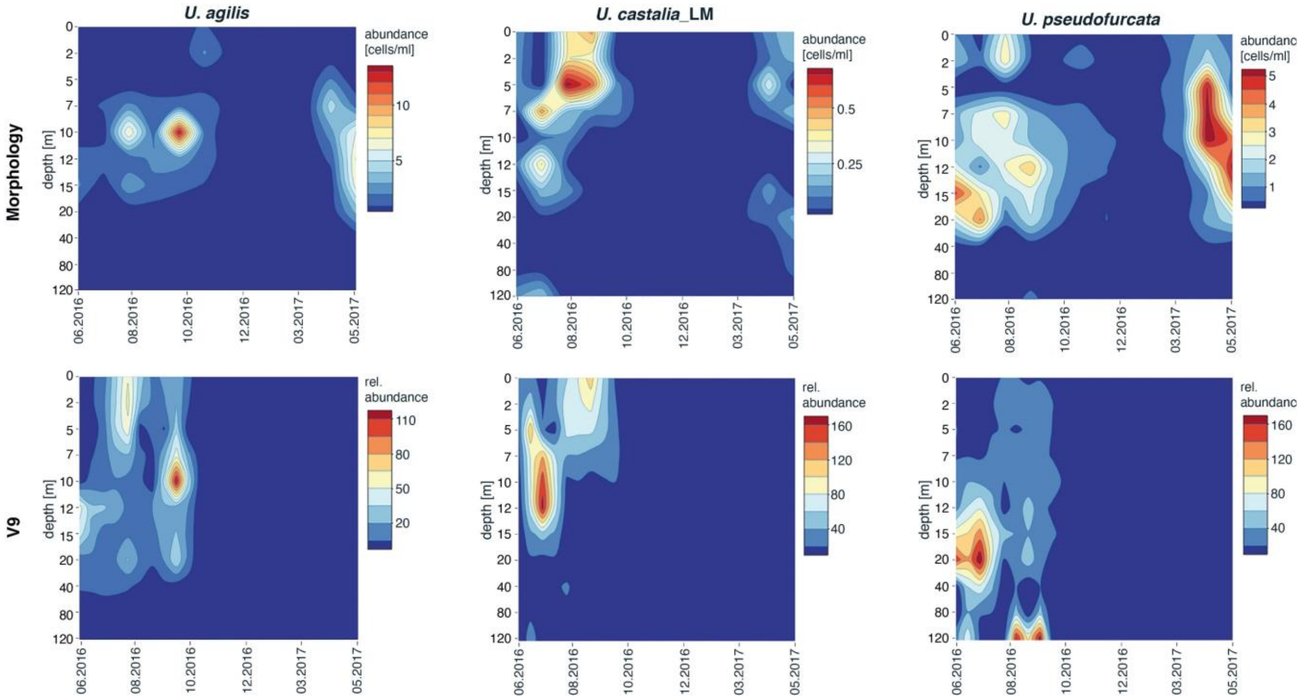

Supplementary Figure 6 | Comparison of temporal and spatial distribution of *Urotricha* strains in Lake Zurich between sequence data (V9 region) and morphology-based count data. Using the morphology-based approach, *U. agilis*, *U. castalia* and *U. pseudofurcata* could be identified and counted. From the V9 dataset, only V9 markers from *U. agilis*, *U. castalia*_LM, and *U. furcata*/*U. pseudofurcata* could be extracted and used for the comparison.

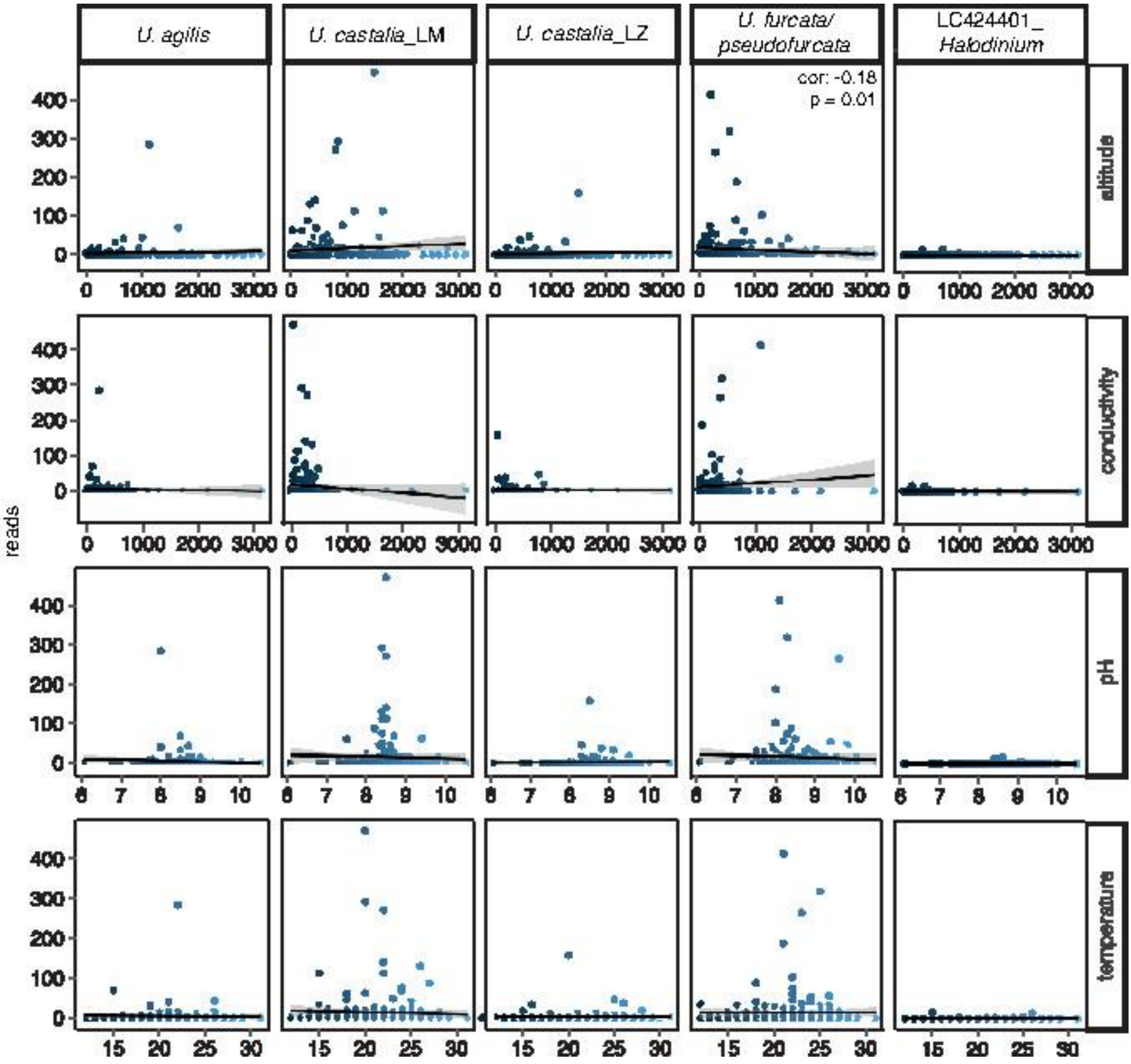

U. agilis
U. castalia_LM
U. castalia_LZ
U. furcata/ pseudofurcata
LC424401_ Halodinium
cor: -0.18
p = 0.01
altitude
conductivity
pH
temperature
reads

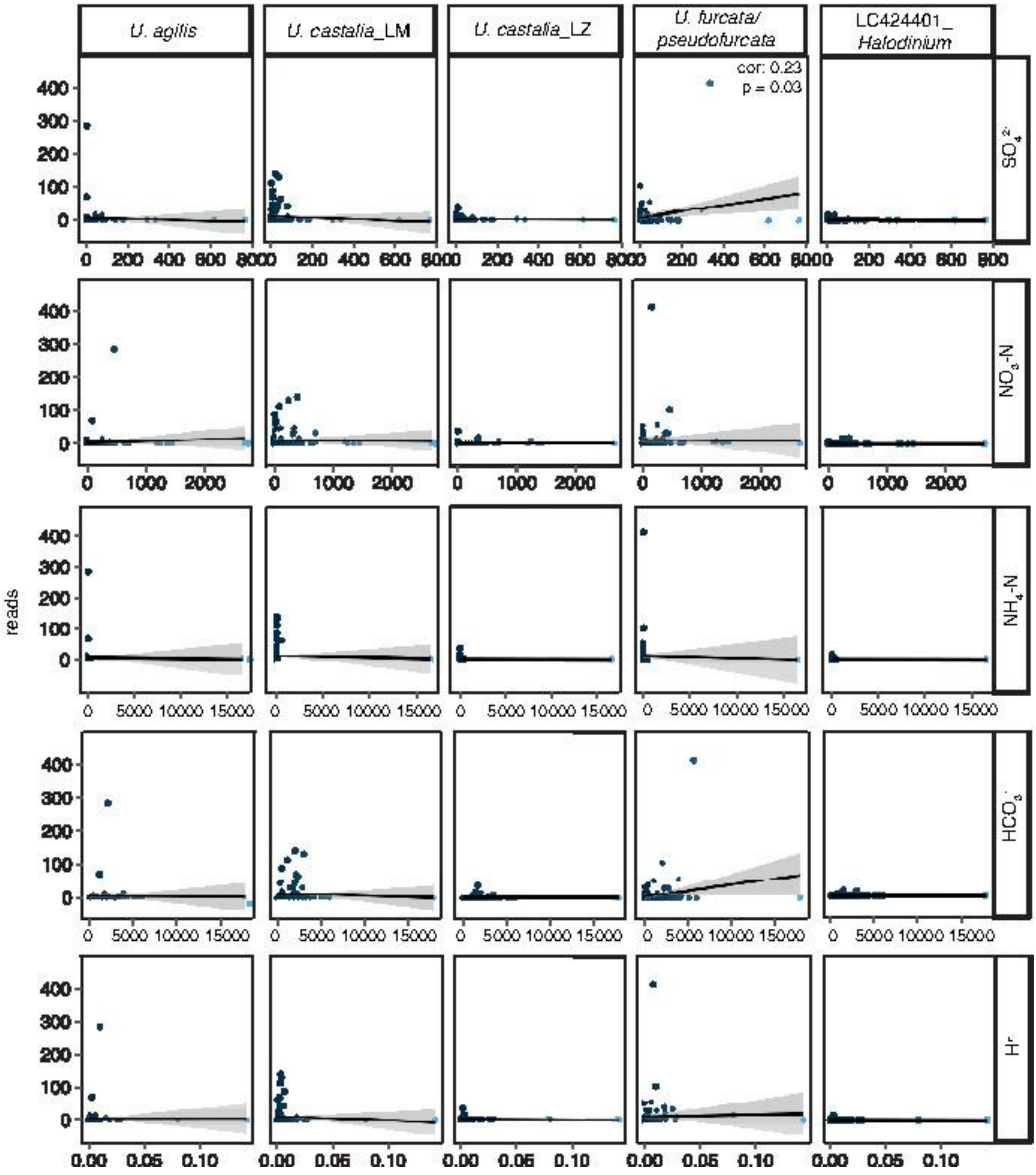

U. agilis
U. castalia_LM
U. castalia_LZ
U. furcata/ pseudofurcata
LC424401_ Halodinium
cor: 0.23
p = 0.03
SO_4^{2-}
NO_3-N
NH_4-N
HCO_3^-
H^+
reads

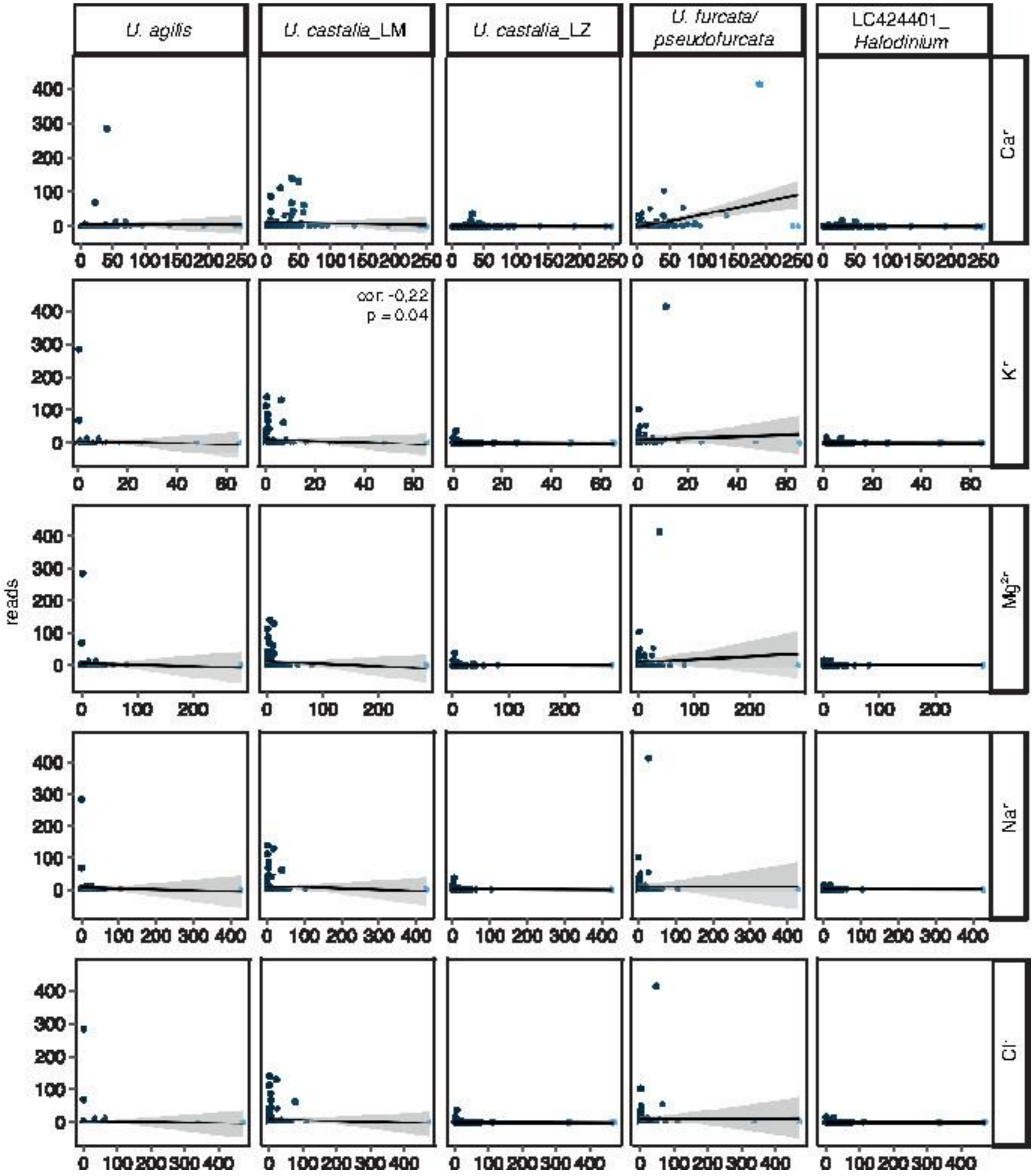

U. agilis
U. castalia_LM
U. castalia_LZ
U. furcata/ pseudofurcata
LC424401_ Halodinium
cor: -0,22
p = 0.04
reads
Ca⁺
K⁺
Mg²⁺
Na⁺
Cl⁻

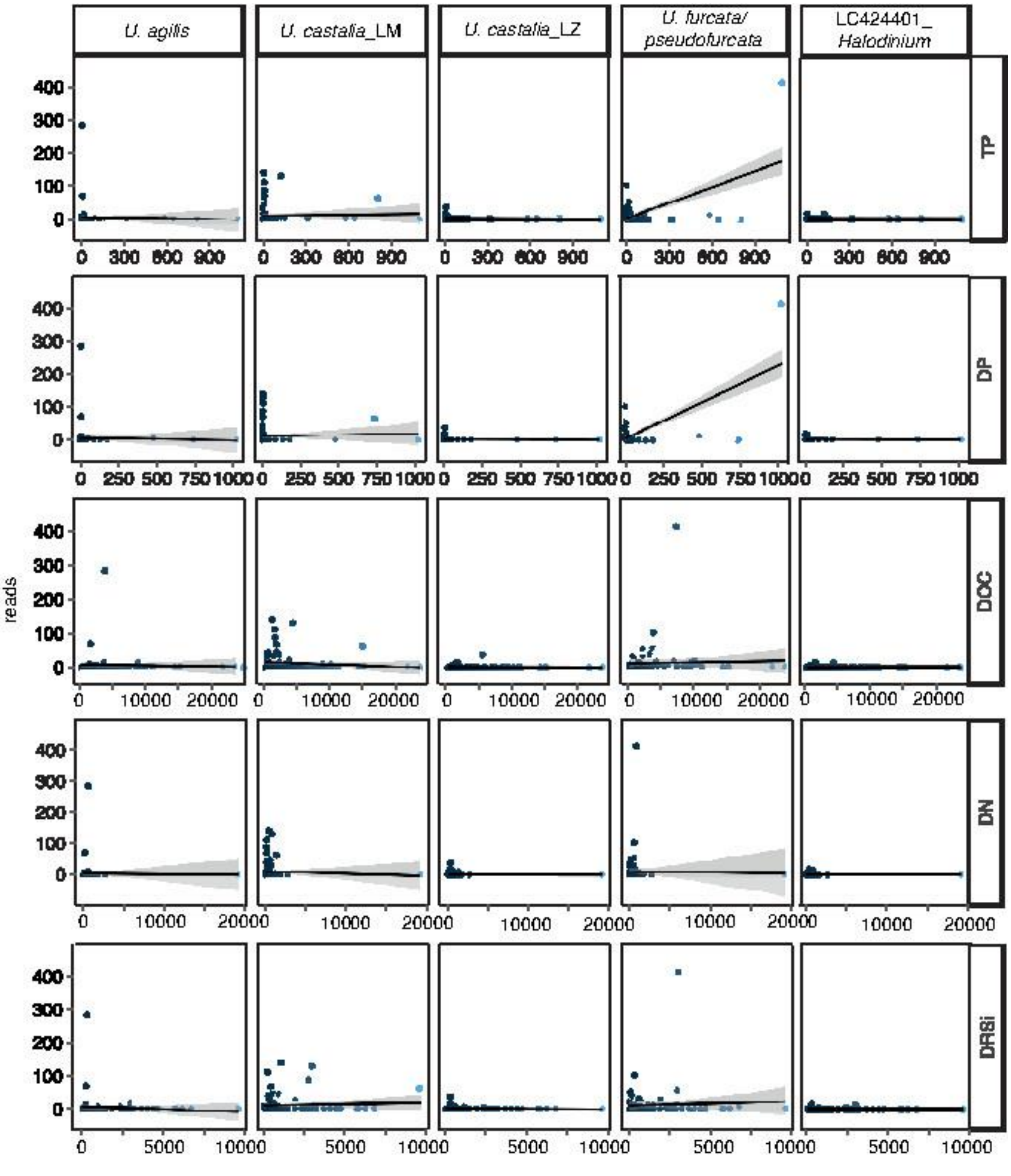

Supplementary Figure 7 | Ecological preferences of the investigated ciliate strains. Read abundances were correlated with and plotted against different environmental parameters. The black lines are regression lines and the gray area indicates the 95%-confidence intervals. Correlations were considered significant when the *p*-value was <0.05.

Supplementary Table 1 | Valid names of *Urotricha* species according to Aescht (2012). Doubtful species were excluded (for details, see Foissner and Pfister, 1997).

**Accepted name**	**Possible synonym**	**Comments**	**Caudal Cilia #**	**Habitat**
Urotricha agilis (Stokes, 1886) Kahl, 1930	*Urotricha gyrans* (Stokes, 1887) Foissner. 1979		1	freshwater
	Urotricha nais Muñoz et al., 1987		1	freshwater
Urotricha baikalensis Alekperov et al., 2012			1	freshwater
Urotricha corlissiana Song and Wilbert, 1989	*Urotricha platystoma* Stokes, 1886		1	freshwater
Urotricha discolor Kahl, 1930			1	freshwater
Urotricha dragescoi Foissner, 1984	*Urotricha armata* Kahl, 1927 sensu Dragesco et al., 1974		1	freshwater
Urotricha farcta Claparède and Lachmann, 1859	*Urotricha fareta* Claparède and Lachmann, 1859 (misspelling)		1	freshwater
	Urotricha gyrans (Stokes, 1887) Foissner, 1979		1	freshwater
	Urotricha minkewicki Schouteden, 1906		1	freshwater
	Urotricha minkewickzi Schouteden, 1906 (misspelling)		1	freshwater
	Urotricha parvula Penard, 1922		1	freshwater
Urotricha globosa Schewiakoff, 1892			1	freshwater
Urotricha gracilis Penard, 1922			1	freshwater
Urotricha lagenula Ehrenberg-Kent, 1881			1	freshwater
Urotricha lemani Foissner et al., 1994	*Urotricha armata* Dragesco, 1960	*Urotricha lemani* is the replacement name for the junior primary homonym *U. armata* Dragesco, 1960	1	freshwater
Urotricha nais Muñoz et al., 1987	Probably synonym of *Urotricha agilis* (Stokes, 1886) Kahl, 1930		1	freshwater
Urotricha ondina Muñoz et al., 1989			1	freshwater
Urotricha ovata Kahl, 1926	Possibly, a synonym of *Urotricha farcta* Claparède and Lachmann, 1859		1	freshwater
Urotricha platystoma Stokes, 1886	*Urotricha armata*, Kahl, 1927		1	freshwater
	Urotricha corlissiana Song and Wilbert, 1989		1	freshwater
Urotricha psenneri Sonntag and Foissner, 2004			1	freshwater

Urotricha pusilla Penard, 1922		Kahl (1930) doubted that this species was an *Urotricha*	1	freshwater
Urotricha ristoi Krainer, 1995			1	freshwater
Urotricha sphaerica Grolière, 1977			1	freshwater
Urotricha synuraphaga Kahl, 1927			1	freshwater
Urotricha vitrea Martín-Gonzalez et al., 1985			1	freshwater
Urotricha furcata Schewiakoff, 1892			2	freshwater
Urotricha macrostoma Foissner, 1983			2	freshwater
Urotricha pseudofurcata Krainer, 1995			2	freshwater
Urotricha cyrtonucleata Martin and Montagnes, 1993			3	marine, brackish
Urotricha spetai Foissner, 2012			4	freshwater
Urotricha matthesi matthesi Krainer, 1995	*Urotricha matthesi*, Krainer, 1995		3-4	freshwater
Urotricha matthesi tristicha Foissner and Pfister, 1997			4-5	freshwater
Urotricha tricha Wang and Nie, 1933			4, rarely 5-6	freshwater
Urotricha castalia Muñoz et al., 1987	*Urotricha rotunda*, Fernandez-Leborans and Novillo, 1994		4-9	freshwater
Urotricha antarctica Wilbert and Song, 2008			ca. 6	marine, brackish
Urotricha multisetosa Wang & Nie, 1933	*Urotricha faurei* Dragesco et al., 1974		At least 10	freshwater
Urotricha apsheronica Alekperov, 1984			12-16	freshwater
Urotricha pelagica Kahl, 1935		Redescribed by Foissner and Pfister (1997) who mentioned high similarity to *U. apsheronica*	14-18	freshwater
Urotricha terricola Alekperov and Musayev, 1988		Redescription required according to Foissner and Pfister (1997)	16	soil
Urotricha simonsbergeri Foissner et al., 1999			ca. 25	freshwater
Urotricha venatrix (Kahl, 1935) Foissner and Pfister, 1997			27-35	freshwater

Supplementary Table 4 | Main morphometric data on the investigated *Urotricha* strains. Measurements in µm. CV = coefficient of variation in %, M = median, IV = *in vivo*, Max = maximum, Mean = arithmetic mean, Meth = applied methods, Min = minimum, n = number of specimens investigated, P = after protargol staining, QPS = after quantitative protargol staining, SD = standard deviation. Corresponding strains are color-coordinated. *Urotricha agilis* (CIL-2017/24, CIL-2019/10), *Urotricha furcata* (CIL-2019/13 and CIL-2019/6), *Urotricha pseudofurcata* (CIL-2019/3), and *Urotricha castalia* (CIL-2017/25, CIL-2017/27, and CIL-2019/1; CIL-48 is the original monoculture of *U. castalia* of which the clonal strain CIL-2019/1 derived).

Character	Strain	Meth.	Mean	M	SD	SE	CV	Min	Max	n
Cell, length	CIL-2017/24	IV	17.9	18.1	2.0	0.4	10.9	14.1	22.8	21
		P	13.8	13.9	1.3	0.2	9.3	10.3	16.3	33
	CIL-2019/10	IV	16.4	16.5	1.7	0.4	10.4	13.3	19.2	21
		P	11.4	11.0	1.5	0.3	13.2	8.7	14.5	21
	CIL-2019/13	IV	22.4	21.7	3.9	0.8	17.2	16.6	35.2	21
	CIL-2019/6	P	16.0	16.3	2.0	0.4	12.7	10.7	18.5	23
	CIL-2019/3	IV	19.8	19.9	2.3	0.5	11.4	15.7	23.8	21
		P	12.8	12.2	2.4	0.5	19.1	8.5	19.8	23
	CIL-2017/25	IV	38.7	38.9	5.6	1.2	14.5	26.1	50.6	21
		P	21.8	22.1	2.8	0.6	12.7	17.7	28.2	23
	CIL-2017/27	IV	49.1	48.7	6.2	1.4	12.6	37.9	59.2	21
		P	23.6	23.9	3.4	0.7	14.5	17.0	29.0	23
	CIL-2019/1	IV	39.7	39.9	7.7	1.7	19.3	25.4	53.3	21
		P	30.1	29.1	4.1	0.8	13.7	24.4	41.5	27
Cell, width	CIL-2017/24	IV	11.7	11.7	1.8	0.4	15.3	8.9	16.1	21
		P	10.6	10.5	1.6	0.3	14.9	8.0	13.5	33
	CIL-2019/10	IV	11.8	11.6	1.4	0.3	12.1	9.1	14.4	21
		P	8.0	8.0	1.3	0.3	16.4	6.0	11.0	21
	CIL-2019/13	IV	14.7	14.3	3.7	0.8	25.1	10.3	28.5	21
	CIL-2019/6	P	13.2	13.4	2.0	0.4	15.5	10.4	16.3	23
	CIL-2019/3	IV	13.7	12.7	2.4	0.5	17.9	10.4	18.5	21
		P	9.3	8.6	2.2	0.5	23.8	6.2	14.2	23
	CIL-2017/25	IV	31.4	30.3	4.7	1.0	14.9	24.9	43.8	21
		P	15.8	15.7	2.0	0.4	12.4	13.3	19.4	23
	CIL-2017/27	IV	35.9	37.3	6.2	1.4	17.4	25.1	43.6	21
		P	17.8	17.6	2.4	0.5	13.3	14.1	22.9	23
	CIL-2019/1	IV	35.5	35.8	6.2	1.4	17.5	25.5	46.4	21
		P	25.9	25.4	4.1	0.8	15.9	20.7	37.6	27
Cell length: width, ratio	CIL-2017/24	IV	1.5	1.5	0.2	0.0	10.9	1.2	1.9	21
		P	1.3	1.3	0.2	0.0	12.0	1.0	1.6	33
	CIL-2019/10	IV	1.4	1.4	0.2	0.0	14.1	1.1	1.9	21
		P	1.5	1.4	0.2	0.0	12.6	1.1	1.8	21
	CIL-2019/13	IV	1.5	1.5	0.2	0.0	11.1	1.2	1.9	21
	CIL-2019/6	P	1.2	1.2	0.2	0.0	15.5	0.9	1.7	23
	CIL-2019/3	IV	1.5	1.5	0.2	0.0	11.0	1.2	1.8	21
		P	1.4	1.4	0.1	0.0	10.0	1.2	1.8	23
	CIL-2017/25	IV	1.2	1.2	0.2	0.0	14.0	0.8	1.6	21
		P	1.4	1.4	0.2	0.0	12.3	1.1	1.8	23
	CIL-2017/27	IV	1.4	1.3	0.2	0.0	12.9	1.2	1.8	21
		P	1.3	1.3	0.1	0.0	10.1	1.1	1.6	23
	CIL-2019/1	IV	1.1	1.2	0.2	0.0	17.6	0.8	1.4	21
		P	1.2	1.1	0.1	0.0	7.8	1.1	1.4	27
Distance between circumoral kinety and somatic kineties	CIL-2017/24	IV	-	-	-	-	-	-	-	-

		P	2.3	2.0	0.8	0.2	35.9	0.5	4.0	21
	CIL-2019/10	IV	-	-	-	-	-	-	-	-
		P	2.3	2.0	0.4	0.1	17.3	1.8	3.0	21
	CIL-2019/13	IV	-	-	-	-	-	-	-	-
	CIL-2019/6	P	1.0	1.0	0.5	0.1	51.6	0.0	1.5	21
	CIL-2019/3	IV	-	-	-	-	-	-	-	-
		P	1.2	1.0	1.3	0.1	24.7	1.0	2.0	21
	CIL-2017/25	IV	-	-	-	-	-	-	-	-
		P	0.6	0.5	0.4	0.1	61.0	0.1	1.0	21
	CIL-2017/27	IV	-	-	-	-	-	-	-	-
		P	0.8	1.0	0.3	0.1	36.0	0.3	1.0	7
	CIL-2019/1	IV	-	-	-	-	-	-	-	-
		P	1.0	1.0	0.3	0.1	34.8	0.5	2.0	21
Unciliated posterior portion, length	CIL-2017/24	IV	-	-	-	-	-	-	-	-
		P	3.5	3.4	0.8	0.2	23.1	2.5	5.7	21
	CIL-2019/10	IV	-	-	-	-	-	-	-	-
		P	4.2	4.0	0.6	0.1	13.3	3.0	5.0	19
	CIL-2019/13	IV	-	-	-	-	-	-	-	-
	CIL-2019/6	P	3.4	3.5	0.6	0.1	18.3	2.3	4.4	21
	CIL-2019/3	IV	-	-	-	-	-	-	-	-
		P	3.5	3.2	1.5	0.3	42.3	1.0	9.0	21
	CIL-2017/25	IV	10.1	9.1	2.5	0.5	24.9	6.8	15.0	21
		P	4.2	4.3	0.8	0.2	18.1	3.0	5.7	21
	CIL-2017/27	IV	-	-	-	-	-	-	-	-
		P	4.1	3.9	1.0	0.2	25.2	2.4	6.4	21
	CIL-2019/1	IV	-	-	-	-	-	-	-	-
		P	6.1	6.3	1.5	0.4	24.0	3.4	9.3	11
Caudal cilia, length	CIL-2017/24	IV	9.0	8.7	1.7	0.4	19.3	6.3	12.1	21
		P	7.3	7.4	1.4	0.3	18.9	5.2	9.7	21
	CIL-2019/10	IV	6.8	6.8	1.0	0.2	15.3	4.8	8.7	21
		P	7.0	7.0	0.7	0.1	9.7	6.0	8.5	21
	CIL-2019/13	IV	7.9	7.7	1.3	0.3	15.9	5.5	10.8	21
	CIL-2019/6	P	6.0	5.9	1.1	0.2	19.1	4.0	8.0	21
	CIL-2019/3	IV	6.4	6.3	1.1	0.2	16.5	4.8	8.8	21
		P	6.1	5.7	1.2	0.3	19.9	4.5	8.7	21
	CIL-2017/25	IV	9.4	9.3	1.1	0.2	12.0	7.3	12.6	21
		P	5.9	6.0	1.1	0.2	19.4	3.1	8.5	21
	CIL-2017/27	IV	8.4	8.6	0.9	0.2	10.7	6.7	9.8	21
		P	6.4	6.7	1.1	0.2	16.6	4.6	8.3	21
	CIL-2019/1	IV	8.0	8.3	1.5	0.3	19.2	4.3	10.7	21
		P	5.9	5.1	1.6	0.3	27.3	4.4	10.6	21
Caudal cilia, number	CIL-2017/24	IV	1.0	1.0	0.0	0.0	0.0	1.0	1.0	21
		P	1.0	1.0	0.0	0.0	0.0	1.0	1.0	21
	CIL-2019/10	IV	1.0	1.0	0.0	0.0	0.0	1.0	1.0	21
		P	1.0	1.0	0.0	0.0	0.0	1.0	1.0	21
	CIL-2019/13	IV	2.0	2.0	0.0	0.0	0.0	2.0	2.0	21
	CIL-2019/6	P	2.0	2.0	0.0	0.0	0.0	2.0	2.0	21
	CIL-2019/3	IV	2.0	2.0	0.0	0.0	0.0	2.0	2.0	21
		P	2.0	2.0	0.0	0.0	0.0	2.0	2.0	21
	CIL-2017/25	IV	4.5	4.0	0.8	0.2	18.2	4.0	7.0	21
		P	5.1	5.0	1.0	0.2	19.5	4.0	7.0	21
	CIL-2017/27	IV	4.4	4.0	0.8	0.2	18.3	4.0	7.0	21
		P	6.0	6.0	1.1	0.2	18.0	4.0	7.0	21
	CIL-2019/1	IV	4.7	4.0	0.9	0.2	19.6	4.0	7.0	21

		P	5.4	5.0	1.3	0.3	23.3	4.0	7.0	19
Somatic cilia, length	CIL-2017/24	IV	4.5	4.6	0.8	0.2	17.7	3.0	6.2	21
		P	4.4	4.3	0.9	0.2	19.9	2.9	6.5	21
	CIL-2019/10	IV	4.1	4.2	1.0	0.2	24.7	2.1	6.3	21
		P	5.5	6.0	0.8	0.2	15.1	4.0	6.5	21
	CIL-2019/13	IV	5.6	5.9	1.0	0.2	17.1	3.2	6.8	21
	CIL-2019/6	P	4.1	4.0	0.8	0.2	19.5	2.6	5.7	23
	CIL-2019/3	IV	4.1	4.2	0.5	0.1	13.2	2.8	5.1	21
		P	4.1	4.1	0.7	0.2	17.1	3.1	6.0	21
	CIL-2017/25	IV	-	-	-	-	-	-	-	-
		P	3.7	3.7	0.6	0.1	16.0	2.8	5.1	21
	CIL-2017/27	IV	-	-	-	-	-	-	-	-
		P	3.7	3.6	0.9	0.2	24.0	2.3	5.8	21
	CIL-2019/1	IV	5.7	5.4	1.6	0.3	27.8	3.6	8.6	21
		P	3.6	3.5	0.7	0.2	19.2	2.2	4.8	21
Somatic kineties, number	CIL-2017/24	IV	-	-	-	-	-	-	-	-
		P	21.6	22.0	1.6	0.4	7.5	18.0	24.0	21
	CIL-2019/10	IV	-	-	-	-	-	-	-	-
		P	17.7	18.0	2.2	0.5	12.5	14.0	22.0	21
	CIL-2019/13	IV	-	-	-	-	-	-	-	-
	CIL-2019/6	P	21.7	22.0	1.7	0.4	8.0	18	24	21
	CIL-2019/3	IV	-	-	-	-	-	-	-	-
		P	19.2	18.0	2.7	0.6	14.1	14.0	24.0	21
	CIL-2017/25	IV	-	-	-	-	-	-	-	-
		P	35.8	35.0	2.1	0.5	5.8	34.0	42.0	21
	CIL-2017/27	IV	36.0	-	0.0	0.0	0.0	36.0	36.0	2
		P	36.0	36.0	2.9	0.6	8.0	32.0	42.0	21
	CIL-2019/1	IV	39.0	40.0	2.0	0.7	5.1	34.0	40.0	9
		P	38.2	-	3.3	0.7	8.8	34.0	46.0	20
Cilia in an anteriorly unshortened somatic kinety, number	CIL-2017/24	IV	9.1	9.0	1.2	0.3	13.0	7.0	12.0	21
		P	8.8	8.0	1.2	0.3	13.9	6.0	11.0	21
	CIL-2019/10	IV	-	-	-	-	-	-	-	-
		P	5.8	6.0	0.8	0.2	14.4	5.0	7.0	21
	CIL-2019/13	IV	-	-	-	-	-	-	-	-
	CIL-2019/6	P	11.4	11.0	0.9	0.2	7.9	10.0	14.0	21
	CIL-2019/3	IV	-	-	-	-	-	-	-	-
		P	9.6	9.0	1.7	0.4	18.0	8.0	14.0	21
	CIL-2017/25	IV	-	-	-	-	-	-	-	-
		P	18.0	18.0	3.2	0.7	17.7	13.0	27.0	21
	CIL-2017/27	IV	-	-	-	-	-	-	-	-
		P	19.0	19.0	2.8	0.6	14.9	13.0	27.0	21
	CIL-2019/1	IV	-	-	-	-	-	-	-	-
		P	21.2	-	3.1	0.7	14.6	18.0	30.0	18
Adoral organelle1, length	CIL-2017/24	IV	-	-	-	-	-	-	-	-
		P	2.0	2.0	0.2	0.0	11.5	1.5	2.5	21
	CIL-2019/10	IV	-	-	-	-	-	-	-	-
		P	2.1	2.0	0.3	0.1	12.7	2.0	3.0	21
	CIL-2019/13	IV	-	-	-	-	-	-	-	-
	CIL-2019/6	P	1.9	2.0	0.4	0.1	22.3	1.0	2.5	17
	CIL-2019/3	IV	-	-	-	-	-	-	-	-

		P	2.0	2.0	0.2	0.0	11.2	1.5	2.5	21
	CIL-2017/25	IV	-	-	-	-	-	-	-	-
		P	1.9	2.0	0.2	0.0	11.6	1.5	2.0	21
	CIL-2017/27	IV	-	-	-	-	-	-	-	-
		P	2.0	-	0.3	0.1	16.5	1.2	2.5	14
	CIL-2019/1	IV	-	-	-	-	-	-	-	-
	+ CIL-48	P+QPS	2.4	-	0.4	0.1	17.8	2.0	3.0	14
Adoral organelle 2, length	CIL-2017/24	IV	-	-	-	-	-	-	-	-
		P	0.9	-	0.1	0.0	12.2	0.8	1.2	18
	CIL-2019/10	IV	-	-	-	-	-	-	-	-
		P	1.0	1.0	0.0	0.0	0.0	1.0	1.0	5
	CIL-2019/13	IV	-	-	-	-	-	-	-	-
	CIL-2019/6	P	1.1	-	0.2	0.1	21.3	0.8	1.5	10
	CIL-2019/3	IV	-	-	-	-	-	-	-	-
		P	1.4	1.5	0.2	0.1	13.2	1.0	1.5	7
	CIL-2017/25	IV	-	-	-	-	-	-	-	-
		P	1.5	1.5	0.2	0.0	14.6	1.2	2.0	21
	CIL-2017/27	IV	-	-	-	-	-	-	-	-
		P	1.3	1.2	0.3	0.1	23.5	1.0	2.0	9
	CIL-2019/1	IV	-	-	-	-	-	-	-	-
	+ CIL-48	P+QPS	1.7	-	0.3	0.1	18.8	1.0	2.0	14
Adoral organelle 3, length	CIL-2017/24	IV	-	-	-	-	-	-	-	-
		P	0.9	-	0.1	0.0	10.7	0.8	1.0	14
	CIL-2019/10	IV	-	-	-	-	-	-	-	-
		P	1.0	1.0	0.0	0.0	0.0	1.0	1.0	3
	CIL-2019/13	IV	-	-	-	-	-	-	-	-
	CIL-2019/6	P	1.0	-	0.1	0.0	8.4	0.8	1.0	6
	CIL-2019/3	IV	-	-	-	-	-	-	-	-
		P	-	-	-	-	-	1.5	1.5	1
	CIL-2017/25	IV	-	-	-	-	-	-	-	-
		P	0.9	1.0	0.2	0.0	20.9	0.2	1.2	19
	CIL-2017/27	IV	-	-	-	-	-	-	-	-
		P	0.9	-	0.3	0.1	28.6	0.5	1.0	4
	CIL-2019/1	IV	-	-	-	-	-	-	-	-
	+ CIL-48	P+QPS	1.0	-	0.3	0.1	31.9	0.5	1.5	14
Dikinetids in adoral organelle 1, number	CIL-2017/24	IV	-	-	-	-	-	-	-	-
		P	4.1	4.0	0.4	0.1	10.7	3.0	5.0	21
	CIL-2019/10	IV	-	-	-	-	-	-	-	-
		P	4.0	4.0	0.2	0.0	5.4	4.0	5.0	21
	CIL-2019/13	IV	-	-	-	-	-	-	-	-
	CIL-2019/6	P	3.7	4.0	0.5	0.1	13.0	3.0	4.0	19
	CIL-2019/3	IV	-	-	-	-	-	-	-	-
		P	4.0	4.0	0.3	0.1	7.9	3.0	5.0	21
	CIL-2017/25	IV	-	-	-	-	-	-	-	-
		P	4.0	4.0	0.0	0.0	0.0	4.0	4.0	21
	CIL-2017/27	IV	-	-	-	-	-	-	-	-
		P	4.0	-	0.0	0.0	0.0	4.0	4.0	14
	CIL-2019/1	IV	-	-	-	-	-	-	-	-
	+ CIL-48	P+QPS	4.5	-	0.5	0.1	11.5	4.0	5.0	14
Dikinetids in adoral organelle 2, number	CIL-2017/24	IV	-	-	-	-	-	-	-	-
		P	2.1	-	0.2	0.1	11.5	2.0	3.0	18

	CIL-2019/10	IV	-	-	-	-	-	-	-	-
		P	2.0	2.0	0.0	0.0	0.0	2.0	2.0	5
	CIL-2019/13	IV	-	-	-	-	-	-	-	-
	CIL-2019/6	P	2.4	-	0.5	0.1	21.3	2.0	3.0	12
	CIL-2019/3	IV	-	-	-	-	-	-	-	-
		P	2.8	-	0.5	0.3	18.2	2.0	3.0	4
	CIL-2017/25	IV	-	-	-	-	-	-	-	-
		P	3.0	3.0	0.0	0.0	0.0	3.0	3.0	21
	CIL-2017/27	IV	-	-	-	-	-	-	-	-
		P	3.0	3.0	0.0	0.0	0.0	3.0	3.0	9
	CIL-2019/1	IV	-	-	-	-	-	-	-	-
	+ CIL-48	P+QPS	3.2	-	0.4	0.1	13.2	3.0	4.0	14
Dikinetids in adoral organelle 3, number	CIL-2017/24	IV	-	-	-	-	-	-	-	-
		P	1.9	2.0	0.3	0.1	13.4	1.0	2.0	15
	CIL-2019/10	IV	-	-	-	-	-	-	-	-
		P	2.0	2.0	0.0	0.0	0.0	2.0	2.0	3
	CIL-2019/13	IV	-	-	-	-	-	-	-	-
	CIL-2019/6	P	2.0	2.0	0.0	0.0	0.0	2.0	2.0	7
	CIL-2019/3	IV	-	-	-	-	-	-	-	-
		P	-	-	-	-	-	3.0	3.0	1
	CIL-2017/25	IV	-	-	-	-	-	-	-	-
		P	2.0	2.0	0.3	0.1	16.7	1.0	3.0	19
	CIL-2017/27	IV	-	-	-	-	-	-	-	-
		P	2.0	-	0.0	0.0	0.0	2.0	2.0	4
	CIL-2019/1	IV	-	-	-	-	-	-	-	-
	+ CIL-48	P+QPS	2.4	-	0.5	0.1	21.1	2.0	3.0	14
Circumoral dikinetids/oral flaps, number	CIL-2017/24	IV	-	-	-	-	-	-	-	-
		P	8.2	8.0	0.5	0.1	6.2	7.0	9.0	21
	CIL-2019/10	IV	-	-	-	-	-	-	-	-
		P	8.9	9.0	0.8	0.2	9.0	8.0	10.0	21
	CIL-2019/13	IV	-	-	-	-	-	-	-	-
	CIL-2019/6	P	10.0	10.0	1.3	0.3	13.2	7.0	12.0	21
	CIL-2019/3	IV	-	-	-	-	-	-	-	-
		P	10.2	10.0	1.1	0.2	11.1	8.0	12.0	21
	CIL-2017/25	IV	-	-	-	-	-	-	-	-
		P	17.8	18.0	1.4	0.3	7.7	15.0	21.0	21
	CIL-2017/27	IV	-	-	-	-	-	-	-	-
		P	16.6	17.0	1.3	0.3	7.7	15.0	19.0	21
	CIL-2019/1	IV	-	-	-	-	-	-	-	-
		P	23.0	22.0	2.7	0.6	12.0	19.0	29.0	21
Oral flaps, length	CIL-2017/24	IV	-	-	-	-	-	-	-	-
		P	1.7	1.7	0.6	0.1	32.0	1.2	3.8	21
	CIL-2019/10	IV	-	-	-	-	-	-	-	-
		P	1.4	1.3	0.2	0.1	16.8	1.2	2.1	21
	CIL-2019/13	IV	-	-	-	-	-	-	-	-
	CIL-2019/6	P	2.0	2.1	0.4	0.1	21.8	1.2	2.9	23
	CIL-2019/3	IV	-	-	-	-	-	-	-	-
		P	2.0	1.8	0.7	0.1	33.0	0.9	3.3	21
	CIL-2017/25	IV	-	-	-	-	-	-	-	-
		P	2.2	2.2	0.3	0.1	14.6	1.6	2.8	21
	CIL-2017/27	IV	-	-	-	-	-	-	-	-
		P	2.0	1.9	0.3	0.1	14.2	1.5	2.7	21

	CIL-2019/1	IV	-	-	-	-	-	-	-	-
		P	2.6	2.4	0.6	0.1	21.3	1.8	3.9	21
Macronucleus, length	CIL-2017/24	IV	4.3	4.3	1.1	0.2	25.0	2.7	7.7	21
		P	4.2	3.8	1.0	0.2	24.0	2.4	6.2	21
	CIL-2019/10	IV	3.9	3.8	1.0	0.2	25.9	1.8	6.3	21
		P	2.9	3.0	0.8	0.2	28.8	1.5	5.0	21
	CIL-2019/13	IV	5.0	4.8	0.8	0.2	15.4	4.0	6.7	21
	CIL-2019/6	P	5.3	5.4	0.9	0.2	17.9	3.4	6.6	21
	CIL-2019/3	IV	4.6	4.6	1.0	0.2	21.5	2.7	6.9	21
		P	5.0	4.8	1.0	0.3	19.9	3.7	7.2	9
	CIL-2017/25	IV	10.8	10.6	1.4	0.3	13.4	8.5	15.1	21
		P	8.0	8.0	1.6	0.3	19.9	5.3	10.9	21
	CIL-2017/27	IV	10.4	9.7	1.4	0.3	13.1	8.8	13.3	21
		P	7.8	7.2	1.7	0.4	21.8	5.3	12.1	21
	CIL-2019/1	IV	10.0	9.9	2.2	0.5	21.4	6.6	14.9	21
		P	9.1	-	1.8	0.4	20.3	6.5	13.6	24
Macronucleus, width	CIL-2017/24	IV	4.0	3.9	0.7	0.1	16.6	2.8	5.2	21
		P	4.1	4.0	1.0	0.2	23.8	2.7	5.8	21
	CIL-2019/10	IV	4.1	4.2	0.9	0.2	22.7	1.8	6.2	21
		P	3.2	3.0	1.0	0.2	32.2	1.0	5.0	21
	CIL-2019/13	IV	4.9	4.6	1.0	0.2	21.1	3.3	7.2	21
	CIL-2019/6	P	4.9	4.5	1.4	0.3	27.7	3.3	9.0	21
	CIL-2019/3	IV	4.5	4.5	1.1	0.2	24.4	2.5	6.8	21
		P	4.7	4.5	0.8	0.3	16.5	3.6	6.0	9
	CIL-2017/25	IV	8.9	8.7	1.4	0.3	15.6	7.3	12.5	21
		P	7.1	7.1	1.2	0.3	17.0	5.6	10.3	21
	CIL-2017/27	IV	10.7	10.8	1.9	0.4	18.1	8.2	13.4	21
		P	7.5	7.4	1.7	0.4	22.9	5.2	12.1	21
	CIL-2019/1	IV	9.5	9.7	1.2	0.3	13.1	6.9	12.1	21
		P	8.4	-	1.8	0.4	21.0	5.1	12.6	24
Anterior cell end to macronucleus, distance	CIL-2017/24	IV	-	-	-	-	-	-	-	-
		P	10.0	10.5	2.8	0.6	27.8	0.7	13.9	21
	CIL-2019/10	IV	-	-	-	-	-	-	-	-
		P	3.4	3.0	1.0	0.2	28.4	2.0	6.0	21
	CIL-2019/13	IV	-	-	-	-	-	-	-	-
	CIL-2019/6	P	6.3	6.0	2.3	0.5	35.8	2.0	11.0	21
	CIL-2019/3	IV	-	-	-	-	-	-	-	-
		P	4.4	5.0	1.0	0.2	23.4	3.0	7.0	21
	CIL-2017/25	IV	-	-	-	-	-	-	-	-
		P	8.5	8.0	3.4	0.7	40.1	5.0	20.0	21
	CIL-2017/27	IV	-	-	-	-	-	-	-	-
		P	8.8	8.0	3.2	0.7	35.9	4.0	15.0	21
	CIL-2019/1	IV	-	-	-	-	-	-	-	-
		P	9.4	9.0	4.3	0.9	45.5	4.0	19.0	21
Micronucleus, length	CIL-2017/24	IV	-	-	-	-	-	-	-	-
		P	-	-	-	-	-	-	-	-
	CIL-2019/10	IV	-	-	-	-	-	-	-	-
		P	-	-	-	-	-	-	-	-
	CIL-2019/13	IV	2.2	2.2	0.3	0.1	13.2	1.8	2.8	21
	CIL-2019/6	P	-	-	-	-	-	-	-	-

	CIL-2019/3	IV	1.7	1.7	0.3	0.1	19.0	0.9	2.3	21
		P	-	-	-	-	-	-	-	-
	CIL-2017/25	IV	-	-	-	-	-	-	-	-
		P	-	-	-	-	-	-	-	-
	CIL-2017/27	IV	3.0	-	1.3	0.9	44.6	2.0	3.9	2
		P	2.6	2.5	0.5	0.1	18.9	2.0	3.5	21
	CIL-2019/1	IV	2.7	2.5	0.8	0.2	29.8	1.5	4.7	21
		P	-	-	-	-	-	3.0	3.0	1
Micronucleus, width	CIL-2017/24	IV	-	-	-	-	-	-	-	-
		P	-	-	-	-	-	-	-	-
	CIL-2019/10	IV	-	-	-	-	-	-	-	-
		P	-	-	-	-	-	-	-	-
	CIL-2019/13	IV	2.2	2.1	0.3	0.1	13.6	1.6	2.7	21
	CIL-2019/6	P	-	-	-	-	-	-	-	-
	CIL-2019/3	IV	1.7	1.7	0.3	0.1	19.7	1.1	2.4	21
		P	-	-	-	-	-	-	-	-
	CIL-2017/25	IV	-	-	-	-	-	-	-	-
		P	-	-	-	-	-	-	-	-
	CIL-2017/27	IV	2.6	-	0.6	0.5	25.0	2.1	3.0	2
		P	2.0	2.0	0.4	0.1	19.9	1.5	3.0	21
	CIL-2019/1	IV	2.6	2.6	0.7	0.2	28.2	1.4	4.5	21
		P	-	-	-	-	-	2.0	2.0	1
Oral basket, distal diameter	CIL-2017/24	IV	4.0	3.8	1.2	0.3	29.4	2.2	6.9	21
		P	3.6	3.5	0.5	0.1	14.9	2.7	4.9	21
	CIL-2019/10	IV	-	-	-	-	-	-	-	-
		P	3.6	3.5	0.7	0.2	19.5	2.5	5.0	19
	CIL-2019/13	IV	-	-	-	-	-	-	-	-
	CIL-2019/6	P	4.2	4.1	0.6	0.1	14.5	3.2	5.7	21
	CIL-2019/3	IV								
		P	4.1	4.0	0.6	0.1	14.4	3.0	5.2	23
	CIL-2017/25	IV	-	-	-	-	-	-	-	-
		P	4.6	4.8	0.6	0.1	13.0	3.6	5.8	21
	CIL-2017/27	IV	-	-	-	-	-	-	-	-
		P	4.7	4.8	0.7	0.1	13.9	3.4	6.0	21
	CIL-2019/1	IV	12.7	11.7	3.9	0.8	30.4	5.7	18.7	21
		P	6.8	-	1.3	0.3	18.8	4.3	10.1	24
Oral basket, length	CIL-2017/24	IV	3.6	3.5	1.2	0.3	32.4	2.2	6.4	21
		P	8.4	8.0	1.5	0.3	17.5	5.9	11.5	21
	CIL-2019/10	IV	-	-	-	-	-	-	-	-
		P	-	-	-	-	-	-	-	-
	CIL-2019/13	IV	-	-	-	-	-	-	-	-
	CIL-2019/6	P	5.5	5.1	1.5	0.3	27.4	3.9	10.1	21
	CIL-2019/3	IV								
		P	8.4	9.0	2.5	0.8	30.2	3.9	12.0	21
	CIL-2017/25	IV	-	-	-	-	-	-	-	-
		P	10.1	9.5	2.2	0.5	21.8	6.8	15.1	21
	CIL-2017/27	IV	-	-	-	-	-	-	-	-
		P	10.3	9.5	2.4	0.5	23.0	7.2	17.6	21
	CIL-2019/1	IV	14.3	13.3	3.9	0.8	27.1	5.6	23.0	21
		P	11.9	-	2.0	0.4	17.1	8.3	16.2	24
Oral basket width:cell width, in %	CIL-2017/24	IV	0.3	0.4	0.1	0.0	30.4	0.2	0.6	21
		P	0.3	0.4	0.1	0.0	19.1	0.2	0.4	21

	CIL-2019/10	IV	0.3	0.3	0.1	0.0	20.2	0.2	0.3	3
		P	0.5	-	0.1	0.0	22.0	0.3	0.7	20
	CIL-2019/13	IV	-	-	-	-	-	-	-	-
	CIL-2019/6	P	0.3	0.3	0.0	0.0	14.0	0.2	0.4	21
	CIL-2019/3	IV	-	-	-	-	-	-	-	-
		P	0.4	0.4	0.1	0.0	26.8	0.3	0.8	21
	CIL-2017/25	IV	-	-	-	-	-	-	-	-
		P	0.3	0.3	0.0	0.0	13.6	0.2	0.4	21
	CIL-2017/27	IV	-	-	-	-	-	-	-	-
		P	0.3	0.3	0.0	0.0	13.8	0.2	0.3	21
	CIL-2019/1	IV	0.4	0.4	0.1	0.0	32.9	0.2	0.6	21
		P	0.3	0.3	0.0	0.0	14.4	0.2	0.3	21
Somatic extrusomes, type 1, length	CIL-2017/24	IV	-	-	-	-	-	1.4	1.4	1
		P	-	-	-	-	-	-	-	-
	CIL-2019/10	IV	-	-	-	-	-	1.5	1.5	1
		P	1.1	-	0.2	0.0	16.1	1.0	1.5	16
	CIL-2019/13	IV	-	-	-	-	-	-	-	-
	CIL-2019/6	P	-	-	-	-	-	-	-	-
	CIL-2019/3	IV	-	-	-	-	-	-	-	-
		P	0.9	0.8	0.2	0.0	25.2	0.6	1.5	21
	CIL-2017/25	IV	2.3	2.2	0.4	0.1	17.0	1.7	2.9	21
		P	-	-	-	-	-	-	-	-
	CIL-2017/27	IV	2.4	2.6	0.5	0.1	19.0	0.8	2.9	21
		P	-	-	-	-	-	-	-	-
	CIL-2019/1	IV	1.8	1.8	0.2	0.0	12.1	1.3	2.2	21
		P	-	-	-	-	-	-	-	-
Extrusomes type 2, restricted to unciliated posterior cell portion, length	CIL-2017/25	IV	4.9	4.8	0.6	0.1	12.7	4.0	6.2	18
		P	-	-	-	-	-	-	-	-
	CIL-2017/27	IV	4.4	-	0.7	0.2	15.2	3.4	5.4	18
		P	-	-	-	-	-	-	-	-
	CIL-2019/1	IV	4.1	4.1	0.7	0.1	16.4	3.0	5.3	21
		P	-	-	-	-	-	-	-	-
